# Helical Nanographenes Containing an Azulene Unit: Synthesis, Crystal Structures, and Properties

**DOI:** 10.1002/anie.201914716

**Published:** 2020-02-03

**Authors:** Ji Ma, Yubin Fu, Evgenia Dmitrieva, Fupin Liu, Hartmut Komber, Felix Hennersdorf, Alexey A. Popov, Jan J. Weigand, Junzhi Liu, Xinliang Feng

**Affiliations:** ^1^ Centre for Advancing Electronics Dresden (cfaed) Faculty of Chemistry and Food Chemistry Technische Universität Dresden 01062 Dresden Germany; ^2^ Leibniz Institute for Solid State and Materials Research 01069 Dresden Germany; ^3^ Leibniz-Institut für Polymerforschung Dresden e. V. Hohe Straße 6 01069 Dresden Germany; ^4^ Chair of Inorganic Molecular Chemistry Technische Universität Dresden 01062 Dresden Germany; ^5^ Department of Chemistry and State Key Laboratory of Synthetic Chemistry The University of Hong Kong Pokfulam Road Hong Kong China

**Keywords:** azulene, helicenes, nanographenes, polycyclic aromatic hydrocarbons, Scholl reaction

## Abstract

Three unprecedented helical nanographenes (**1**, **2**, and **3**) containing an azulene unit are synthesized. The resultant helical structures are unambiguously confirmed by X‐ray crystallographic analysis. The embedded azulene unit in **2** possesses a record‐high twisting degree (16.1°) as a result of the contiguous steric repulsion at the helical inner rim. Structural analysis in combination with theoretical calculations reveals that these helical nanographenes manifest a global aromatic structure, while the inner azulene unit exhibits weak antiaromatic character. Furthermore, UV/Vis‐spectral measurements reveal that superhelicenes **2** and **3** possess narrow energy gaps (**2**: 1.88 eV; **3**: 2.03 eV), as corroborated by cyclic voltammetry and supported by density functional theory (DFT) calculations. The stable oxidized and reduced states of **2** and **3** are characterized by in‐situ EPR/Vis–NIR spectroelectrochemistry. Our study provides a novel synthetic strategy for helical nanographenes containing azulene units as well as their associated structures and physical properties.

## Graphical abstract

**An odd contribution**: Azulene‐embedded helical nanographenes with global aromaticity are synthesized by a Scholl‐type cyclization. X‐ray crystallographic analysis clearly reveals the formation of an azulene unit in the helical π‐system. The embedded azulene core adopts a highly twisted conformation and is less aromatic than pristine azulene.

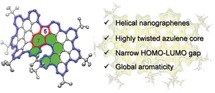

Contorted polycyclic aromatic hydrocarbons (PAHs, or nanographenes) have received considerable attention in the past decades because of their intriguing optoelectronic properties and applications in organic electronics.^1^, ^2^, ^3^, ^4^, ^5^ Among the contorted PAHs, helicenes represent an important class of compounds because of their unique nonplanarity, inherent chirality, and dynamic behavior.^6^ Recently, embedding helicene moieties into large polycyclic aromatic systems has emerged as an important strategy for achieving nanographenes with interesting chemical bonding, aromaticity, and chirality (Figure 1 a).^7^, ^8^, ^9^, ^10^ Importantly, such helical nanographenes greatly alleviate intermolecular aggregation due to their highly twisted geometry and exhibit unique optoelectronic properties, enabling potential applications in circular dichroism,^11^ chiral‐induced spin selectivity,^12^ and nonlinear optics.^13^ In addition to the π‐extended helical aromatics consisting solely of hexagons, the incorporation of nonhexagonal rings in such π‐systems can lead to the formation of positively or negatively helical PAHs with an exotic molecular geometry and superior photophysical properties with respect to planar PAHs.^14^, ^15^, ^16^, ^17^, ^18^ For instance, the pentagon‐embedded bowl‐helix hybrid molecule (Figure 1 a) exhibits inversion motions of both the bowl and helix in its enantiomerization processes,^15^ while the heptagon‐embedded saddle‐helix hybrid nanographene displays enhanced nonlinear optics and chiroptical properties.^19^

**Figure 1:**
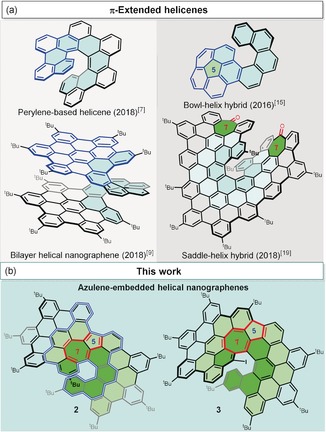
Structures of a) π‐extended helicenes and b) π‐extended azulene‐embedded helical nanographenes (**2** and **3**) reported in this work. The structure of **1** is indicated by a blue double line in **2**.

In contrast to individual odd‐membered rings, azulene, which consists of a pair of five‐ and seven‐membered rings, is a non‐benzenoid, non‐alternant aromatic hydrocarbon.^20^, ^21^ It should be noted that the topological transformation of naphthalene into azulene results in a large perturbation of the molecular symmetry and physicochemical properties.^22^ Previous related studies have revealed that incorporation of non‐alternant units into PAHs is an efficient method to tailor their optoelectronic properties.^23^, ^24^, ^25^, ^26^ However, embedding the azulene unit into a π‐extended helical system is still only possible to a limited extent, mostly due to the lack of a facile synthetic route and the possible azulene‐to‐naphthalene rearrangement.^27^, ^28^

In this work, we demonstrate a novel synthetic strategy towards a class of unprecedented helical nanographenes containing an azulene unit (**1**–**3**) based on a Scholl‐type oxidative cyclization from spatially crowded biaryl precursors (Scheme 1). Single‐crystal X‐ray analysis unequivocally reveals the π‐extended helical structures of this family of azulene‐based nanographenes. Notably, the embedded azulene unit in **1**–**3** possesses a profound twisting degree due to the steric crowdedness derived from their helical structures; among them, **2** has a record value (16.1°) in comparison to that of previously reported azulene‐based PAHs.^26^, ^27^, ^29^ Furthermore, UV/Vis absorption and cyclic voltammetry (CV) analysis show narrow optical energy gaps (**2**: 1.88 eV; **3**: 2.03 eV) and amphoteric redox properties for **1**–**3**. Additionally, this class of helical nanographenes exhibits remarkable global aromaticity, while the inner azulene cores display weak antiaromaticity from the structural analysis, which is also supported by the theoretical calculations.

**Scheme 1:**
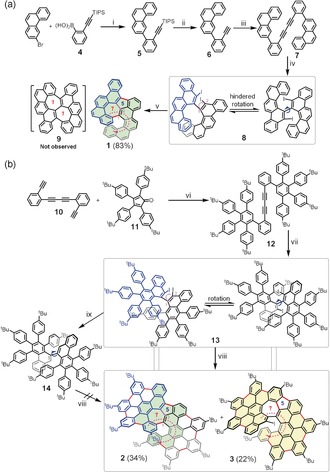
Synthesis of a) **1** and b) the π‐extended azulene‐embedded superhelicenes **2** and **3**. Reagents and conditions: i) Pd(PPh_3_)_4_, 2 m K_2_CO_3_, THF, 90 °C, 20 h, 91 %; ii) TBAF, THF, 1 h, quant.; iii) CuCl, piperidine, toluene, 60 °C, 3 h, 88 %; iv) ICl, DCM, −78 °C, 2 h, 44 %; v) DDQ, MSA, DCM, 0 °C, 30 min, 83 %; vi) *o*‐xylene, reflux, 12 h, 83 %; vii) ICl, DCM, −78 °C, 2 h, 85 %; viii) DDQ, TfOH, DCM, 0 °C, 30 min, 34 % (**2**) and 22 % (**3**); ix) *n*‐BuLi, THF, −78 °C, 1 h, 72 %.

The synthesis of **1** was carried out as shown in Scheme 1 a. First, triisopropyl((2‐(phenanthren‐3‐yl)phenyl)ethynyl)silane (**5**) was prepared by Suzuki coupling between (2‐((triisopropylsilyl)ethynyl)phenyl)boronic acid (**4**) and the commercially available 3‐bromophenanthrene. Subsequently, compound **5** was deprotected with TBAF to give **6** in quantitative yield. Then, a CuCl‐catalyzed Glaser self‐coupling of **6** gave 1,4‐bis(2‐(phenanthren‐3‐yl)phenyl)buta‐1,3‐diyne (**7**) in 88 % yield. Next, the key precursor (*S*)‐8,8′‐diiodo‐7,7′‐bibenzo[*c*]chrysene (**8**) was obtained in good yield (44 %) through the selective ICl‐induced cyclization of **7**, which was verified by single‐crystal structural analysis (Figure S8, Supporting Information). Initially, we intuitively considered that the projected heptalene‐embedded compound **9** would possibly be formed during the Scholl reaction from precursor **8**. To our surprise, azulene‐embedded compound **1** was obtained in high yield (83 %) by treatment of **8** with 2,3‐dichloro‐5,6‐dicyano‐1,4‐benzoquinone (DDQ) and methanesulfonic acid (MSA). The helical structure of **1** was unambiguously determined by X‐ray single‐crystal analysis. Additionally, we found that subjection of **8** to DDQ/triflic acid (TfOH) at 0 °C yielded unidentified side products. The successful formation of **1** can be attributed to the rotation of the 8‐iodobenzo[*c*]chrysene of **8** (highlighted in blue in Scheme 1 a) following HI elimination after the 1,2‐aryl migration during the Scholl‐type cyclization. The proposed mechanism is described in Scheme S3.^18^, ^30^

Inspired by the interesting result above, a larger and flexible precursor **13** in which the core of the molecular skeleton is similar to that of **8** was thus designed to synthesize the azulene‐embedded superhelicene **2** bearing helicene **1** (highlighted by light‐green shading in **2**, Scheme 1 b). Precursor **13** was obtained by ICl‐mediated benzannulation of diacetylene **12** in 85 % yield, which was synthesized through a Diels–Alder cycloaddition from 1,4‐bis(2‐ethynylphenyl)buta‐1,3‐diyne (**10**) and cyclopentadienone derivative **11**. Then, **13** was treated with DDQ/TfOH at 0 °C. There were two dominant red spots on the thin‐layer‐chromatography (TLC) plate after the reaction, which were effectively separated by preparative silica TLC after the standard work‐up. Single‐crystal X‐ray analysis clearly revealed the formation of π‐expanded helical nanographenes **2** and **3** containing an azulene unit (Figure 2). We attribute the same mechanism involved in the formation of **2** to that of **1**, where the rotation and 1,2‐migration of the substituted 9‐iodophenanthrene moiety in **13** (highlighted in blue, Scheme 1 b) occurred during the Scholl reaction. Regarding the formation of **3**, apart from eight C−C bonds formed between the peripheral phenyl rings in **13** (indicated by yellow shading in Scheme 1 b), a seven‐membered ring was formed through HI elimination, and a five‐membered ring was also established during the Scholl oxidation. The related mechanism for the formation of **2** and **3** via the Scholl reaction is proposed in Scheme S4.

**Figure 2:**
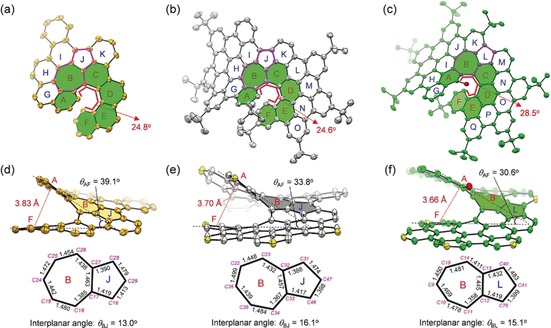
X‐ray crystallographic molecular structures of a) **1**, b) **2**, and c) **3**. Hydrogen atoms are omitted for clarity. Side view of the π‐extended helical geometry of d) **1**, e) **2**, and f) **3** as well as the bond lengths (in Å) of the embedded azulene unit (the *tert*‐butyl groups in **2** and **3** are removed for clarity, and the carbons that are bonded to *tert*‐butyl groups are shown in yellow).

To gain more insight into the Scholl reaction, the iodo substituents in **13** were removed using *n*‐BuLi, and the resultant compound **14** was subjected to the Scholl reaction using DDQ/TfOH. However, a complex and unpurifiable mixture was obtained from **14**. Therefore, this result suggests that the two iodo groups in precursor **13** play an important role in the formation of azulene‐embedded superhelicenes **2** and **3** during the Scholl reaction. All PAHs **1**–**3** have good solubility in common organic solvents, such as DCM, chloroform, THF, and toluene. Moreover, the identity of compounds **1**–**3** was further confirmed by high‐resolution mass spectrometry (Figures S1–S3) and NMR analysis (see the Supporting Information).

In the solid state, compounds **1**–**3** adopt a highly helical conformation, which is derived from the central heptagon‐embedded [6]helicene (filled with green color in Figure 2 a–c). Compound **1**, which can be regarded as a subunit of **2**, crystallizes in the *C*2/*c* space group with a pair of enantiomers (*M* and *P*; Figure S9 a). However, the π‐extended PAHs **2** and **3** crystallize in the space group $P\bar{1}$ . In the packing pattern, the intermolecular π–π distance of the enantiomeric pairs (*M* and *P*) in **3** is measured to be 3.28 Å (Figure S9), which is smaller than that in **1** (3.58 Å) and **2** (3.73 Å), suggesting a stronger intermolecular π–π stacking interaction in **3**. The distance between the centroids of the terminal rings A and F was measured to be 3.70 Å for **2** and 3.66 Å for **3** (Figure 2 e,f), which is significantly shorter than the value in compound **1** (3.83 Å; Figure 2 d) and [6]helicene (4.44 Å; Figure S10).^31^ Additionally, the splay angle between the two planes (*θ*_AF_) of the terminal rings in **2** and **3** was measured to be 33.8° and 30.6°, respectively, which is also significantly smaller than that in **1** (39.1°) and [6]helicene (58.4°; Figure S10). These structural analyses suggest an enhanced intramolecular π–π interaction in **2** and **3** with respect to **1** and pristine [6]helicene due to their extended π‐conjugation.^7^ The torsion angle along the helical inner rim directly reflects the geometrical distortion of the helical molecules. The mean value of the four torsion angles of **3** (28.5°) is significantly larger than the corresponding value in **1** (24.8°), **2** (24.6°), and [6]helicene (21.7°)^31^ (Figure 2 d–f), which can be attributed to the influence of the inner iodo substituent and the accumulative steric repulsion from ring G to the heptagon‐embedded [6]helicene moiety in **3**.

Another significant difference among **1**–**3** is the embedded azulene unit. As shown in Figure 2 f, there is almost no bond length alternation in the azulene unit of **3** since the C−C bonds in the seven‐membered ring (ring B) mainly exhibit C(sp^2^)−C(sp^2^) bond character. In **2**, however, significant double‐bond features (Figure 2 e) are determined for C31−C32 (1.388 Å), C33−C34 (1.367 Å), and C47−C48 (1.398 Å), which is reminiscent of the structural contribution from a pentafulvene unit.^27^ Similar to **2**, the C−C bond lengths of the azulene unit in **1** are between 1.39 and 1.48 Å without bond alternation (Figure 2 d). This observation suggests that the embedded azulene core has less aromatic character than pristine azulene. Intriguingly, the embedded azulene units in **1**–**3** are found to be highly twisted due to the steric crowdedness of the π‐extended helical structure (Figure 2 d–f). This remarkable conformation can be illustrated by the interplanar angles between the five and seven‐membered rings: The rings B and J in **2** form an angle of *θ*_BJ_=16.1°, while the rings B and L in **3** form an angle of *θ*_BL_=15.1°, which is larger than that in **1** (*θ*_BJ_=13.0°), representing a record‐high twisting degree of the reported azulene units. These parameters are in contrast with those of pristine azulene and other reported azulene‐embedded π‐systems in which the azulene moiety mainly adopts a planar structure.^26^, ^27^

The nucleus‐independent chemical shift (NICS) was calculated to evaluate the aromaticity of the π‐extended helical frameworks. According to the NICS calculations (Figures 3 d and S18), the embedded azulene units in **1**–**3** are slightly antiaromatic (**1**: B ring, +3.60 ppm, J ring, −0.01 ppm; **2**: B ring, +3.39 ppm, J ring, +0.39 ppm; **3**: B ring, +4.60 ppm, L ring, +1.94 ppm), while highly negative values were found at the surrounding hexagonal rings, which are consistent with the above bond‐length analysis of the azulene core. To further support the local aromaticity of the azulene‐embedded helical PAHs **1**–**3**, anisotropy of the induced current density (ACID) analysis was performed (Figure 3 a–c). Continuous counterclockwise paratropic ring currents appeared around the azulene unit in the ACID plots of **1**–**3**. However, diamagnetic ring currents were found in the six‐membered rings at the periphery. These results indicate that the azulene core in **1**–**3** displays weak local antiaromaticity, which is consistent with the results of the NICS calculations. Interestingly, the ACID plots exhibit the obvious clockwise ring current delocalized along the helical outer rim of the molecule, thus indicating the global aromaticity of **1**–**3**.

**Figure 3:**
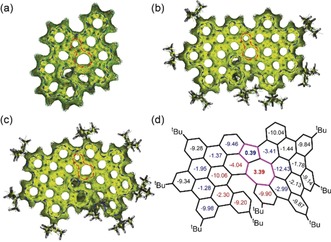
Calculated ACID plots for a) **1**, b) **2**, and c) **3**. The isovalue is 0.05, and the diamagnetic (clockwise) and paramagnetic (counterclockwise) ring currents under the magnetic field parallel to the *z*‐axis are highlighted by blue and red arrows, respectively. d) NICS(1) values of **2** calculated at GIAO‐B3LYP/6–31+G(2d,p).

To elucidate the optoelectronic properties of **1**–**3**, their UV/Vis absorption spectra were recorded in dry CH_2_Cl_2_. As shown in Figure 4 a, compound **1** features a broad absorption with a maximum at 474 nm, which is attributed to the HOMO→LUMO transition. Compared to **1**, π‐extended compound **2** displayed an intense red‐shifted absorption band at 536 nm, together with a weak absorption centered at 629 nm and a long tail up to 675 nm, which can be explained by an expansion of the π‐system. According to time‐dependent density functional theory (TD‐DFT) calculations, the absorption spectrum of **2** in the range of 510–650 nm can be attributed to a combination of the HOMO−1→LUMO and HOMO→LUMO transitions (Figure S17 and Table S3). However, the absorption spectrum of **3** exhibits a broad absorption with the longest‐wavelength absorption maximum at 573 nm and two shoulder peaks at 535 and 507 nm. TD‐DFT calculations [B3LYP/6‐31G(d)] attribute the absorption band at approximately 570 nm to the HOMO→LUMO and HOMO→LUMO+1 transitions (Figure S17 and Table S4). As estimated from the onsets of the lowest‐energy absorption band of their UV/Vis absorption spectra, the optical energy gaps of **1**, **2**, and **3** were calculated to be 2.31, 1.88, and 2.03 eV, respectively. Moreover, the CH_2_Cl_2_ solution of compound **1** exhibited yellow luminescence upon irradiation with UV light, and the emission spectrum of **1** displayed a maximum at 550 nm with two shoulders at 508 nm and 587 nm (Figure S11). However, regarding the red CH_2_Cl_2_ solutions of the π‐extended nanographenes **2** and **3**, there was no detectable fluorescence after excitation at their absorption maximum. To further study the molecular structures of **1**–**3**, Raman‐spectroscopical characterization was carried out (Figure S12). Compounds **1**–**3** displayed the typical D and G bands as a result of the alternate C=C/C−C vibrations in the molecules, which are consistent with the reported graphene molecules.^9^, ^16^

**Figure 4:**
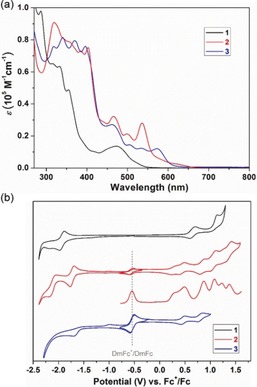
a) UV/Vis absorption spectra of **1**, **2**, and **3** in CH_2_Cl_2_ (1×10^−5^ mol L^−1^). b) CV of **1**, **2**, and **3** in CH_2_Cl_2_ containing 0.1 m nBu_4_NPF_6_ at a scan rate of 50 mV s^−1^. The square‐wave voltammogram of **2** was measured during the oxidation. DmFc (decamethylferrocene) was used as an internal standard (its redox potential is marked by a gray line).

Next, the electrochemical behavior of **1**–**3** was investigated by CV in deaerated CH_2_Cl_2_. As illustrated in Figure 4 b, compound **1** featured two oxidation waves at *E* ^ox^_1/2_=0.64 and 1.07 V (vs. Fc^+^/Fc), and two reduction waves at *E* ^red^_1/2_=−1.92 and −2.17 V. However, π‐extended compound **2** displayed six reversible oxidation waves with *E* ^ox^_1/2_ potentials at 0.31, 0.48, 0.87, 1.08, 1.23, and 1.37 V, and two reversible reduction waves with *E* ^red^_1/2_ at −1.69 and −2.09 V. In contrast, **3** manifested only two reversible oxidation waves with half‐wave potentials *E* ^ox^_1/2_ at 0.46 and 0.81 V, and one irreversible reduction wave at a peak potential of −1.69 V. Accordingly, the HOMO/LUMO levels were estimated to be −5.32/−2.99 eV, −4.99/−3.13 eV, and −5.18/−3.24 eV for **1**, **2**, and **3**, respectively, based on the onset potentials of the first oxidation/reduction waves. The electrochemical energy gaps (*E* ^EC^_g_) were thus calculated to be 2.33, 1.86, and 1.94 eV for **1**, **2**, and **3**, respectively, which are in good accordance with their optical energy gaps. Furthermore, no electron paramagnetic resonance (EPR) signals were detected in CH_2_Cl_2_ solutions of **2** or **3** at room temperature, indicating that both compounds are diamagnetic in their pristine form. In‐situ EPR/Vis–NIR spectroelectrochemistry measurements showed the formation of stable radical ions during the first oxidation process of **2** and **3** as well as the reduction process of **2** (Figures S14–S16). Interestingly, as found by the DFT calculations, the HOMOs and LUMOs of **1**–**3** are efficiently separated by the azulene unit and the frontier orbitals are respectively distributed on the two blades of the molecular backbone (Figure 5), suggesting the existence of intramolecular electron‐transfer behavior in **1**–**3**.^32^

**Figure 5:**
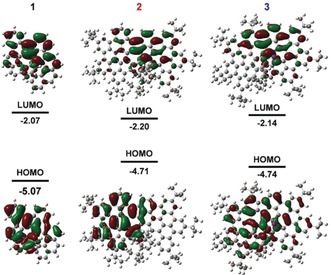
Frontier molecular orbitals and energy diagrams of **1**, **2**, and **3**, calculated at B3LYP/6‐31G(d).

In summary, we have reported an unprecedented synthetic strategy towards a series of helical nanographenes (**1**–**3**) containing a pentagon–heptagon pair through Scholl‐type oxidative cyclization from spatially crowded biaryl precursors (**8** and **13**). The single‐crystal X‐ray diffraction analysis unambiguously elucidated the structure of the embedded azulene unit in **1**–**3** and revealed the helically twisted geometry of this class of nanographenes. Compared to the planar and aromatic pristine azulene unit, the outstanding feature in compounds **1**–**3** is the inner non‐planar and slightly antiaromatic azulene core that results from the accumulated repulsions at the helical inner rim. Moreover, the π‐extended superhelicenes exhibit narrow optical energy band gaps and amphoteric redox properties as well as efficient HOMO–LUMO energy separation. The synthetic strategy reported herein not only stimulates the molecular design for unprecedented helical non‐alternant aromatics but also paves the way towards the development of azulene‐based helical/chiral nanographenes or graphene nanoribbons.^33^

## Conflict of interest

The authors declare no conflict of interest.

## Supporting information

As a service to our authors and readers, this journal provides supporting information supplied by the authors. Such materials are peer reviewed and may be re‐organized for online delivery, but are not copy‐edited or typeset. Technical support issues arising from supporting information (other than missing files) should be addressed to the authors.

Supplementary
