# Supplementary material for: Helical Nanographenes Containing an Azulene Unit: Synthesis, Crystal Structures, and Properties
Source: Angew Chem Int Ed Engl. 2020 Feb 3;59(14):5637–42. doi: 10.1002/anie.201914716 (PMC7155134; doi:10.1002/anie.201914716)
Supplement: Supplementary file 1 — Supplementary [file ANIE-59-5637-s001.pdf]

Angewandte Chemie

Eine Zeitschrift der Gesellschaft Deutscher Chemiker

Supporting Information

Helical Nanographenes Containing an Azulene Unit: Synthesis, Crystal Structures, and Properties

Ji Ma, Yubin Fu, Evgenia Dmitrieva, Fupin Liu, Hartmut Komber, Felix Hennersdorf, Alexey A. Popov, Jan J. Weigand, Junzhi Liu, and Xinliang Feng**

anie_201914716_sm_miscellaneous_information.pdf

Table of Content

1. Experimental Section

    1.1 General methods and materials

    1.2 Detailed synthetic procedure and characterization data

    1.3 Proposed mechanism for the Scholl reaction

2. HR MALDI-TOF MS for **1**, **2** and **3**
3. Temperature-dependent ^{1}H NMR of **1**, **2** and **3**
4. X-ray crystallographic analysis of **1**, **2**, **3**, **8** and 1**3**
5. Fluorescence spectrum of **1**
6. Raman spectra of **1-3**
7. In situ spectroelectrochemistry
8. DFT calculation details
9. NMR spectra
10. High resolution mass spectra
11. References

1. Experimental section

1.1 General methods and materials

Nuclear magnetic resonance (NMR) spectra were recorded on a Bruker Avance III 500 spectrometer (500.13 MHz and 125.77 MHz for ^{1}H and ^{13}C respectively) and/or on a Bruker Avance III HD 300 spectrometer (300 MHz and 75.5 MHz for ^{1}H and ^{13}C respectively) using a 5 mm $^{1}H/^{13}C/^{19}F/^{31}P$ gradient probe. CD_2Cl_2 ($\delta(^{1}H) = 5.33$ ppm, $\delta(^{13}C) = 53.7$ ppm) or $C_2D_2Cl_4$ ($\delta(^{1}H) = 5.98$ ppm, $\delta(^{13}C) = 73.7$ ppm) were used as solvent, lock and internal standard. The 2D NMR spectra were recorded using the standard pulse sequences of the Bruker software package (TOPSPIN 3.2). Unless otherwise stated, the measurements were carried out at 30°C. The sample temperature was controlled by the Bruker variable temperature accessory BVT-3000. The mass spectrometry analysis was performed on a Bruker Autoflex Speed MALDI TOF MS (Bruker Daltonics, Bremen, Germany) using dithranol as matrix. High-Resolution Atmospheric Pressure Chemical Ionization (APCI) mass spectra was recorded with Agilent 6538 Ultra High Definition (UHD) Accurate-Mass Q-TOF LC/MC system, using the positive mode. UV-visible spectra were measured on an Agilent Cary 5000 UV-Vis-NIR spectrophotometer by using 10 mm optical-path quartz cell at room temperature. Photoluminescence spectra were measured on PerkinElmer fluorescence spectrometer LS 55. Raman spectra were recorded at ambient temperature using a Bruker Vertex 70 instrument equipped with a RAM II module (Nd-YAG laser, 1064 nm). Cyclic voltammetry measurements were carried out on a PARSTAT4000 potentiostat (Princeton Applied Research, Ametek) in a three-electrode cell. Electrolyte solutions were prepared by solving 0.1 M nBu$_4$NPF$_6$ in dichloromethane and the experiments were performed with a scan rate of 50 mVs^{-1} at room temperature. All measurements were recorded in nitrogen-purged solutions in anhydrous dichloromethane. A Pt-wire and a Pt-sheet were used as working and counter electrode, respectively. An AgCl-coated silver wire was used as pseudo reference electrode. All potentials are given against ferrocenium/ferrocene (Fc^{+}/Fc) redox couple. The spectroelectrochemical experiments were performed in the optical EPR cavity (ER 4104OR, Bruker Germany). EPR spectra were recorded by the EMX X-band CW spectrometer (Bruker, Germany). UV-Vis-NIR spectra were measured using the Avantes spectrometer AvaSpec-2048x14-USB2 with the CCD detector and AvaSpec-NIR256-2.2 with the InGaAs detector applying the AvaSoft 7.5 software (Avantes, The Netherlands). A light source Avantes

Avalight-DH-S-BAL was used. Both, the EPR spectrometer and the UV-Vis-NIR spectrometer are linked to a HEKA potentiostat PG 390 which triggers both spectrometers. Triggering was performed by the software package PotMaster v2x90 (HEKA Electronik, Germany). Each UV-Vis-NIR spectrum was collected relative to that of the neutral (non-charged) compound at the initial potential. For standard *in situ* EPR/UV-Vis-NIR spectroelectrochemical experiments an EPR flat cell was used. A laminated gold mesh as working electrode, an AgCl-coated silver wire as pseudo reference electrode, and a platinum wire as counter electrode were used in spectroelectrochemical experiments. (2-((Triisopropylsilyl) ethynyl)phenyl)boronic acid (**4**), [1] 1,4-bis(2-ethynylphenyl)buta-1,3-diyne (**10**) [2] and 2,3,4,5-tetrakis(4-(*tert*-butyl)phenyl)cyclopenta-2,4-dien-1-one (**11**) [3] were synthesized according to the literature.

1.2 Detailed synthetic procedure and characterization data

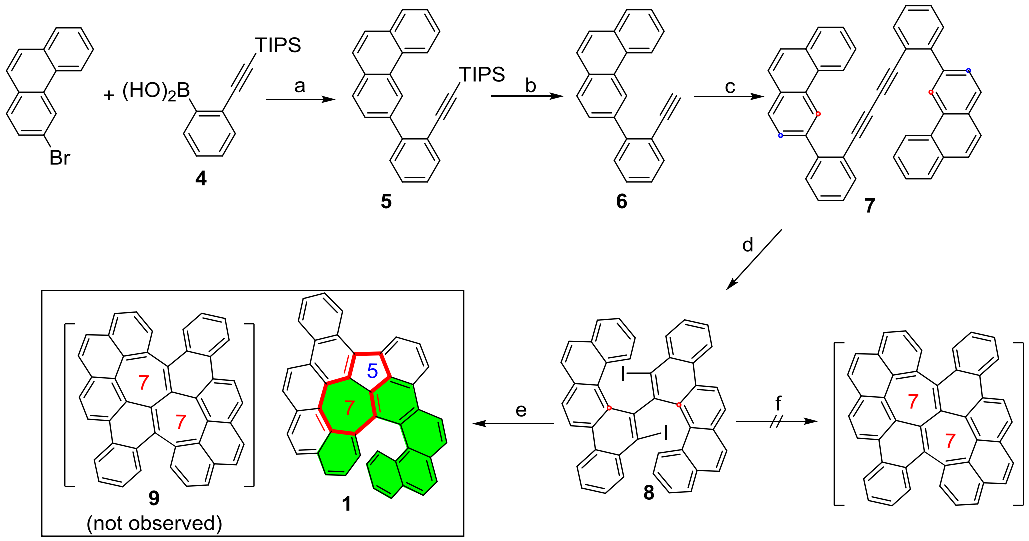

Scheme S1. Synthetic route to azulene-embedded helicene **1**. (a) $Pd(PPh_3)_4$, 2M K_2CO_3, THF, 90 °C, 20 h, 91%; (b) TBAF, THF, 1 h, quant.; (c) CuCl, piperidine, toluene, 60 °C, 3 h, 88%; (d) ICl, DCM, -78 °C, 2 h, 44%; (e) DDQ, CH_3SO_3H, DCM, 0 °C, 30 min, 83%; (f) $PdCl_2(PPh_3)_2$, Na_2CO_3, N,N-dimethylacetamide, 110 °C, 18 h or microwave (110 °C, 5 h), deiodination byproducts.

<u>Triisopropyl((2-(phenanthren-3-yl)phenyl)ethynyl)silane (5)</u>

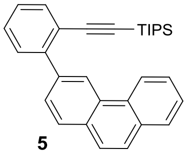

5

A mixture of 3-bromophenanthrene (500 mg, 1.94 mmol), (2-((triisopropylsilyl) ethynyl)phenyl)boronic acid (**4**) (882 mg, 2.92 mmol), $Pd(PPh_3)_4$ (112 mg, 0.097 mmol), 2 M K_2CO_3 aqueous solution (4 mL) and THF (20 mL) in a thick-walled Pyrex bottle was purged with Argon for 30 min. The sealed bottle was kept in an oil bath at 90 °C for 20 h. After being cooled to room temperature, the reaction mixture was removed under reduced pressure, and the residue was subjected to chromatography on silica gel (isohexane/CH_2Cl_2=6/1) to get **5** as yellow oil (769 mg, 91%).^{1}H NMR (300 MHz, CD_2Cl_2): 8.79 (s, 1H), 8.63 (d, 1H), 7.85-7.75 (m, 3H), 7.68 (s, 2H), 7.60-7.45 (m, 4H), 7.37 (t, 1H), 7.27 (t, 1H), 0.79 (s, 18H). ^{13}C NMR (75 MHz, CD_2Cl_2): 144.44, 138.89, 133.80, 132.18, 131.25, 130.38, 129.96, 129.79, 128.66, 128.47, 128.10, 128.05, 127.13, 126.96, 126.59, 126.52, 126.47, 123.50, 122.93, 122.23, 106.35, 94.17, 18.20, 11.19. HR-MS (APCI): m/z =434.2423, calcd. for: m/z = 434.2430, error = -1.56 ppm.

3-(2-Ethynylphenyl)phenanthrene (6)

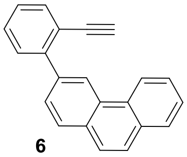

6

Argon was bubbled through a solution of **5** (200 mg, 0.46 mmol) in 10 mL THF for 10 min, and the 0.69 mL TBAF (1 M in THF) was added in the solution *via* dropwise. After 1h, the reaction was quenched with methanol. The solvent was removed under vacuum. The residue was then cleaned by flash column chromatography (isohexane/ CH_2Cl_2=4/1) to afford the crude product **6** (130 mg), which was directly used for the next step.

1,4-Bis(2-(phenanthren-3-yl)phenyl)buta-1,3-diyne (7)

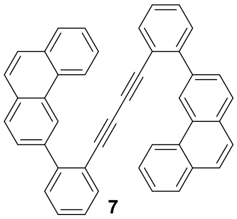

7

A mixture of compound **6** (130 mg, 0.47 mmol), CuCl (2.3 mg, 0.023 mmol), piperidine (7.95 mg) and toluene (2.5 mL) in an atmosphere of air was heated with stirring at 60 °C for 3h. After cooling to rt, the reaction mixture was poured in 15 mL methanol. The solution was then filtered to get **7** as a white solid (114 mg, 88%). ^{1}H NMR (300 MHz, CD_2Cl_2): 8.81 (s, 1H), 8.66 (d, 1H), 7.83 (d, 1H), 7.72-7.61 (m,

4H), 7.55-7.48 (m, 4H), 7.41 (t, 1H), 7.25 (t, 1H). ^{13}C NMR (75 MHz, CD_2Cl_2): 144.98, 137.97, 136.67, 134.32, 132.23, 131.36, 130.39, 129.93, 129.58, 128.57, 128.13, 127.59, 127.25, 126.73, 126.50, 123.24, 122.68, 120.19, 99.99, 81.79, 76.54. HR-MS (APCI): m/z =554.2020, calcd. for: m/z = 554.2034, error = -2.59 ppm.

(S)-8,8'-Diiodo-7,7'-bibenzo[*c*]chrysene (8)

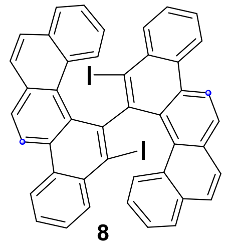

8

A solution of **7** (70 mg, 0.13 mmol) in anhydrous DCM (25 mL) was purged with argon for 30 minutes. After the solution was cooled to -78 °C, a 1 M solution of ICl in DCM (0.4 mL) was added dropwise under argon atmosphere. The reaction was stirred for 2 h and quenched with saturated sodium sulfite solution. The reaction mixture was diluted in 50 mL DCM and the organic layer was washed with a saturated sodium sulfite solution (3×10 mL) and dried over magnesium sulfate. The solvent was removed under reduced pressure. The crude product was purified by column chromatography (isohexane/ CH_2Cl_2=2/1) to get compound **8** as a light yellow solid (45 mg, 44%). ^{1}H NMR (300 MHz, CD_2Cl_2): 8.67 (d, 1H), 8.19 (d, 1H), 7.84 (d, 1H), 7.67 (t, 1H), 7.57 (t, 1H), 7.48 (d, 1H), 7.32 (d, 1H), 7.25 (dd, 2H), 7.16 (d, 1H), 6.64 (t, 1H), 6.10 (t, 1H). ^{13}C NMR (75 MHz, CD_2Cl_2): 145.61, 135.18, 131.79, 131.05, 130.61, 130.10, 129.81, 128.36, 127.50, 127.23, 127.07, 126.32, 125.85, 125.66, 124.97, 124.68, 122.77, 122.06, 121.77, 120.56. HR-MS (APCI): m/z =805.9954, calcd. for: m/z = 805.9967, error = -1.63 ppm.

Benzo[*pq*]benzo[3,4]fluoreno[2,1,9,8-*ghijk*]phenanthro[3,4-*m*]pleiadene (1)

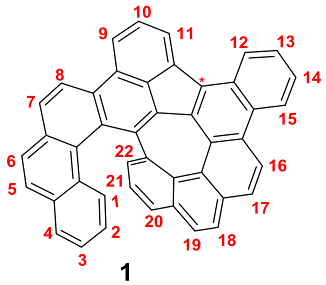

1

To a mixture of **8** (30 mg, 0.037 mmol) and DDQ (17 mg, 0.074 mmol) in dry CH_2Cl_2 (9.5 mL) at 0 °C, methanesulfonic acid (0.5 mL) was added by a syringe under argon atmosphere. After 30 minutes, the mixture was quenched by saturated $NaHCO_3$ solution (10 mL), and then extracted by CH_2Cl_2 twice. The combined extracts was washed with brine and dried over $MgSO_4$. After removal of solvent in vacuo, the crude material was purified by preparative TLC (isohexane/ CH_2Cl_2=2/1) to afford compound **1** (17 mg, 83%) as a red solid. ^{1}H NMR (500 MHz, $C_2D_2Cl_4$): 8.97 (d, 7.8 Hz, 1H; 12), 8.95 (d, 8.8 Hz, 1H; 16), 8.84 (d, 8.3 Hz, 1H; 8), 8.81 (d, 8.6 Hz, 1H; 15), 8.78 (d, 7.9 H, 1H; 11), 8.70 (d, 7.9 Hz, 1H; 9), 8.23 (d, 8.8 Hz, 1H; 17), 8.17 (d, 8.1 Hz, 1H; 1), 8.12 (d, 8.3 Hz, 1H; 7), 8.05 (t, 7.9 Hz, 1H; 10), 7.98 (d, 8.6 Hz, 1H; 18), 7.85 (d, 8.5 H, 1H; 6), 7.80 (t, 7.8 Hz, 1H; 13), 7.77 (d, 8.5 Hz, 1H; 5), 7.72 (t, 7.4 Hz, 1H; 14), 7.70 (d, 8.6 Hz, 1H; 19), 7.64 (d, 7.6 Hz, 1H; 4), 7.31 (d, 7.4 Hz, 1H; 20), 7.14 (t, 7.5 Hz, 1H; 3), 6.81 (d, 7.4 Hz, 1H; 22), 6.53 (t, 7.4 Hz, 1H; 21) and 6.41 ppm (t, 7.5 Hz, 1H; 2). ^{13}C NMR (125 MHz, $C_2D_2Cl_4$): 139.3, 137.9, 136.7, 136.6, 134.6, 133.15, 133.1, 132.9, 132.8 (*), 131.6, 131.35, 131.3, 131.0, 130.8 (22), 130.0, 129.6, 129.5, 129.3, 128.9 (20), 128.4 (10), 128.2, 128.0 (19), 127.9 (7), 127.8 (1), 127.7 (18), 127.6 (17), 127.5 (5), 127.4 (13), 126.5 (4), 126.3 (14), 126.2, 125.8 (6), 125.3 (3), 124.8 (15), 124.7 (2), 124.2 (12), 123.8 (21), 123.5 (11), 122.3 (16), 121.2 (9) and 121.1 ppm (8).

Comment: The assignment of proton signals within a spin system is based on their COSY correlation peaks ($^3J_{HH}$). The sequence of the spin systems is deduced from the ROESY spectrum that allows to identify neighboring protons of different spin systems. There are no ROESY correlations between ring systems containing protons H1-H4 and H20-H22. However, the neighborhood of H11 and H12 is proved by ROESY correlation. Finally, proton signals H11 and H12 show a correlation peak ($^3J_{CH}$) to the quarternary carbon at 132.8 ppm (marked with an asterix) confirming the assignment of these protons.

HR-MS (MALDI-TOF): m/z = 550.1714, calcd. for: m/z = 550.1722, error = - 1.4 ppm.

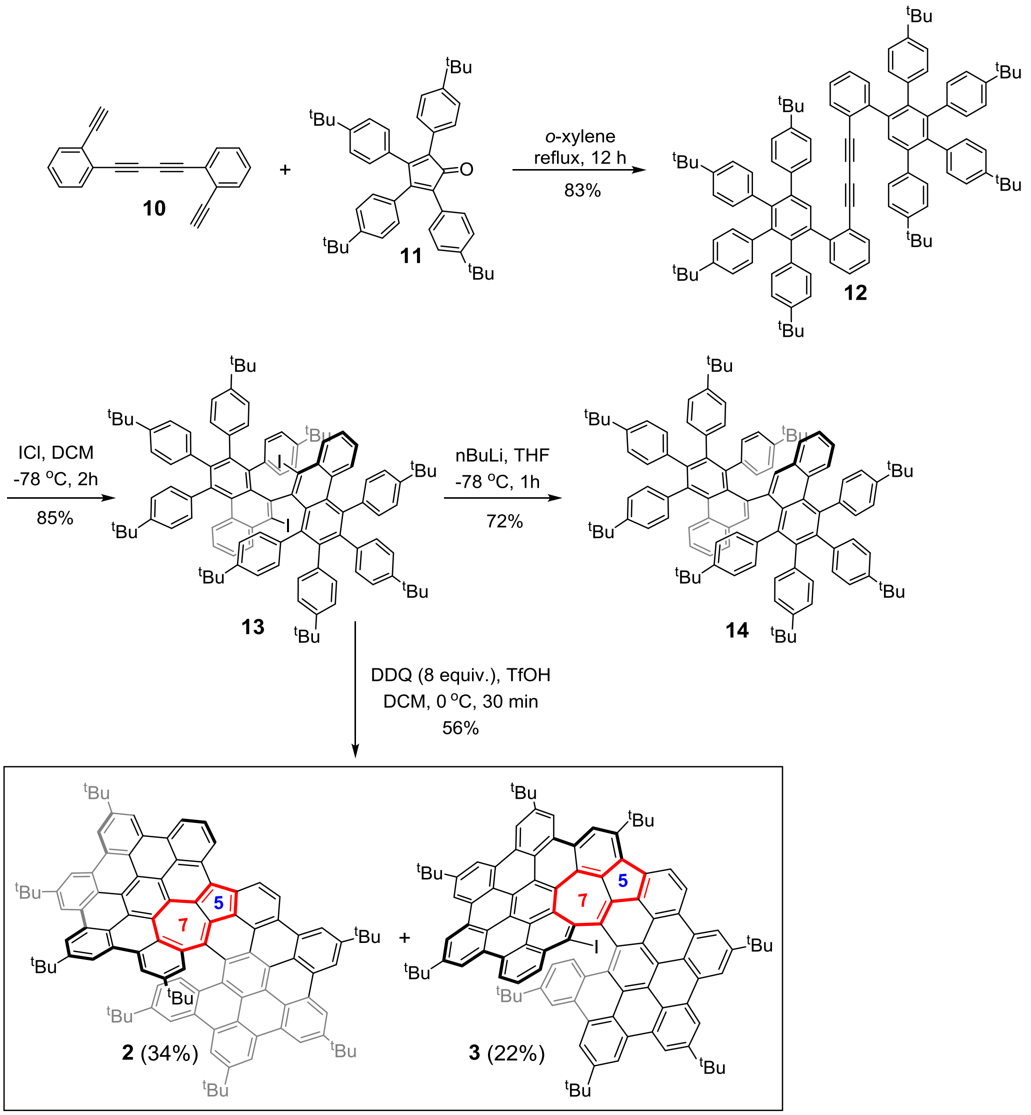

Scheme S2. Synthesis of π-extended azulene-embedded helical nanographenes **2** and **3**.

1,4-Bis(4''-(*tert*-butyl)-3',4',5'-tris(4-(*tert*-butyl)phenyl)-[1,1':2',1''-terphenyl]-2-yl)buta-1,3-diyne (12)

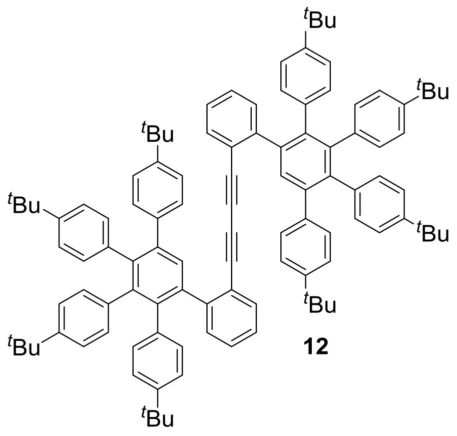

A solution of 320 mg (1.28 mmol) 1,4-bis(2-ethynylphenyl)buta-1,3-diyne (**10**) and 1.63 g 2,3,4,5-tetrakis(4-(tert-butyl)phenyl)cyclopenta-2,4-dien-1-one (**11**) (2.68 mmol) in 6 mL *o*-xylene under argon was heated to 165 °C for 12 hours. After cooling to room temperature, the reaction mixture was evaporated. The residue was then purified by column chromatography (silica gel, isohexane/CH_2Cl_2 = 2:1)) affording **12** as white solid (1.5 g, 83% yield). ^{1}H NMR (500 MHz, $C_2D_2Cl_4$, 60 °C): 7.57 (s, 2H), 7.51 (m, 2H), 7.14 (d, 8.4 Hz, 4H), 7.10 (d, 8.4 Hz, 4H), 7.15-7.05 (6H), 6.84 (d, 8.3 Hz, 4H), 6.82 (d, 8.3 Hz, 4H), 6.8-6.6 (16H), 1.22 (s, 18H; *t*Bu), 1.10 (s, 36H; *t*Bu) and 1.05 ppm (s, 18H; *t*Bu).^{13}C NMR (125 MHz, $C_2D_2Cl_4$, 60°C): 148.3, 147.7, 147.6, 147.2, 145.7, 142.0, 139.8, 139.6, 139.5, 138.8, 138.0, 137.2, 137.0, 136.8, 132.7 (CH), 131.0 (CH), 130.9 (CH), 130.6 (CH), 130.5 (CH), 129.5 (CH), 127.6 (CH), 125.8 (CH), 123.8 (CH), 122.9 (CH), 122.6 (CH), 121.6, 118.7, 82.0 (-C≡), 77.1 (-C≡), 33.9, 33.75, 33.7, 33.65 (all C of *t*Bu), 31.1, 31.0 and 30.95 ppm (all CH_3 of *t*Bu).

Comments: The ^{1}H NMR spectrum shows line broadening at 30°C. With increasing temperature the rate of the dynamic process increases and line narrowing is observed. Moreover, the signal splitting changes significantly in the temperature range studied. The ^{1}H and ^{13}C NMR measurements were carried out at 60°C. The aromatic CH carbons were identified based on a DEPT-135 spectrum.

HR-MS (APCI): m/z = 1410.8915, calcd. for: m/z = 1410.8920, error = -0.41 ppm.

5,5',6,6',7,7',8,8'-Octakis(4-(*tert*-butyl)phenyl)-10,10'-diiodo-9,9'-biphenanthrene (13)

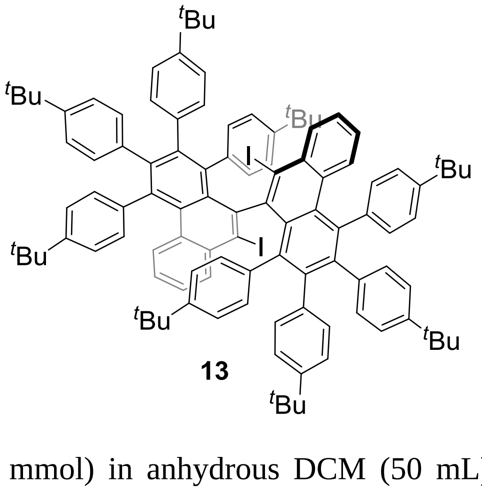

13

A solution of **12** (600 mg, 0.42 mmol) in anhydrous DCM (50 mL) was purged with argon for 30 minutes. After the solution was cooled to -78 °C, a 1 M solution of ICl in DCM (1.7 mL) was added dropwise under argon atmosphere. The reaction was stirred for 2 h and quenched with saturated sodium sulfite solution. The reaction mixture was diluted in 50 mL DCM and the organic layer was washed with

a saturated sodium sulfite solution (3×20 mL) and dried over magnesium sulfate. The solvent was removed under reduced pressure and the residue was purified by silica gel column chromatography by using isohexane/CH_2Cl_2 (2/1, v/v) as eluent to give compound **13** as a light yellow solid (600 mg, 85% yield). ^{1}H NMR (500 MHz, CD_2Cl_2): 8.09 (d, 8.2 Hz, 2H), 7.45 (d, 8.6 Hz, 2H), 7.24 (t, 8.4 Hz, 2H), 9.29 (s, 1H), 7.09 (d, 8.2 Hz, 2H), 7.05 (d, 8.2 Hz, 2H), 6.97 (d, 7.9 Hz, 2H), 6.92 (d, 8.1 Hz, 2H), 6.87 (d, 8.1 Hz, 2H), 6.85-6.75 (8H), 6.60-6.50 (8H), 6.38 (d, 8.1 Hz, 2H), 6.28 (d, 8.2 Hz, 2H), 6.22 (d, 8.4 Hz, 2H), 6.17 (d, 8.6 Hz, 2H), 1.26 (s, 18H; *t*Bu), 1.11 (s, 18H; *t*Bu), 1.06 (s, 18H; *t*Bu) and 0.87 ppm (s, 18H; *t*Bu). ^{13}C NMR (125 MHz, CD_2Cl_2): 149.2, 148.9, 147.8, 147.5, 147.2, 143.4, 141.5, 141.0, 138.8, 138.6, 138.4, 137.9, 134.1, 133.3, 132.3 (CH), 132.0 (CH), 131.95, 131.45 (CH), 131.4 (CH), 131.1 (CH), 130.9, 130.8 (CH), 130.75, 130.6 (CH), 130.1 (CH), 129.2 (CH), 126.6 (CH), 125.0 (CH), 124.5 (CH), 124.4 (CH), 123.2 (CH), 123.1 (CH), 122.8 (CH), 122.6 (CH), 122.3 (CH), 121.3, 121.1 (CH), 34.5, 34.2, 34.1, 33.9 (all C of *t*Bu), 31.4, 31.3, 31.2 and 31.0 ppm (all CH_3 of *t*Bu).

Comments: Despite it is possible to assign the ^{1}H NMR signals and most ^{13}C NMR signals to different aromatic rings, it is not possible to identify the position of these four rings at the central hexa-substituted moiety. Therefore, the assignments are not reported. The additional splitting of many ^{1}H NMR signals due to $^4J_{HH}$ coupling (1 – 2 Hz) is not outlined in the NMR data listing. The aromatic CH carbons were identified based on a DEPT-135 spectrum.

HR-MS (APCI): m/z =1662.6822, calcd. for: m/z = 1662.6853, error = -1.89 ppm.

5,5',6,6',7,7',8,8'-Octakis(4-(*tert*-butyl)phenyl)-9,9'-biphenanthrene (14)

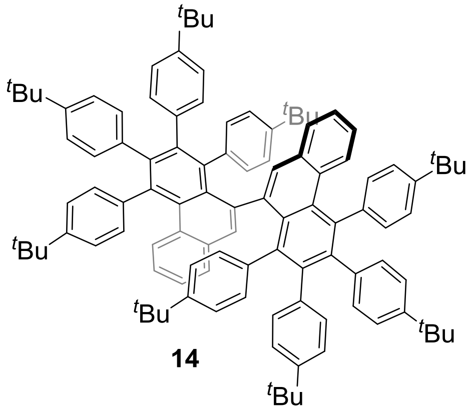

14

A solution of **13** (100 mg, 0.06 mmol) in anhydrous THF (10 mL) was degassed with argon for 30 minutes. After the solution was cooled to -78 °C, a 1.6 M solution of *n*-BuLi in hexane (0.11 mL, 3 equiv.) was added dropwise to the solution under argon atmosphere. The reaction was stirred for 1 h and

quenched with methanol (1 mL). The reaction mixture was diluted in 50 mL DCM and the organic layer was washed with water (3×20 mL) and dried over magnesium sulfate. After removal of the solvent under reduced pressure, the residue was purified by column flash chromatography on silica (isohexane/DCM; 2/1) to obtain the title compound **14** (61 mg, 72%) as a yellow solid. ^{1}H NMR (500 MHz, CD_2Cl_2): 7.58 (d, 7.5 Hz, 2H), 7.48 (s, 2H), 7.25-7.20 (4H), 7.20-7.10 (6H), 7.05-6.95 (4H), 6.93 (d, 8.0 Hz, 2H), 6.84 (d, 8.1 Hz, 2H), 6.82-6.75 (4H), 6.65 (d, 8.2 Hz, 2H), 6.63-6.58 (6H), 6.41 (d, 8.1 Hz, 2H), 6.32 (d, 8.2 Hz, 2H), 5.99 (d, 8.2 Hz, 2H), 5.67 (d, 8.2 Hz, 2H), 1.29 (s, 18H; *t*Bu), 1.17 (s, 18H; *t*Bu), 1.14 (s, 18H; *t*Bu) and 0.85 ppm (s, 18H; *t*Bu). ^{13}C NMR (125 MHz, CD_2Cl_2): 149.3, 147.8, 147.75, 147.1, 141.1, 140.6, 140.5, 139.3, 139.0, 138.2, 138.1, 137.7, 133.9 (CH), 133.7 (CH), 133.4, 132.0 (CH), 131.9 (CH), 131.8 (CH), 131.2 (CH), 131.1 (CH), 131.0, 130.5, 130.2 (CH), 129.8 (CH), 128.7 (CH), 127.6 (CH), 125.7, 125.4 (CH), 125.1 (CH), 124.5 (CH), 123.8 (CH), 123.2 CH), 123.1 (CH), 123.0 (CH), 122.9 (CH), 122.4 (CH), 121.7 (CH), 34.5, 34.3, 33.9 (all C of *t*Bu), 31.4, 31.35, 31.3 and 30.9 ppm (all CH_3 of *t*Bu).

Comments: Despite it is possible to assign the ^{1}H NMR signals and most ^{13}C NMR signals to different aromatic rings, it is not possible to identify the position of these four rings at the central hexa-substituted moiety. Therefore, the assignments are not reported. The additional splitting of many ^{1}H NMR signals due to $^{4}J_{HH}$ coupling (1 – 2 Hz) is not outlined in the NMR data listing. The aromatic CH carbons were identified based on a DEPT-135 spectrum.

HR-MS (MALDI-TOF): m/z = 1410.8915, calcd. for: m/z = 1410.8921, error = -0.43 ppm.

Azulene-embedded helical nanographenes (2 and 3)

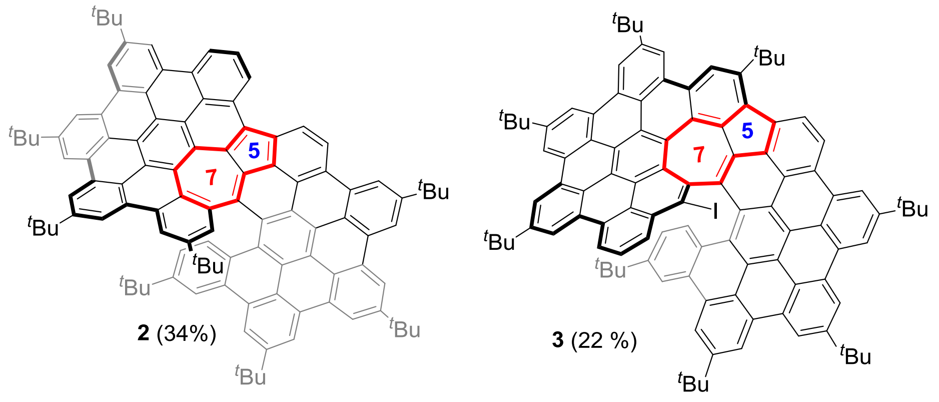

2 (34%) **3** (22 %)

13 (30 mg, 0.018 mmol) and DDQ (32.7 mg,0.144 mmol) were dissolved in 9.5 mL of anhydrous

CH_2Cl_2 in a 25 mL Schlenk flask equipped with a magnetic stirrer under argon. The solution was cooled down to 0 °C with ice bath. After stirring for 10 min, 0.5 mL of trifluoromethanesulfonic acid (TfOH) was added dropwise through a syringe. The reaction mixture was kept stirred at 0 ºC for 30 min and then quenched with 1 mL Et_3N. The reaction mixture was diluted with 20 mL DCM and the organic layer was washed with water, dried with anhydrous magnesium sulfate and concentrated under reduced pressure. The residue was then purified by preparative silica TLC plate (eluent: isohexane/ CH_2Cl_2 (3/1)) to afford the title compound **2** (10 mg, 34 %) and compound **3** (6.5 mg, 22%) as red solid, respectively.

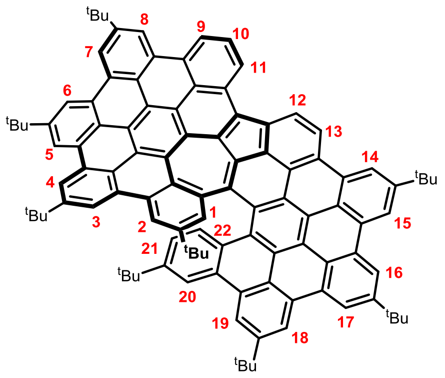

2

^{1}H NMR (500 MHz, CD_2Cl_2): 9.35 (s, 1H; 16), 9.34 (s, 1H; 17), 9.31 (s, 1H; 18), 9.29 (s, 1H; 5), 9.27 (s, 1H; 15), 9.26 (s, 1H; 6), 9.21 (s, 1H; 4), 9.16 (s, 1H; 7), 9.08 (s, 1H; 19), 9.025 (s, 1H; 14), 9.02 (d, 7.5 Hz, 1H; 11), 8.97 (d, 8.2 Hz, 1H; 13), 8.97 (s, 1H; 8), 8.93 (d, 8.2 Hz, 1H; 12), 8.93 (s, 1H; 3), 8.78 (d, 7.5 Hz, 1H; 9), 8.65 (d, 8.5 Hz, 1H; 22), 8.63 (s, 1H; 20), 8.62 (s, 1H; 2), 7.86 (t, 7.5 Hz, 1H; 10), 7.30 (s, 1H; 1), 6.56 (d, 8.5 Hz, 1H; 21), 1.91 (s, 9H, $t\mathrm{Bu}_{5/6}$), 1.88 (s, 9H, $t\mathrm{Bu}_{16/17}$), 1.86 (s, 9H, $t\mathrm{Bu}_{3/4}$), 1.85 (s, 9H, $t\mathrm{Bu}_{18/19}$), 1.72 (s, 9H, $t\mathrm{Bu}_{7/8}$), 1.63 (s, 9H, $t\mathrm{Bu}_{14/15}$), 1.28 (s, 9H, $t\mathrm{Bu}_{20/21}$) and 0.85 ppm (s, 9H, $t\mathrm{Bu}_{1/2}$).

^{13}C NMR (125 MHz, $C_2D_2Cl_4$): 149.9, 149.7, 149.6, 149.5, 149.4, 147.3, 141.8, 139.1, 137.9, 134.0, 133.7, 133.6, 132.1, 131.9 (CH), 131.1, 130.9, 130.6, 130.4, 130.2, 130.0, 12.9, 129.8 (CH), 129.5, 129.3, 129.0, 128.7, 127.8, 127.1 (CH), 126.9, 126.2, 124.4, 124.1, 124.0, 123.8, 123.2 (2x CH), 123.1, 123.0, 122.8, 122.7, 122.5 (CH), 122.4, 121.8, 121.2 (CH), 120.8, 120.4 (CH), 120.2, 120.1 (CH), 120.0 (CH), 119.6 (CH), 119.3 (CH), 119.1 (3x CH), 118.9 (3x CH), 118.7 (CH), 118.6 (CH), 118.5 (CH), 118.4 (CH), 35.6 , 35.55, 35.5, 35.45 (2x C), 35.4, 34.5, 33.7 (all C of *t*Bu), 31.85, 31.8, 31.75, 31.7, 31.65, 31.4 and 30.2 ppm (all CH_3 of *t*Bu).

Comments: The assignment of proton signals within a spin system is based on their TOCSY correlation peaks and multiplicities. The two singlets in 1,3-position of seven rings show long-range coupling resulting in intense TOCSY correlations. The sequence of the spin systems is deduced from the ROESY spectrum that allows to identify neighboring protons of different spin systems. It should be mentioned that the ^{1}H NMR spectrum of **2** shows a significant concentration-dependence. It is necessary to carefully adjust the concentration to obtain a line separation (Fig. S41) that allows to observe the TOCSY and ROESY correlations shown in Figures S42 and S413.

HR-MS (MALDI-TOF): m/z = 1390.7355, calcd. for: m/z = 1390.7356, error = - 0.07 ppm.

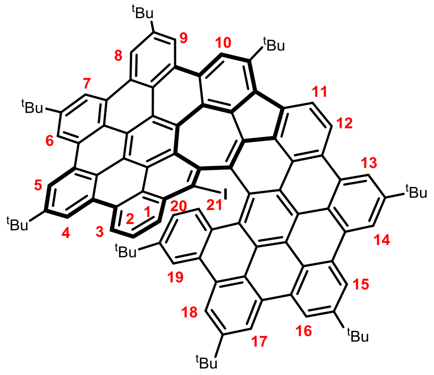

3

^{1}H NMR (500 MHz, $C_2D_2Cl_4$): 9.38 (s, 1H; 13), 9.37 (d, 8.5 Hz, 1H; 17), 9.35 (s, 1H; 14), 9.30 (s, 1H; 6), 9.29 (s, 1H; 15), 9.28 (s, 1H; 5), 9.22 (s, 1H; 7), 9.19 (s, 1H; 16), 9.18 (s, 1H; 10), 9.16 (s, 1H; 8), 9.14 (d, 8.5 Hz, 1H; 11), 9.13 (s, 1H; 17), 9.11 (s, 1H; 9), 9.07 (s, 1H; 4), 8.83 (d, 8.1 Hz, 1H; 3), 8.61 (s, 1H; 18), 8.32 (d, 8.4 Hz, 1H; 21), 7.91 (d, 8.1 Hz, 1H; 1), 7.90 (s, 1H; 19), 7.68 (t, 8.1 Hz, 1H; 2), 5.49 (d, 8.4 Hz, 1H; 20), 2.18 (s, 9H, $t\mathrm{Bu}_{10}$), 1.86 (s, 9H, $t\mathrm{Bu}_{6/7}$), 1.85 (s, 9H, $t\mathrm{Bu}_{13/14}$), 1.83 (s, 9H, $t\mathrm{Bu}_{4/5}$), 1.81 (s, 9H, $t\mathrm{Bu}_{8/9}$), 1.80 (s, 9H, $t\mathrm{Bu}_{15/16}$), 1.72 (s, 9H, $t\mathrm{Bu}_{17/18}$) and 0.41 ppm (s, 9H, $t\mathrm{Bu}_{19/20}$).

^{13}C NMR (125 MHz, $C_2D_2Cl_4$): 150.0, 149.9, 149.6, 149.5, 149.0, 148.7, 146.7, 141.6, 139.2, 138.6, 138.5, 137.9, 133.4, 133.3, 132.4, 131.3, 130.9, 130.7, 130.6, 130.5, 130.4, 130.3, 130.2 (21), 130.1 (1), 129.7, 129.4, 129.3, 129.2, 129.1, 128.7, 127.3, 127.2 (11), 126.7 (2), 126.6, 125.7, 125.1, 124.4, 123.6, 135.5, 123.3, 123.0, 122.8, 122.7, 122.6, 122.3 (2), 122.0, 121.8 (3), 121.6 (10), 121.4 (12), 120.7, 119.7 (13), 119.5 (8), 119.4 (14), 119.3 (19), 119.0 (16), 118.8 (5, 7, 15), 118.6 (4, 17), 118.5 (18), 118.4 (9), 109.3 (C-I), 35.8, 35.5, 35.4, 35.3, 33.4 (all C of *t*Bu), 31.8, 31.7, 30.4 and 30.2 ppm (all CH_3 of *t*Bu).

Comments: The assignment of proton signals (Fig. S47) within a spin system is based on their TOCSY

correlation peaks (Fig. S48) and multiplicities. The two singlets in 1,3-position of seven rings show long-range coupling resulting in intense TOCSY correlations. The sequence of the spin systems is deduced from the ROESY spectrum (Fig. S49) that allows to identify neighboring protons of different spin systems. The tert. butyl group next to H_{10} shows a ROESY correlation to the doublet of H_{11}.

HR-MS (MALDI-TOF): m/z = 1516.6312, calcd. for: m/z = 1516.6322, error = - 0.66 ppm.

1.3 Proposed mechanism for the Scholl reaction

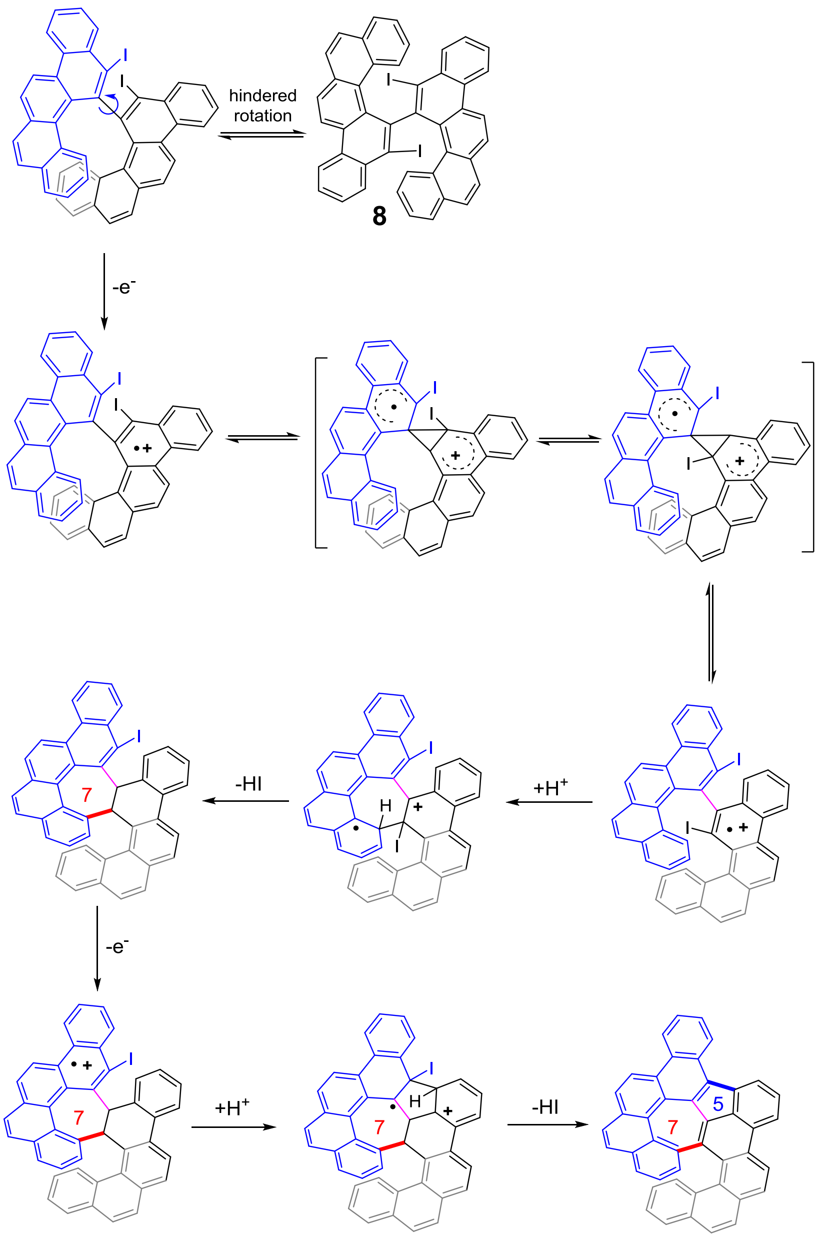

Scheme S3. Possible pathways to formation of azulene-embedded helicene **1** from precursor **8**.

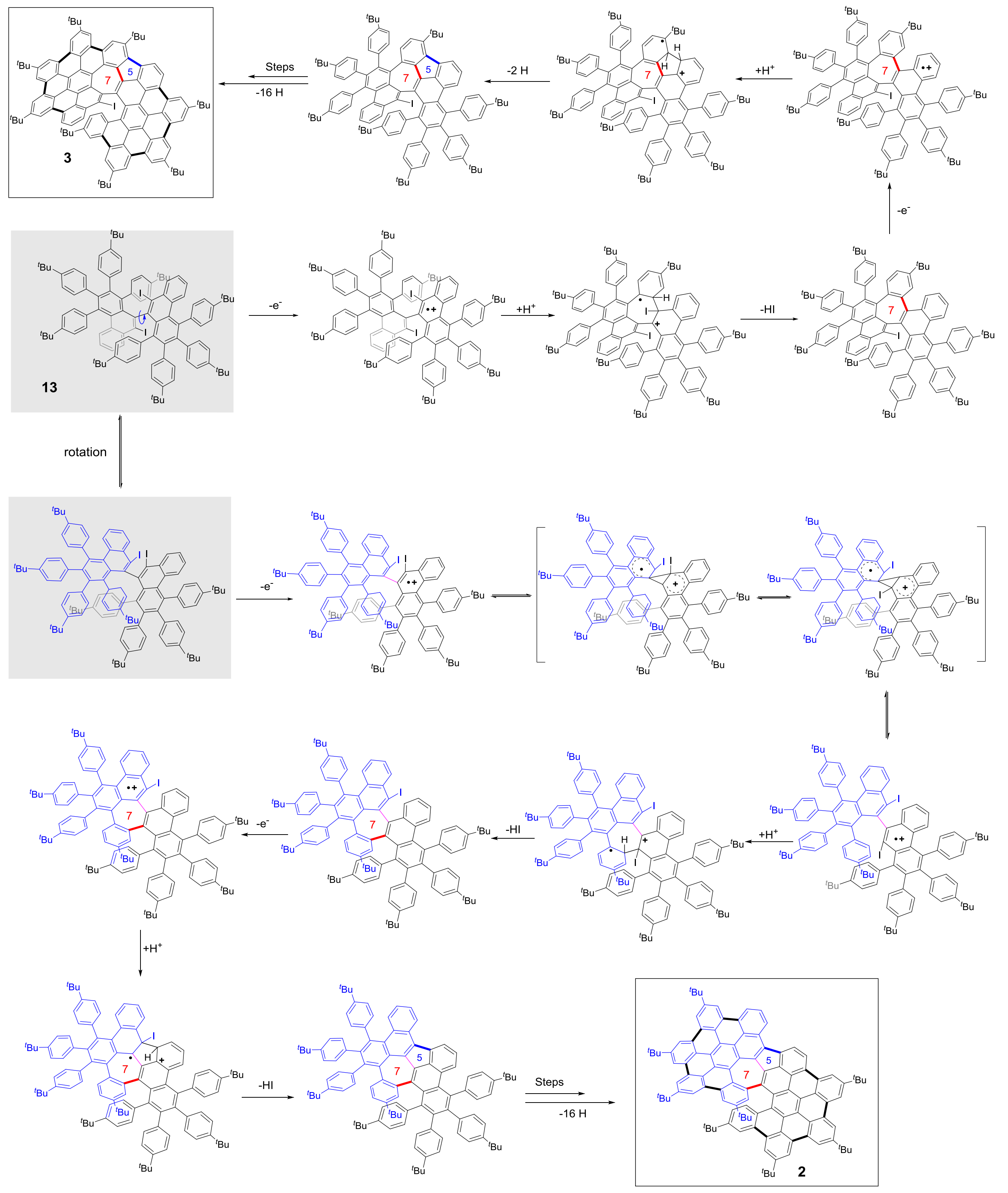

Scheme S4. Possible pathways to formation of **2** and **3** containing a pentagon-heptagon pair from precursor **13**.

2. HR MALDI-TOF MS for 1, 2 and 3.

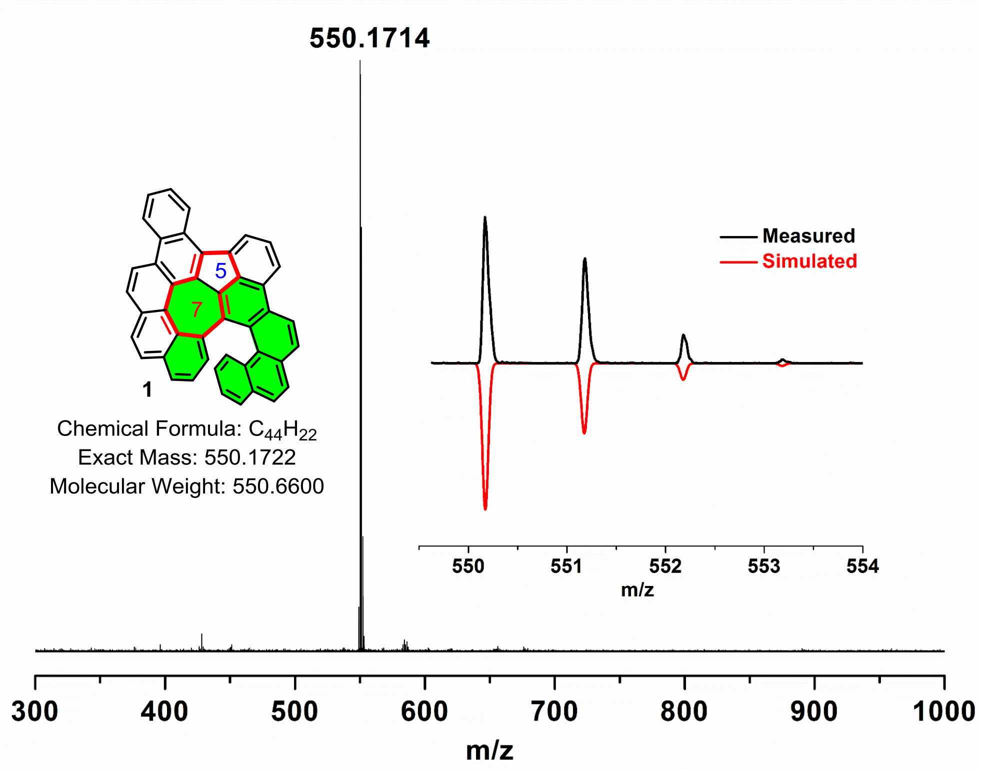

Figure S1. HR-MALDI-TOF mass spectrum of **1**.

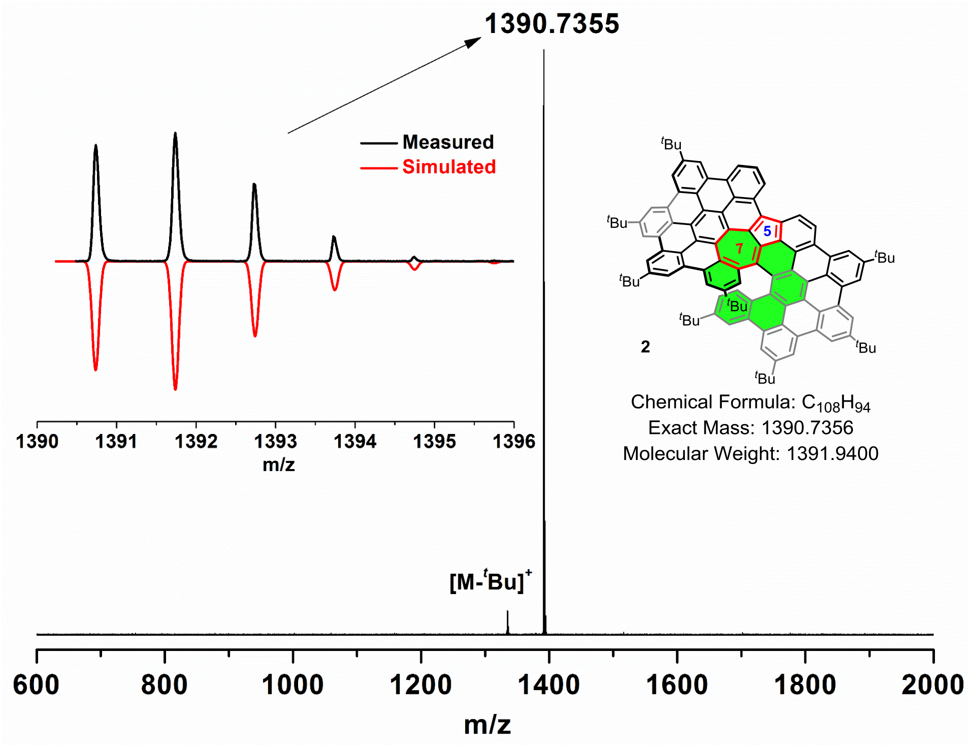

Figure S2. HR-MALDI-TOF mass spectrum of **2**.

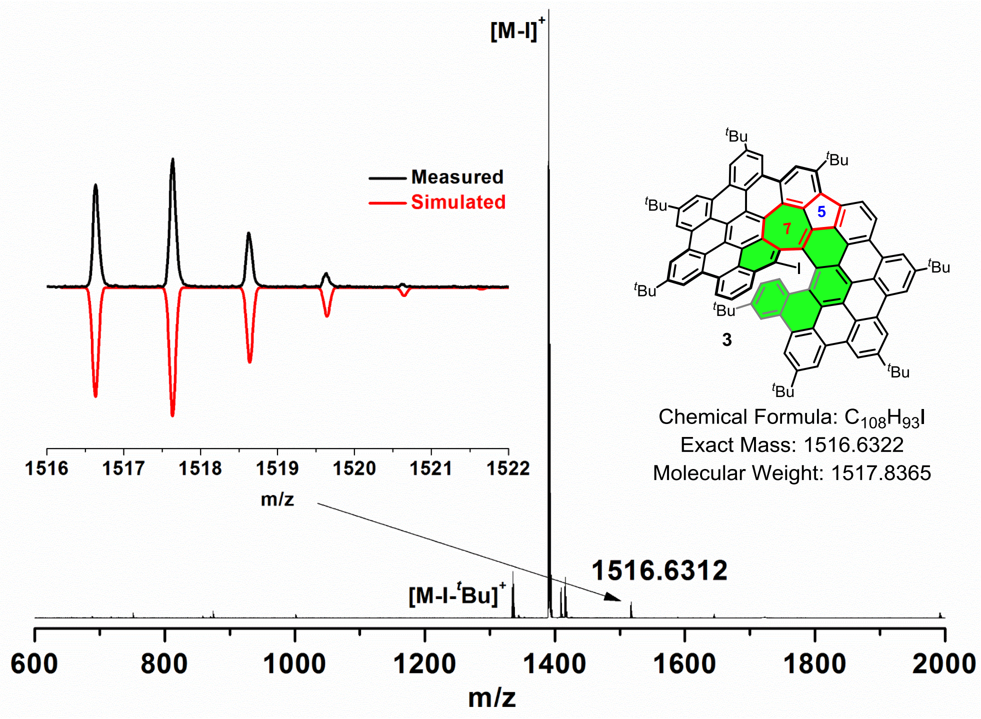

Figure S3. HR-MALDI-TOF mass spectrum of **3**.

3. Temperature-dependent ^{1}H NMR of 1, 2 and 3

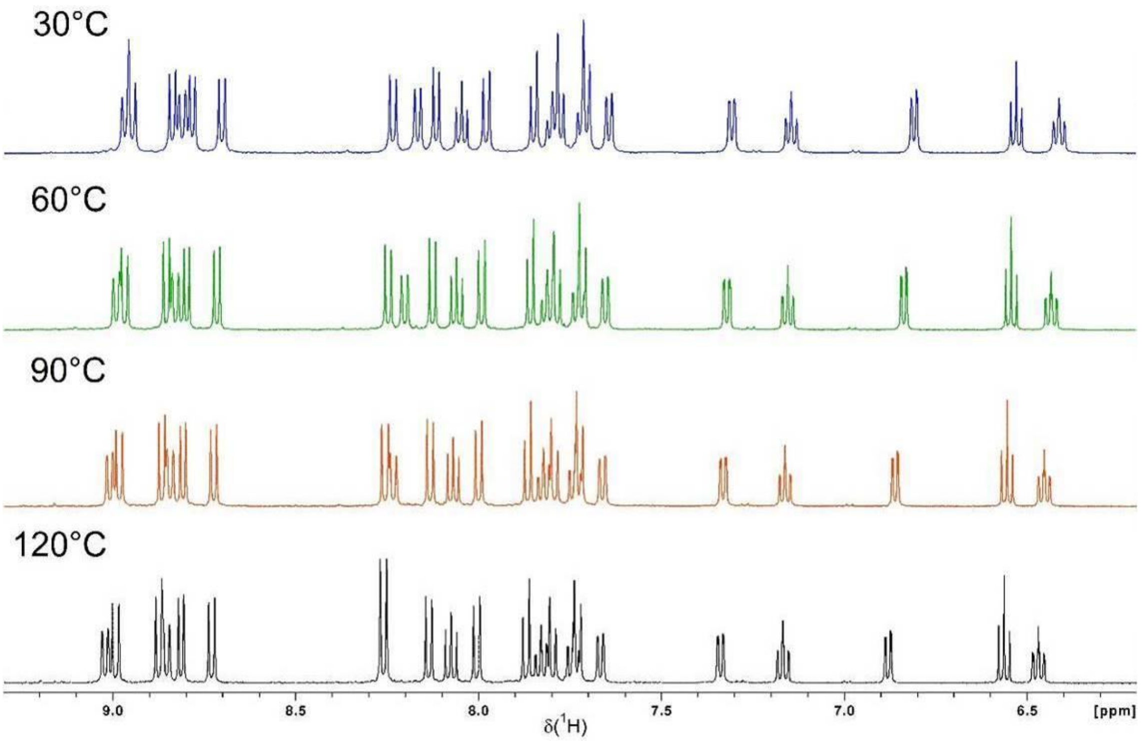

Figure S4. VT ^{1}H NMR spectra of **1** (500 MHz, $C_2D_2Cl_4$).

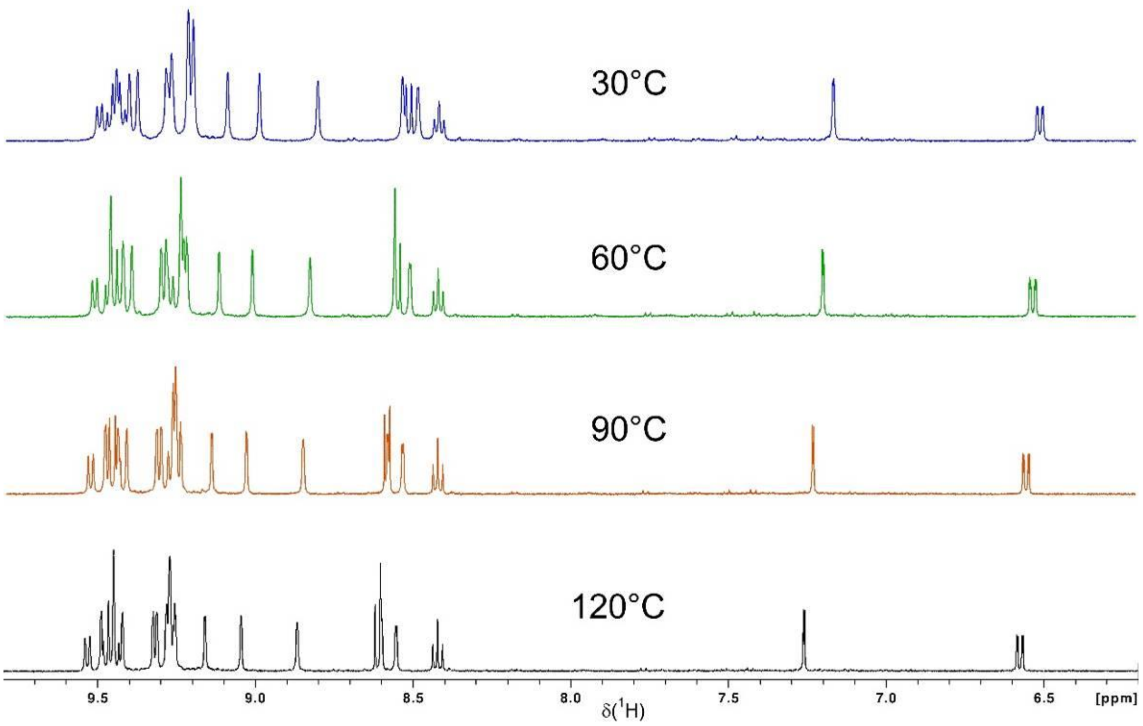

Figure S5. VT ^{1}H NMR spectra of **2** (500 MHz, $C_2D_2Cl_4$).

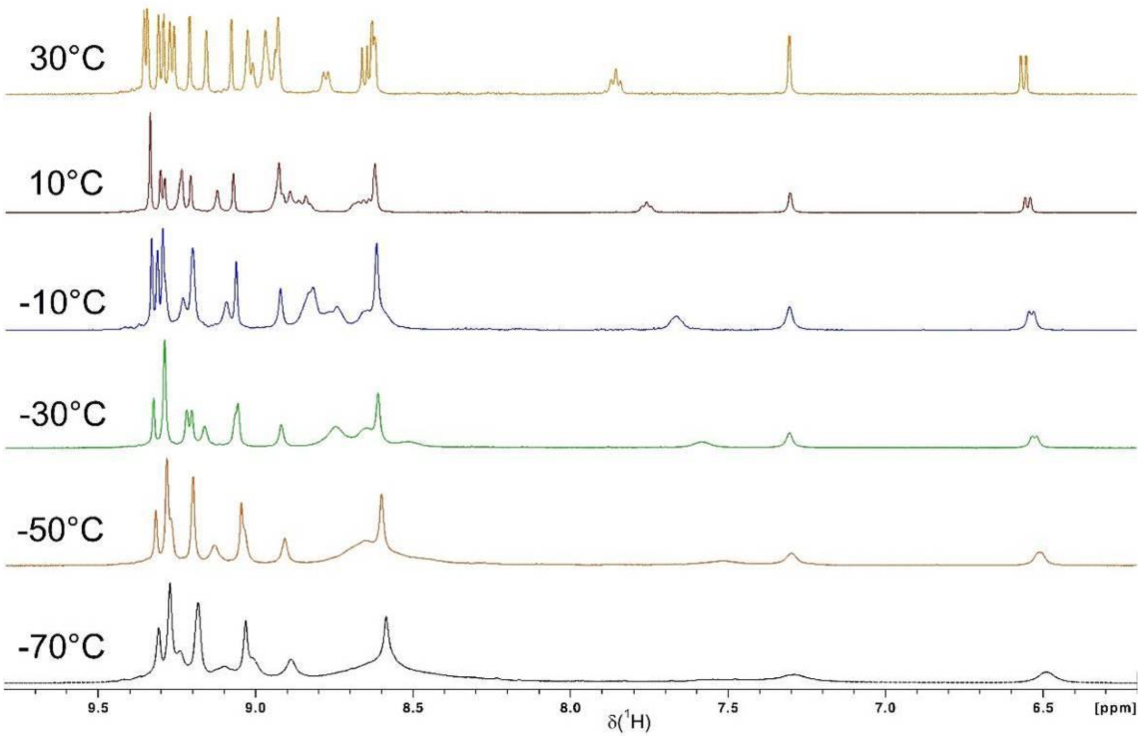

Figure S6. VT ^{1}H NMR spectra of **2** (500 MHz, CD_2Cl_2).

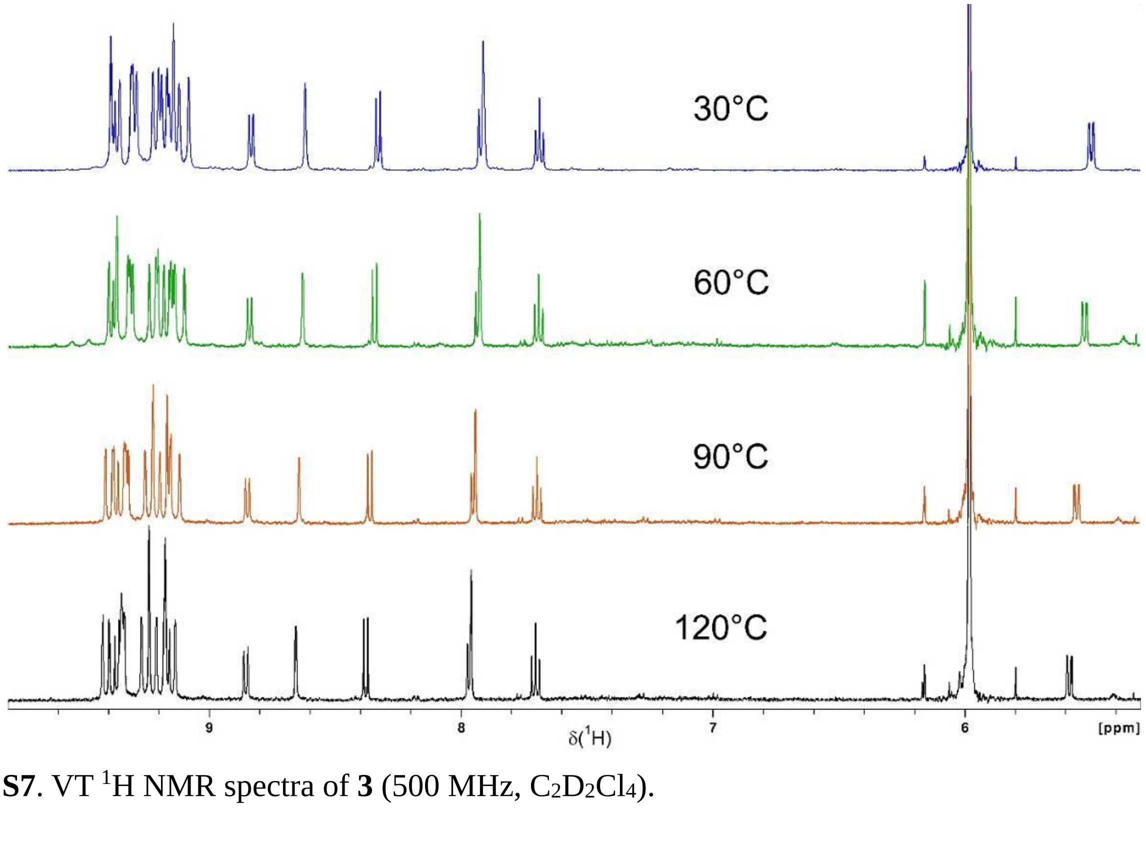

Figure S7. VT ^{1}H NMR spectra of **3** (500 MHz, $C_2D_2Cl_4$).

4. X-ray crystallographic analysis of 1, 2, 3, 8 and 13.

Single crystals of **8** were obtained by slow diffusion of n-hexane into a CH_2Cl_2 solution, while those of **3** and **13** were obtained by slow diffusion of acetonitrile into a CH_2Cl_2 solution, respectively. Single crystals of **1** and **2** were grown by slowly diffusing methanol vapor into its $C_2H_4Cl_2$ solution, respectively. The X-ray crystallographic coordinates for structures reported in this article have been deposited at the Cambridge Crystallographic Data Centre (CCDC), under deposition number, CCDC 1966144 (for **1**) 1949090 (for **2**), 1946579 (for **3**), 1963531 (for **8**) and 1949091 (for **13**). These data can be obtained free of charge from CCDC via http://www.ccdc.cam.ac.uk/data_request/cif

Table **S1**. Summary of crystal data and reflection collection parameters for compounds **1**, **2**, **3**, **8** and **13**.

	**1**	**2**	**3**
Moiety formula	$C_{44}H_{22}$	$C_{108}H_{94}$	$C_{110.78}H_{102.78}Cl_{1.35}IO_{2.11}$
Formula weight	550.61	1391.83	1642.36
Crystal size, mm	0.05 × 0.08 × 0.2	0.1 × 0.08 × 0.06	0.133 × 0.038 × 0.032
Crystal system	monoclinic	triclinic	triclinic
space group	*C*2/*c*	*P*-1	*P*-1
a, Å	31.020(6)	12.870(3)	16.8195(3)
b, Å	12.030(2)	14.460(3)	18.9974(3)
c, Å	17.400(4)	21.500(4)	28.7509(4)
α, deg	90	86.13(3)	80.5870(10)
β, deg	122.32(3)	80.55(3)	80.5440(10)
γ, deg	90	80.53(3)	87.5060(10)
V, Å^3	5487(2)	3889.7(14)	8938.5 (2)
Z	8	2	4
$D_{calcd.}$, g cm^{-3}	1.333	1.188	1.220
F_{000}	2288	1484	3441.0
Temperature, K	190 (2)	200(2)	100 (10)
Radiation	synchrotron (0.82656)	synchrotron (λ = 0.895)	CuKα (λ = 1.54184)
μ, mm^{-1}	0.105	0.113	3.569
2Θ range for data collection/°	4.4 to 64.1	3.6 to 66.8	4.716 to 153.572
Index ranges	$-38 \leq h \leq 39$, $-13 \leq k \leq 13$, $-22 \leq l \leq 21$	$-15 \leq h \leq 15$, $-16 \leq k \leq 16$, $-25 \leq l \leq 25$	$- 20 \leq h \leq 21$, $- 23 \leq k \leq 23$, $- 35 \leq l \leq 36$
no. of collected reflections	34020	42499	96153
no. of unique ref.(R_{int})	3722 (0.0335)	8152 (0.0485)	37180 (0.0419)
Data/restraints/parameters	3722 / 0 / 398	8152 / 72 / 1059	37180 / 311 / 2168
R_1, wR_2 [obs I>2σ (I)]	0.0663, 0.1852	0.0566, 0.1492	0.0554, 0.1530
R_1, wR_2 (all data)	0.0933, 0.2109	0.0804, 0.1680	0.0666, 0.1625
residual peak/hole, e. Å^{-3}	0.384 / -0.183	0.349/ -0.236	1.41 /-1.56
Goodness-of-fit on F^2	1.039	1.010	1.029

	**8**	**13**
Moiety formula	$C_{89}H_{50}Cl_2I_4$	$C_{108}H_{112}I_2$
Formula weight	1697.79	1663.77
Crystal size, mm	0.241 × 0.131 × 0.051	0.2 × 0.2 × 0.1
Crystal system	triclinic	monoclinic
space group	*P*-1	$P2_1/n$
a, Å	9.41848(16)	18.780(4)
b, Å	18.5089(3)	20.500(4)
c, Å	19.6834(3)	23.550(5)
α, deg	109.9677(15)	90
β, deg	92.2630(13)	93.11(3)
γ, deg	97.7378(13)	90
V, Å^3	3182.19(9)	9053(3)
Z	2	4
$D_{calcd.}$, g cm^{-3}	1.772	1.221
F_{000}	1660.0	3464
Temperature, K	100.01 (10)	190 (2)
Radiation	CuKα (λ = 1.54184)	synchrotron (λ = 0.82656)
μ, mm^{-1}	16.549	1.106
2Θ range for data collection/°	4.796 to 153.524	3.9 to 73.4
Index ranges	$-10 \le h \le 11$, $-23 \le k \le 19$, $-24 \le l \le 24$	$-27 \le h \le 26$, $-27 \le k \le 27$, $-32 \le l \le 32$
no. of collected reflections	33828	144122
no. of unique ref.(R_{int})	13254 (0.0219)	25199 (0.0314)
Data/restraints/parameters	13254 / 60 / 884	25199 / 36 / 1046
R_1, wR_2 [obs I>2σ (I)]	0.0275, 0.0718	0.0455, 0.1204
R_1, wR_2 (all data)	0.0278, 0.0720	0.0517, 0.1247
residual peak/hole, e. Å^{-3}	159/ -1.36	1.911/ -1.397
Goodness-of-fit on F^2	1.064	1.052

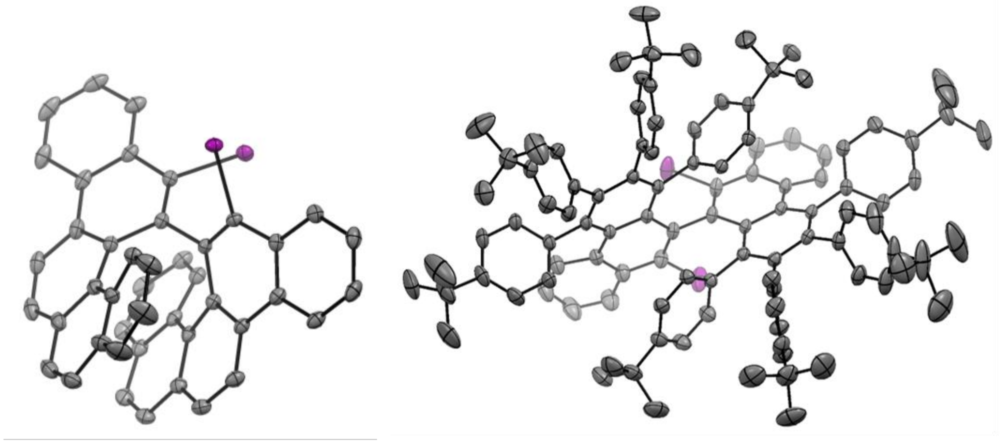

Figure S8. Single-crystal structure of compound **8** (left) and **13** (right).

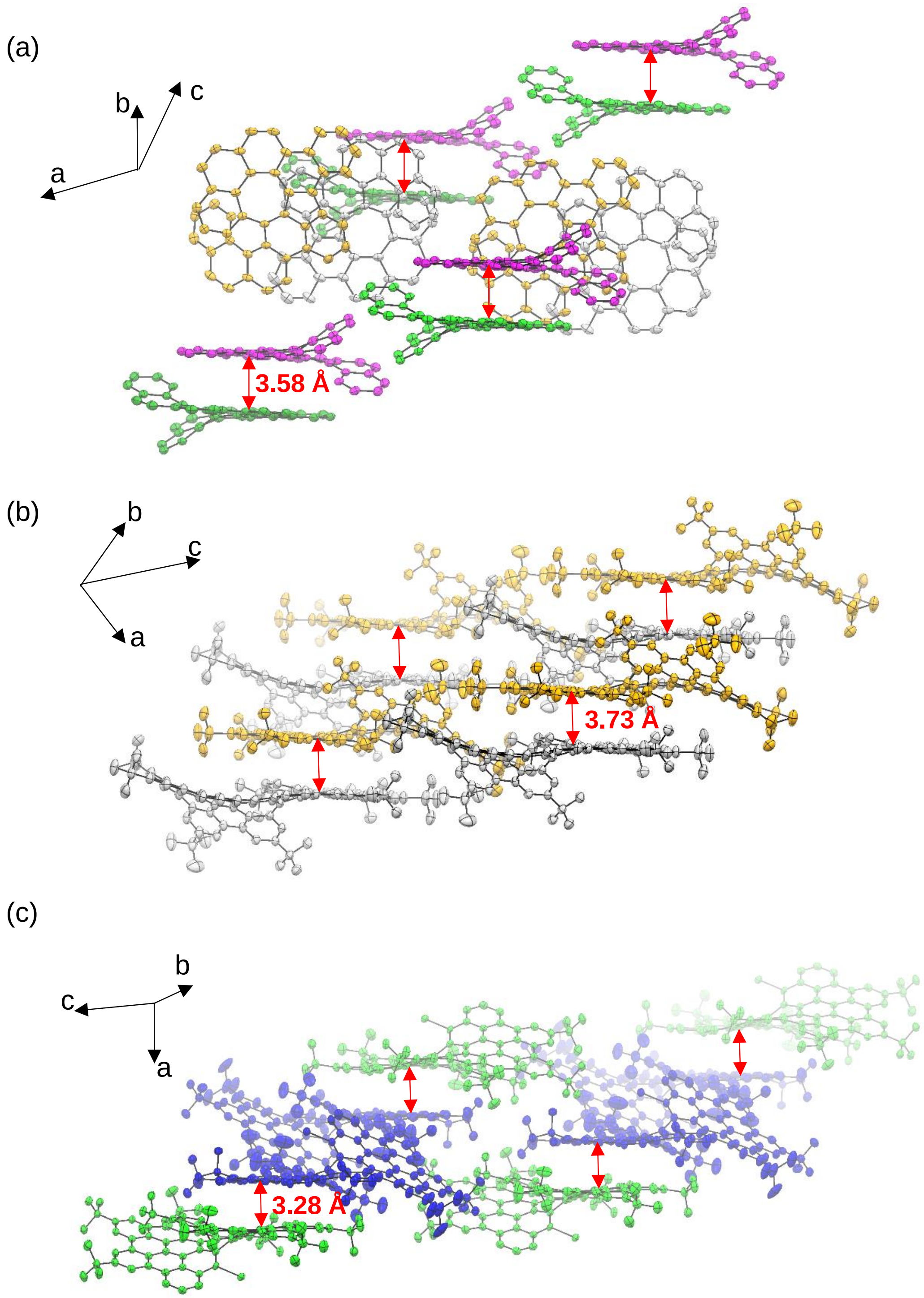

Figure S9. Molecular packing of (a) **1**, (b) **2** and (c) **3** in their crystals. Two enantiomers are shown in different colors, and all hydrogen atoms and solvent molecules are hided for clarity. For **1**, yellow and green represent the (*P*)-isomer, gray and pink represent the (*M*)-isomer. For **2**, grey and yellow represent the (*P*) and (*M*)-isomers, respectively. For **3**, green and blue represent the (*P*) and (*M*)-isomers, respectively.

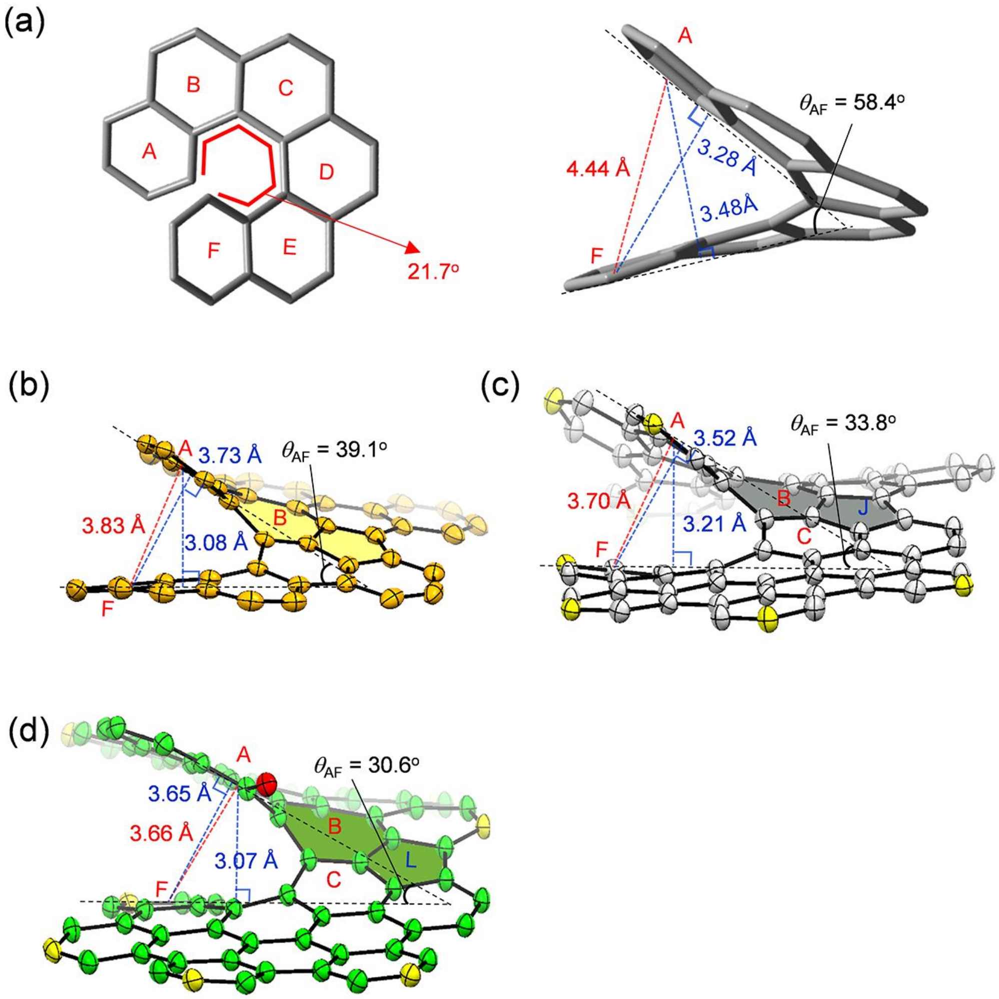

Figure S10. Front view and side view of the crystal structure of (a) [6]helicene (CCDC 1175421). Side view of the crystal structures of (b) **1**, (c) **2** and (d) **3**. The vertical distance *d* from the centroid of the ring A to the plane defined by the ring F are 3.21 Å for **2** and 3.07 Å for **3**, and that from the centroid F to the plane A are 3.52 Å for **2** and 3.65 Å for **3**, respectively. Consequently, the mean vertical distance between the terminal rings A and F of **2** and **3** are comparable (3.36 Å for both molecules), which are slightly smaller than that of in **1** (3.41 Å) and the pristine [6]helicene (3.38 Å), indicating the strong intramolecular π-π interaction for the large π-extended systems **2** and **3**. The angle between the two planes (θ_{AF}) of the terminal rings A and F in **1** and the pristine [6]helicene is 39.1° and 58.4°, respectively. The mean value of the four torsion angles in **1** and the pristine [6]helicene is measured to be 24.8° and 21.7°, respectively.

5. Fluorescence spectrum of 1

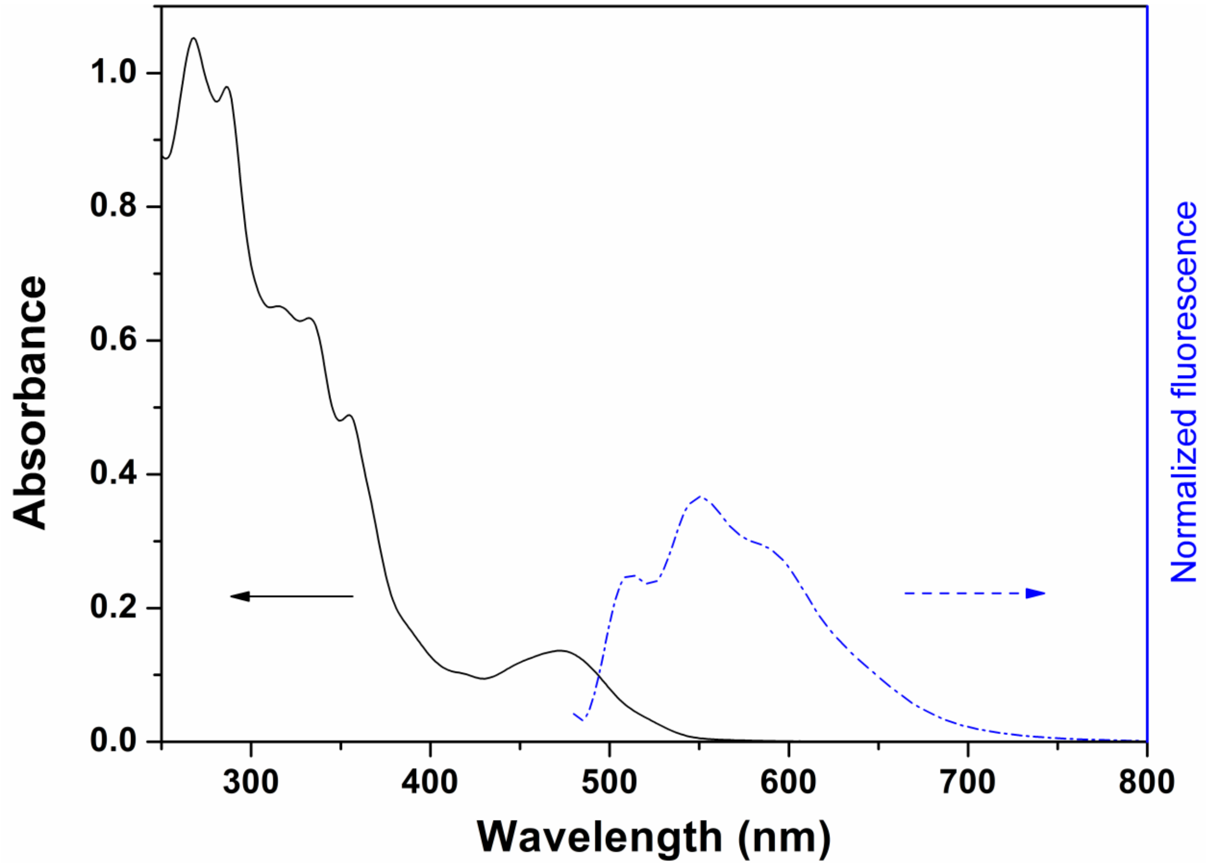

Figure S11. Absorption and photoluminescence spectra of **1** in CH_2Cl_2. The photoluminescence spectrum of **1** was measured with excitation at 470 nm.

6. Raman spectra of 1-3

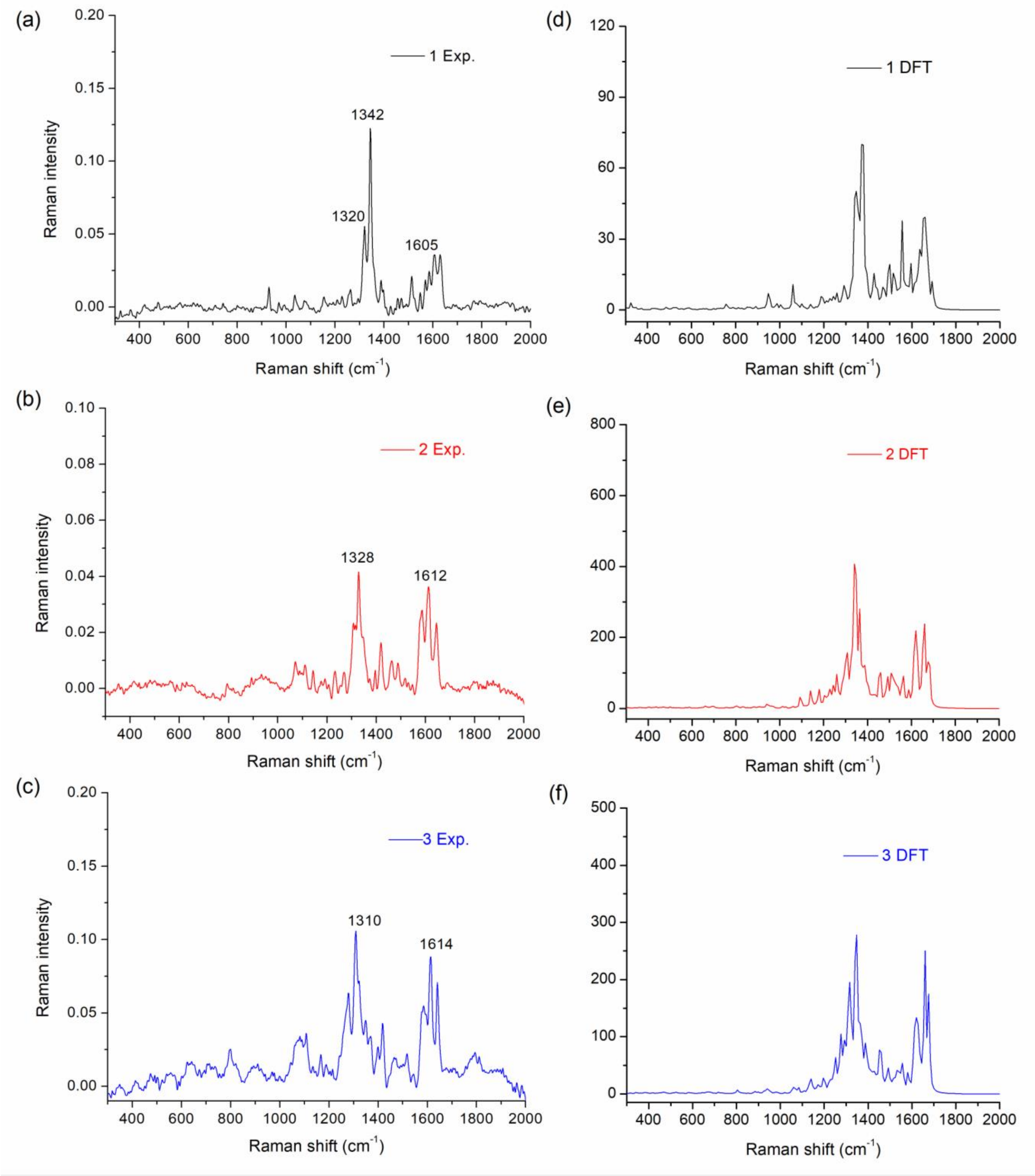

Figure S12. Solid state 1064 nm FT-Raman spectra of (a) **1**, (b) **2** and (c) **3** at room temperature. Calculated Raman spectra of (d) **1**, (e) **2** and (f) **3** (B3LYP-6-31G(d) standard basis set).

7. In situ spectroelectrochemistry

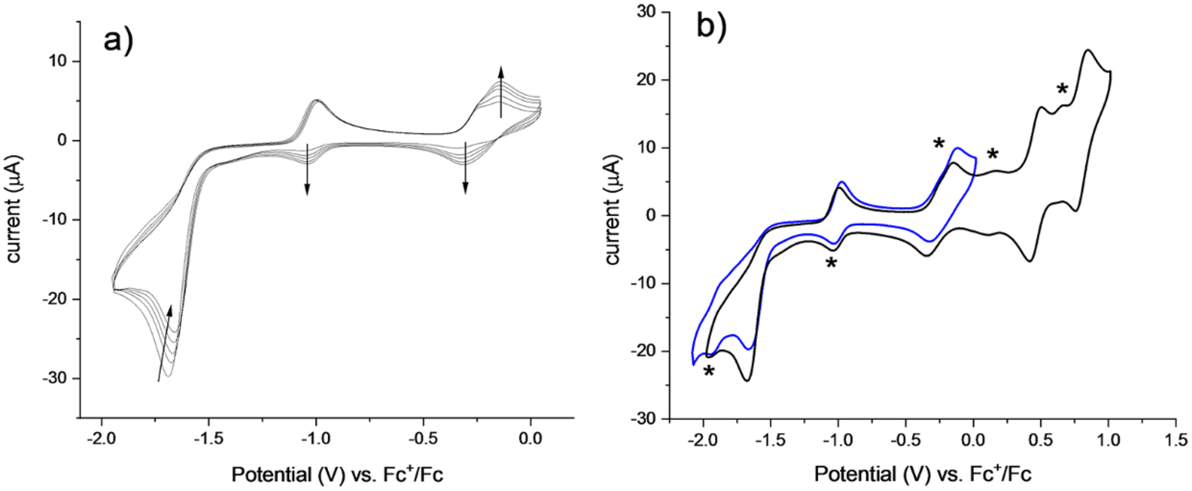

Figure S13. (a) Cyclic voltammogram of the compound **3** (five subsequent cycles). (b) Cyclic voltammogram of the compound **3** after potentiodynamic polarization in cathodic range. After potentiodynamic polarization of **3** in cathodic region, new electroactive structure(s) are formed. The redox events of new structures are marked by asterisk.

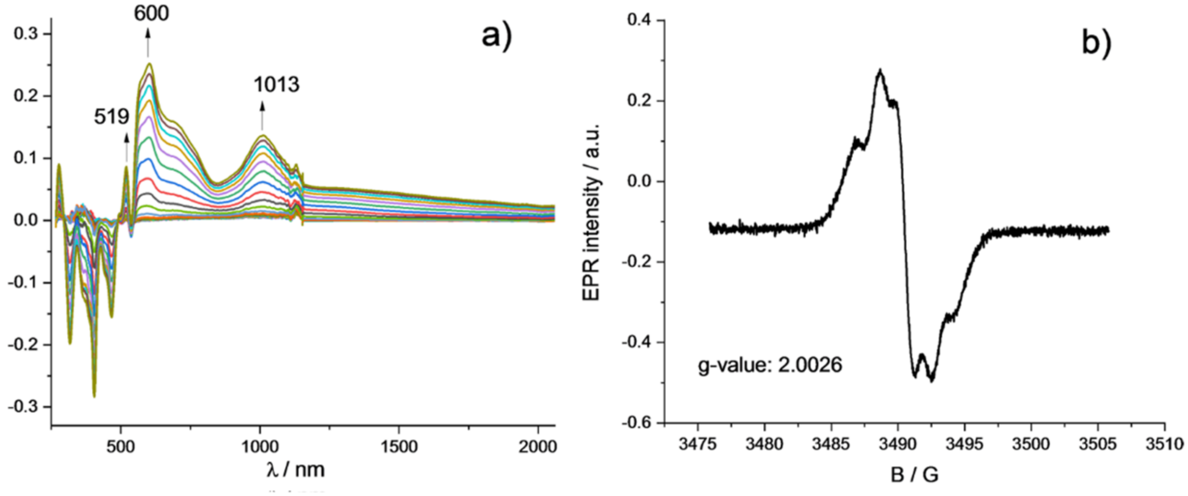

Figure S14. UV-Vis-NIR spectra (a) and EPR signal (b) measured during the oxidation of **2**.

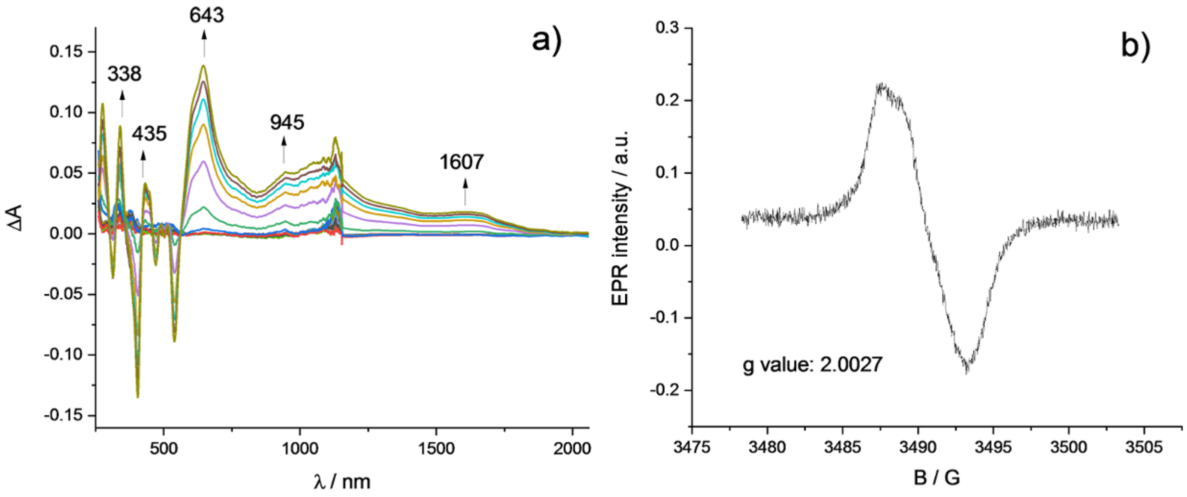

Figure S15. UV-Vis-NIR spectra (a) and EPR signal (b) measured during the reduction of **2**.

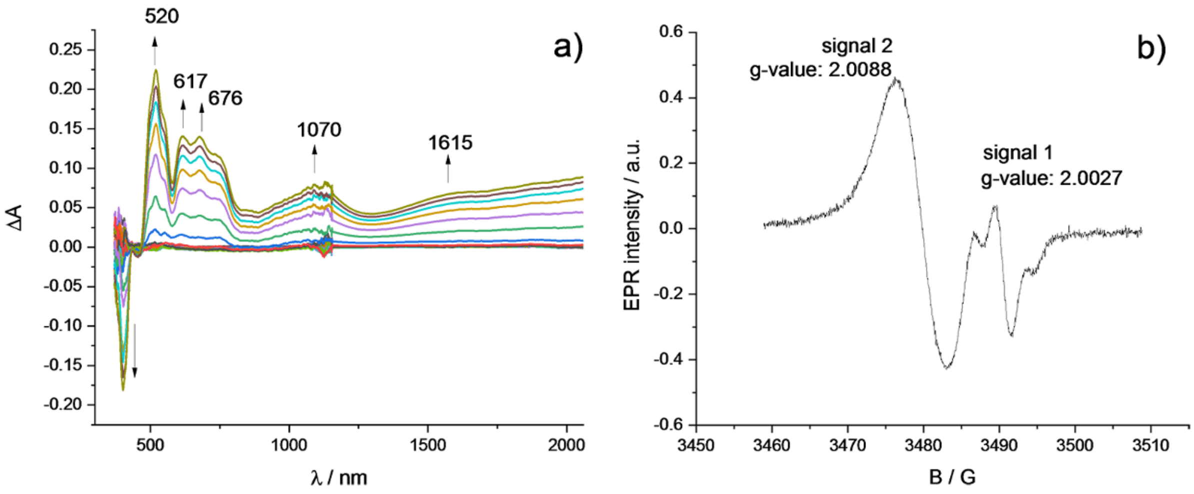

Figure S16. UV-Vis-NIR spectra (a) and EPR signals (b) measured during the oxidation of **3**.

8. DFT calculation details

All density functional theory (DFT) calculation was performed using the Gaussian 09 program.[4] The B3LYP functional was used for geometry optimization in the ground state and theoretical vibrational Raman spectra. The 6-31G(d) basis set was used for the C, and H atoms, while def2-SVP and its corresponding pseudopotential was used for I atom. All the geometry optimization was done in the gas phase and based on the single crystal structure. In order to simulate the UV-Vis spectra of the molecules TD-DFT calculations using B3LYP functional and 6-31G(d) basis set was used for the C, and H atoms, while def2-SVP and its corresponding pseudopotential was used for I atom. For better comparison to the experimental absorption spectra the polarity of the solvent dichloromethane was added.

Anisotropy of the induced current density (ACID) plots were calculated by Herges's method.[5] Nucleus independent chemical shifts (NICS) values were calculated using the standard gauge invariant atomic orbital (GIAO)[6] method at B3LYP functional. The 6-31+G(2d,p) basis set was used for the C, and H atoms, while def2-TZVP and its corresponding pseudopotential was used for I atom. All NICS values were averaged by two positions (above and below the plane) of each molecule.

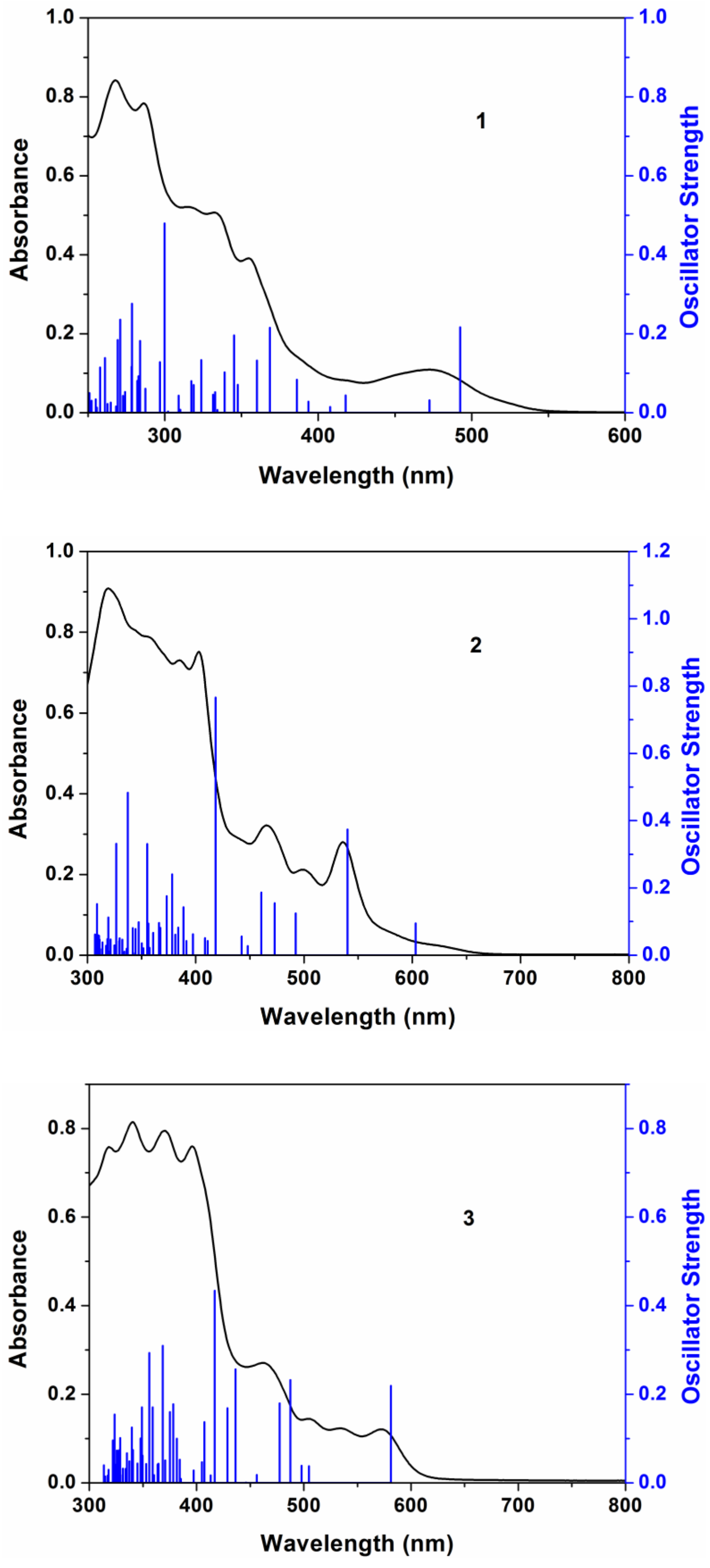

Figure S17. Experimental UV-Vis spectrum of **1**, **2** and **3** in CH_2Cl_2 (black plot) and calculated oscillator strength at TD-B3LYP/6-31+G(2d,p) level of theory (blue bar).

Table S2. TD-DFT calculation of **1** using B3LYP functional and 6-31G(d) basis set.

Excited state	Energy (eV)	Wavelength (nm)	Oscillator strength (f)	Description
1	2.5192	492.16	0.2162	HOMO -> LUMO 0.68260
2	2.6255	472.22	0.0316	HOMO-1 -> LUMO 0.69280
3	2.9690	417.60	0.0438	HOMO-2 -> LUMO 0.63139<br>HOMO-1 -> LUMO+1 0.11587<br>HOMO -> LUMO+1 0.23774
4	3.0427	407.49	0.0144	HOMO-2 -> LUMO -0.22325<br>HOMO -> LUMO+1 0.63579<br>HOMO -> LUMO+2 0.10889<br>HOMO -> LUMO+3 -0.11628
5	3.1518	393.38	0.0280	HOMO-3 -> LUMO -0.14004<br>HOMO-1 -> LUMO+1 0.27576<br>HOMO-1 -> LUMO+3 -0.10375<br>HOMO -> LUMO+1 -0.11243<br>HOMO -> LUMO+2 0.60401
6	3.2134	385.84	0.0832	HOMO-3 -> LUMO 0.67334<br>HOMO -> LUMO+2 0.14857
7	3.3672	368.21	0.2153	HOMO-5 -> LUMO 0.13991<br>HOMO-2 -> LUMO -0.10344<br>HOMO-1 -> LUMO+1 0.58675<br>HOMO-1 -> LUMO+2 -0.14072<br>HOMO -> LUMO+2 -0.25901
8	3.4460	359.79	0.1320	HOMO-2 -> LUMO -0.10320<br>HOMO-1 -> LUMO+1 0.15176<br>HOMO-1 -> LUMO+2 0.53524<br>HOMO -> LUMO+3 0.36559
9	3.5699	347.31	0.0705	HOMO-4 -> LUMO 0.22697<br>HOMO-2 -> LUMO+1 -0.21330<br>HOMO-2 -> LUMO+2 0.25688<br>HOMO-2 -> LUMO+4 -0.10332<br>HOMO-1 -> LUMO+2 -0.30505<br>HOMO -> LUMO+3 0.43245<br>HOMO -> LUMO+4 -0.10217
10	3.5943	344.94	0.1960	HOMO-5 -> LUMO 0.34577<br>HOMO-4 -> LUMO -0.34728<br>HOMO-2 -> LUMO+1 0.35972<br>HOMO-1 -> LUMO+2 -0.19735<br>HOMO -> LUMO+3 0.23770

Table S3. TD-DFT calculation of **2** using B3LYP functional and 6-31G(d) basis set.

Excited state	Energy (eV)	Wavelength (nm)	Oscillator strength (f)	Description

1	2.0571	602.71	0.0950	HOMO-1 -> LUMO 0.11092<br>HOMO -> LUMO 0.68645
2	2.2970	539.76	0.3737	HOMO-1 -> LUMO 0.67737<br>HOMO -> LUMO+1 -0.11632
3	2.5201	491.98	0.1243	HOMO-3 -> LUMO 0.12341<br>HOMO-2 -> LUMO 0.66473
4	2.6238	472.54	0.1546	HOMO-3 -> LUMO 0.64283<br>HOMO-2 -> LUMO -0.12509<br>HOMO -1-> LUMO+1 0.13566<br>HOMO -> LUMO+2 0.12066<br>HOMO -> LUMO+3 0.10892
5	2.6947	460.10	0.1866	HOMO-4 -> LUMO -0.29925<br>HOMO-1 -> LUMO 0.10313<br>HOMO -> LUMO+1 0.58840
6	2.7699	447.61	0.0276	HOMO-4 -> LUMO 0.61057<br>HOMO-1 -> LUMO+1 -0.11811<br>HOMO -> LUMO+1 0.27835
7	2.8043	442.12	0.0559	HOMO-3 -> LUMO -0.15674<br>HOMO-2 -> LUMO 0.10204<br>HOMO-2 -> LUMO+1 0.15035<br>HOMO-1 -> LUMO+1 0.27285<br>HOMO-1 -> LUMO+3 -0.10093<br>HOMO -> LUMO+2 0.54448<br>HOMO -> LUMO+3 0.13159
8	2.9658	418.04	0.7664	HOMO-5 -> LUMO 0.20788<br>HOMO-3 -> LUMO -0.10882<br>HOMO-1 -> LUMO+1 0.54938<br>HOMO -> LUMO+2 -0.29746
9	3.0180	410.82	0.0423	HOMO-5 -> LUMO 0.57034<br>HOMO-1 -> LUMO+1 -0.11081<br>HOMO -> LUMO+2 0.17055<br>HOMO -> LUMO+3 -0.31588
10	3.0367	408.29	0.0509	HOMO-5 -> LUMO 0.27734<br>HOMO-2 -> LUMO+1 0.13342<br>HOMO -1-> LUMO+1 -0.16066<br>HOMO-1 -> LUMO+2 0.36386<br>HOMO -> LUMO+3 0.44141

Table S4. TD-DFT calculation of **3** using B3LYP functional and 6-31G(d) basis set.

Excited state	Energy (eV)	Wavelength (nm)	Oscillator strength (f)	Description
1	2.1350	580.71	0.2191	HOMO-> LUMO 0.68879<br>HOMO-> LUMO+1 0.10206
2	2.4577	504.47	0.0379	HOMO-4-> LUMO 0.19224

				HOMO-3-> LUMO 0.23225<br>HOMO-2-> LUMO 0.45198<br>HOMO-1-> LUMO 0.42286
3	2.4915	497.63	0.0389	HOMO-3 -> LUMO -0.13973<br>HOMO-2 -> LUMO -0.27212<br>HOMO-1 -> LUMO 0.43631<br>HOMO -> LUMO+1 0.43422
4	2.5452	487.13	0.2323	HOMO-3 -> LUMO 0.21853<br>HOMO-2 -> LUMO 0.21472<br>HOMO-1 -> LUMO -0.31143<br>HOMO -> LUMO+1 0.51722
5	2.5985	477.13	0.1796	HOMO-3 -> LUMO 0.57059<br>HOMO-2 -> LUMO -0.32021<br>HOMO -> LUMO+2 -0.16677
6	2.7195	455.9	0.0180	HOMO-4 -> LUMO 0.27569<br>HOMO-2 -> LUMO -0.12042<br>HOMO-2 -> LUMO+1 0.36912<br>HOMO-1 -> LUMO+1 -0.17868<br>HOMO -> LUMO+2 0.32347<br>HOMO -> LUMO+3 -0.28123<br>HOMO -> LUMO+4 -0.12599
7	2.7808	445.86	0.0010	HOMO-4 -> LUMO 0.57010<br>HOMO-2 -> LUMO -0.19037<br>HOMO-2 -> LUMO+1 -0.11561<br>HOMO-1 -> LUMO+1 0.11412<br>HOMO -> LUMO+3 0.23822
8	2.8437	436.00	0.2564	HOMO-2 -> LUMO+1 0.10035<br>HOMO-1 -> LUMO+1 0.57451<br>HOMO -> LUMO+2 0.27596
9	2.8934	428.51	0.1687	HOMO-4 -> LUMO+1 0.11776<br>HOMO-2 -> LUMO+1 -0.16055<br>HOMO -> LUMO+1 -0.28122<br>HOMO -> LUMO+2 0.38422<br>HOMO -> LUMO+3 0.38322<br>HOMO -> LUMO+4 -0.17923
10	2.9766	416.52	0.4341	HOMO-4 -> LUMO+1 0.21493<br>HOMO-3 -> LUMO+1 0.18944<br>HOMO-2 -> LUMO+1 0.46674<br>HOMO -> LUMO+2 -0.20669<br>HOMO-> LUMO+3 0.34521

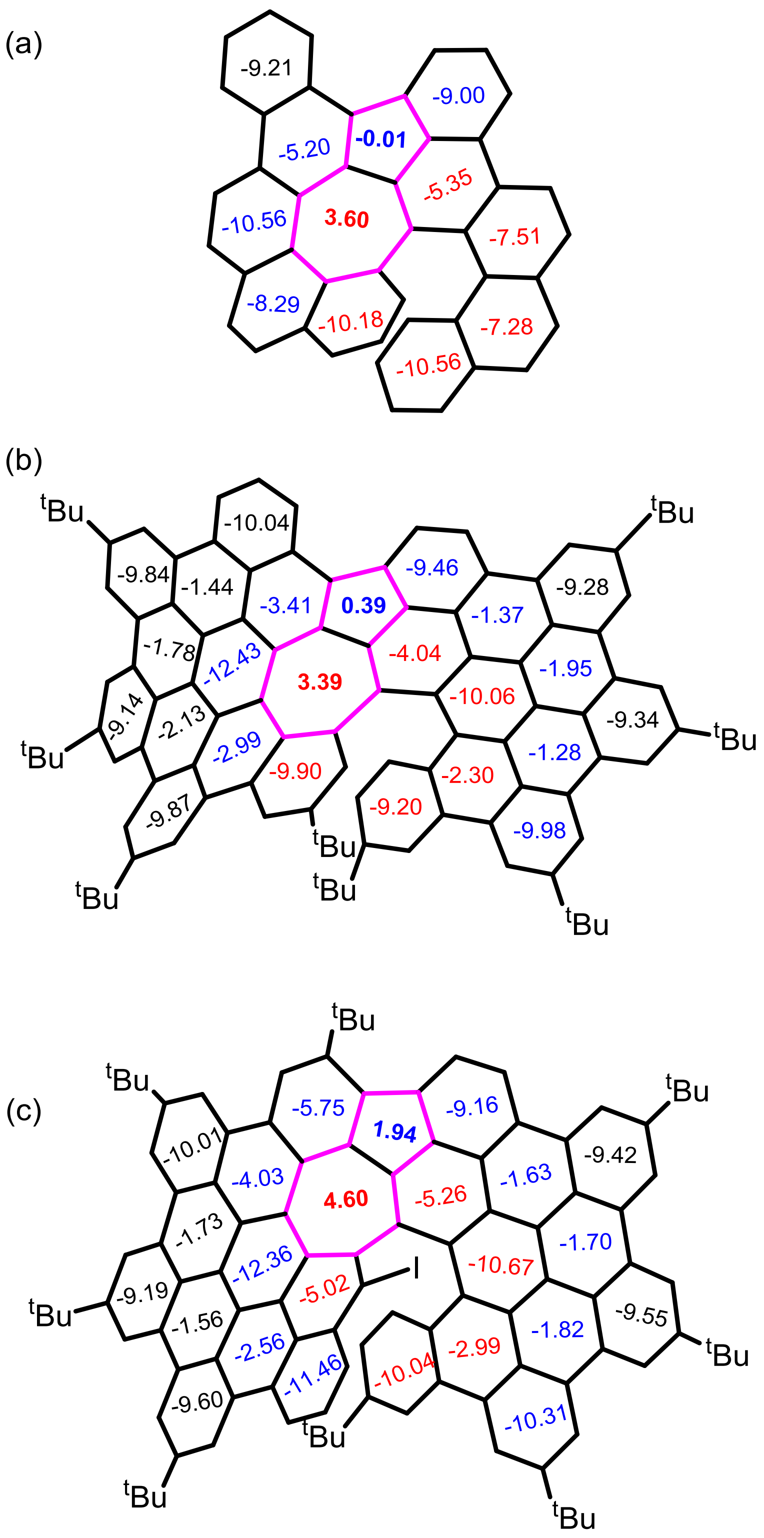

Figure S18. NICS(1) values of (a) **1**, (b) **2** and (c) **3**, calculated at the GIAO-B3LYP/6-31+G(2d,p) level of theory.

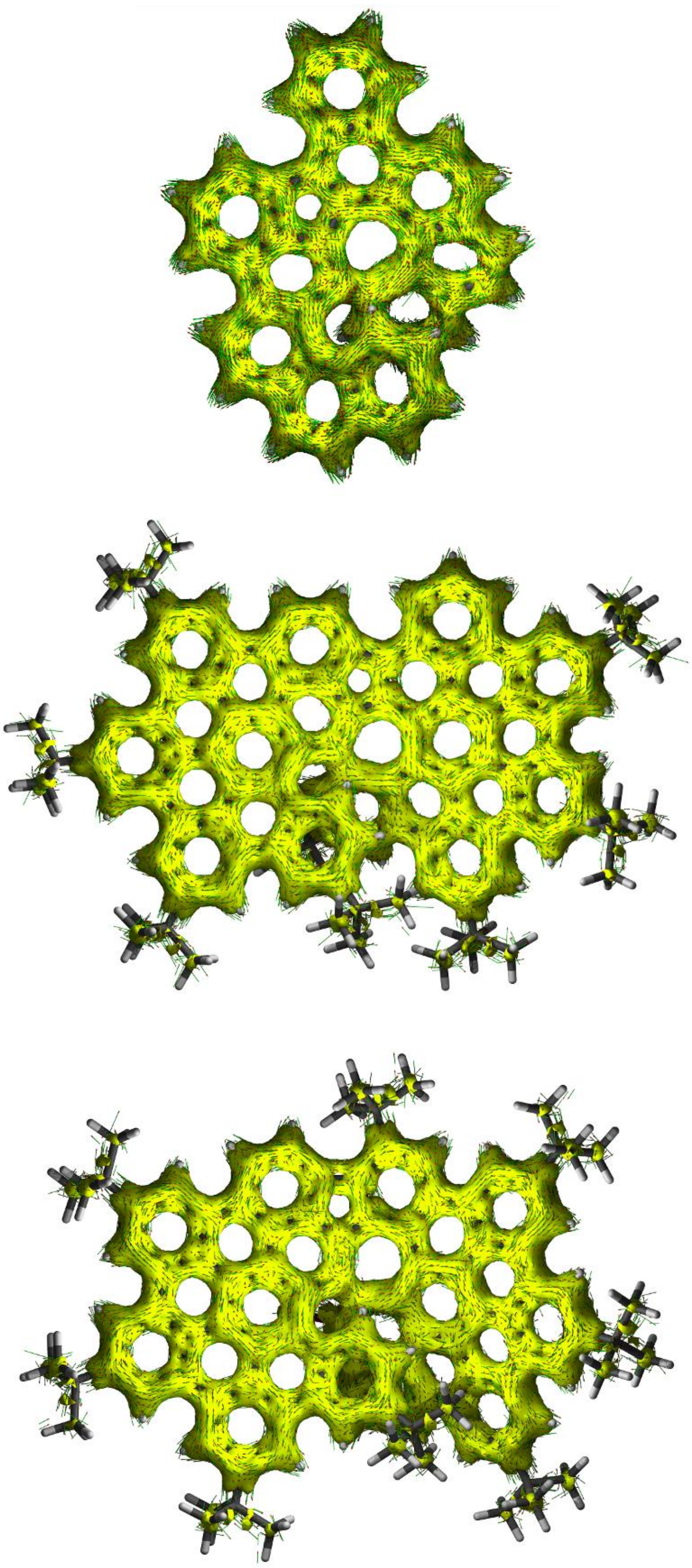

Figure S19. Enlarged ACID plot of **1** (top), **2** (middle) and **3** (bottom) (isosurface value = 0.05).

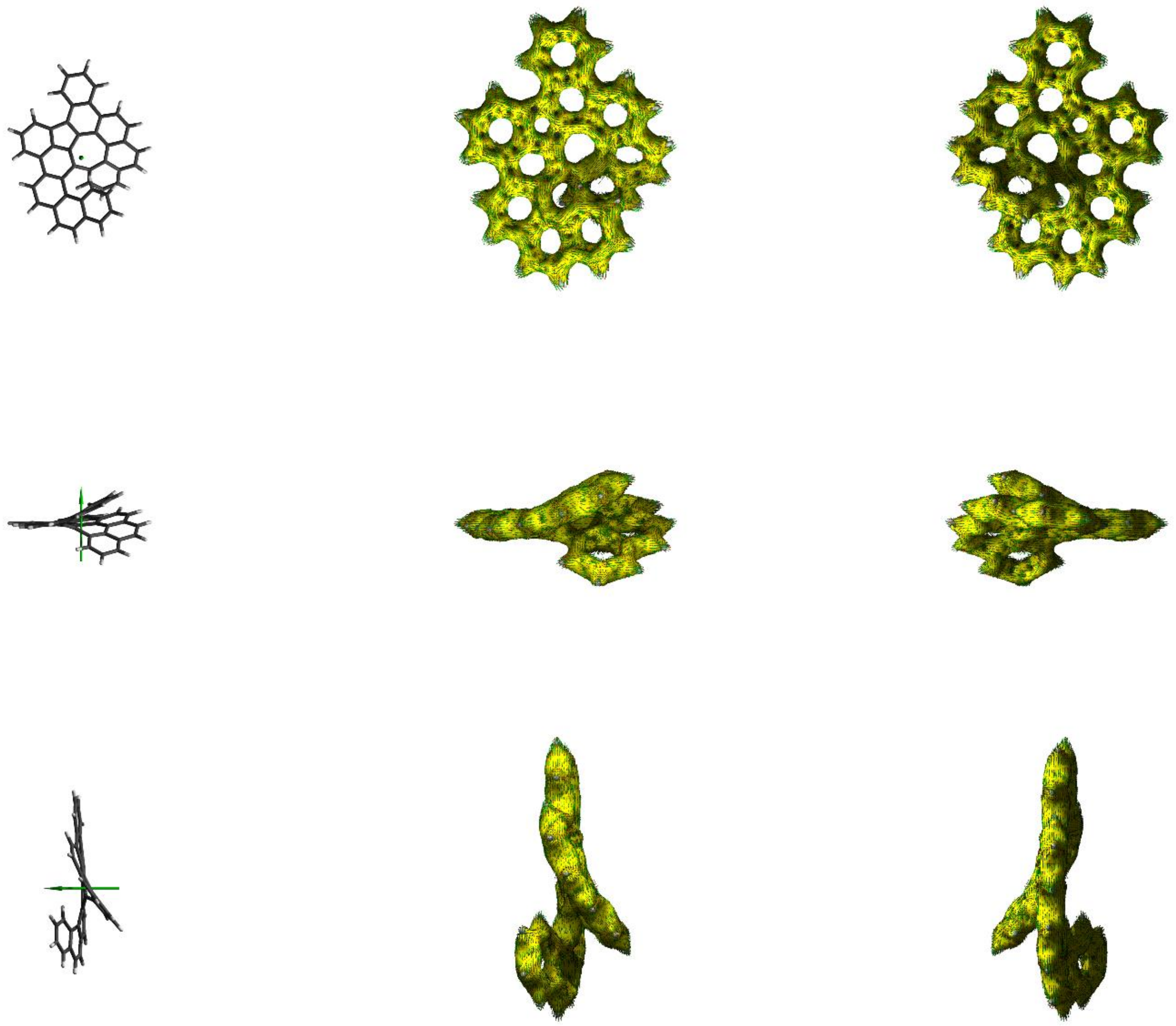

Figure S20. ACID plots of **1** viewed from different angles. The green arrow indicates the magnetic field. Isovalue is 0.05.

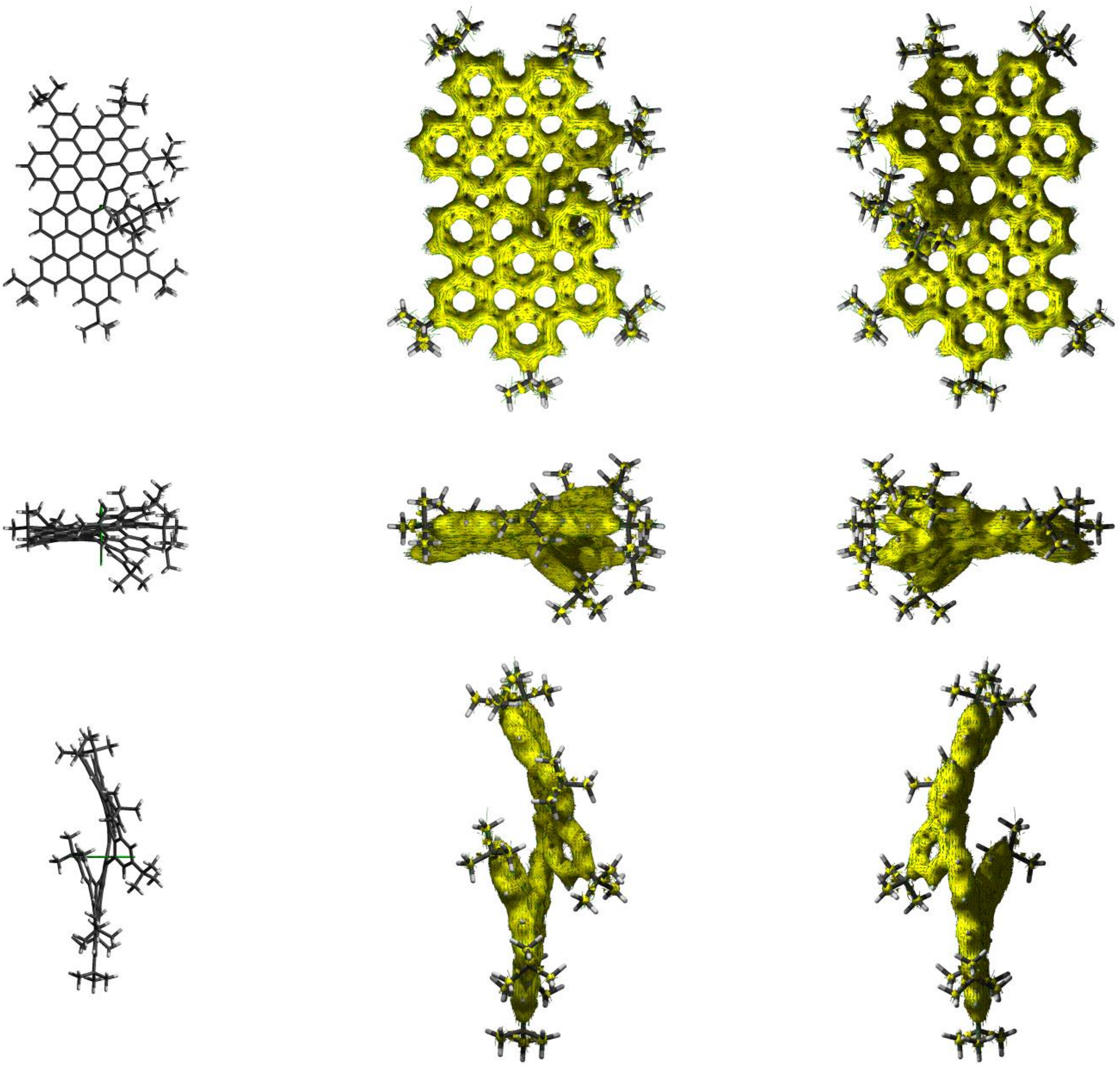

Figure S21. ACID plots of **2** viewed from different angles. The green arrow indicates the magnetic field. Isovalue is 0.05.

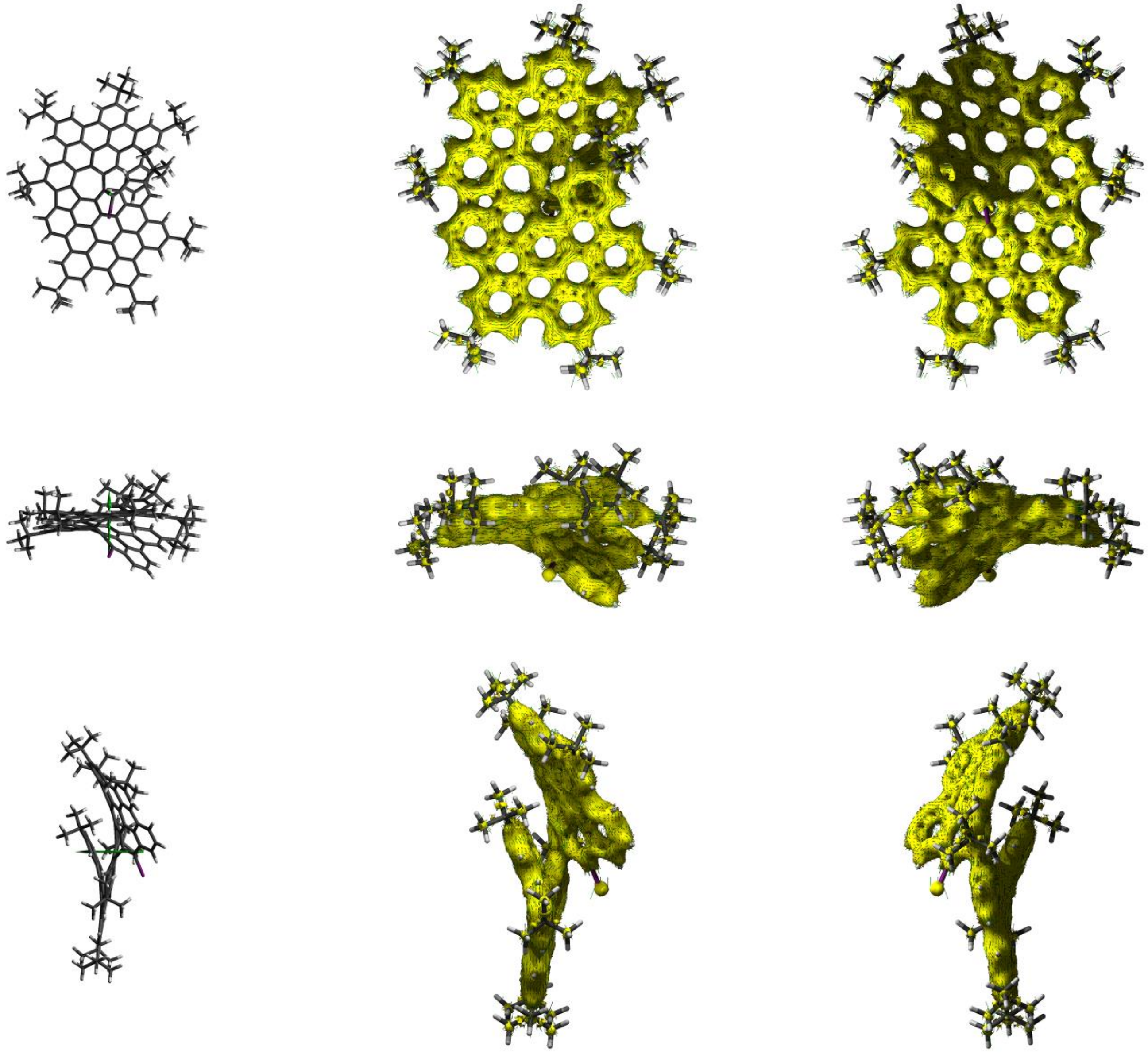

Figure S22. ACID plots of **3** viewed from different angles. The green arrow indicates the magnetic field. Isovalue is 0.05.

9. NMR spectra

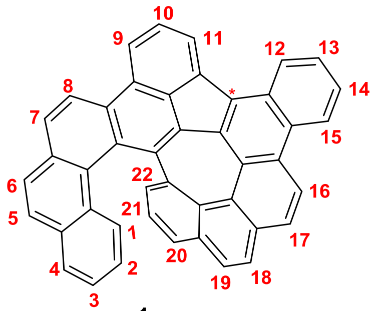

1

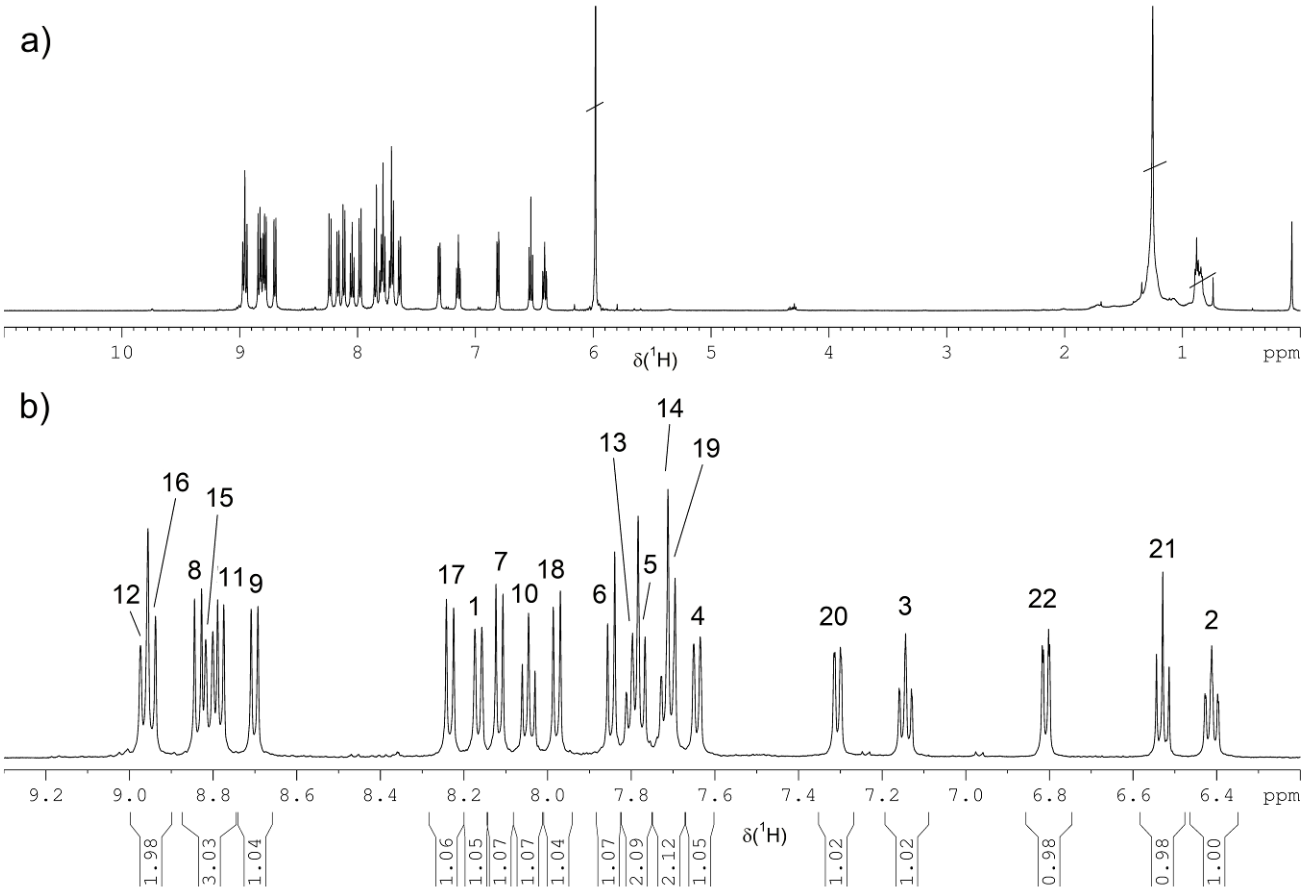

Figure S23. ^{1}H NMR spectrum of **1** (500 MHz, $C_2D_2Cl_4$, 30 °C): a) overview and b) enlarged region.

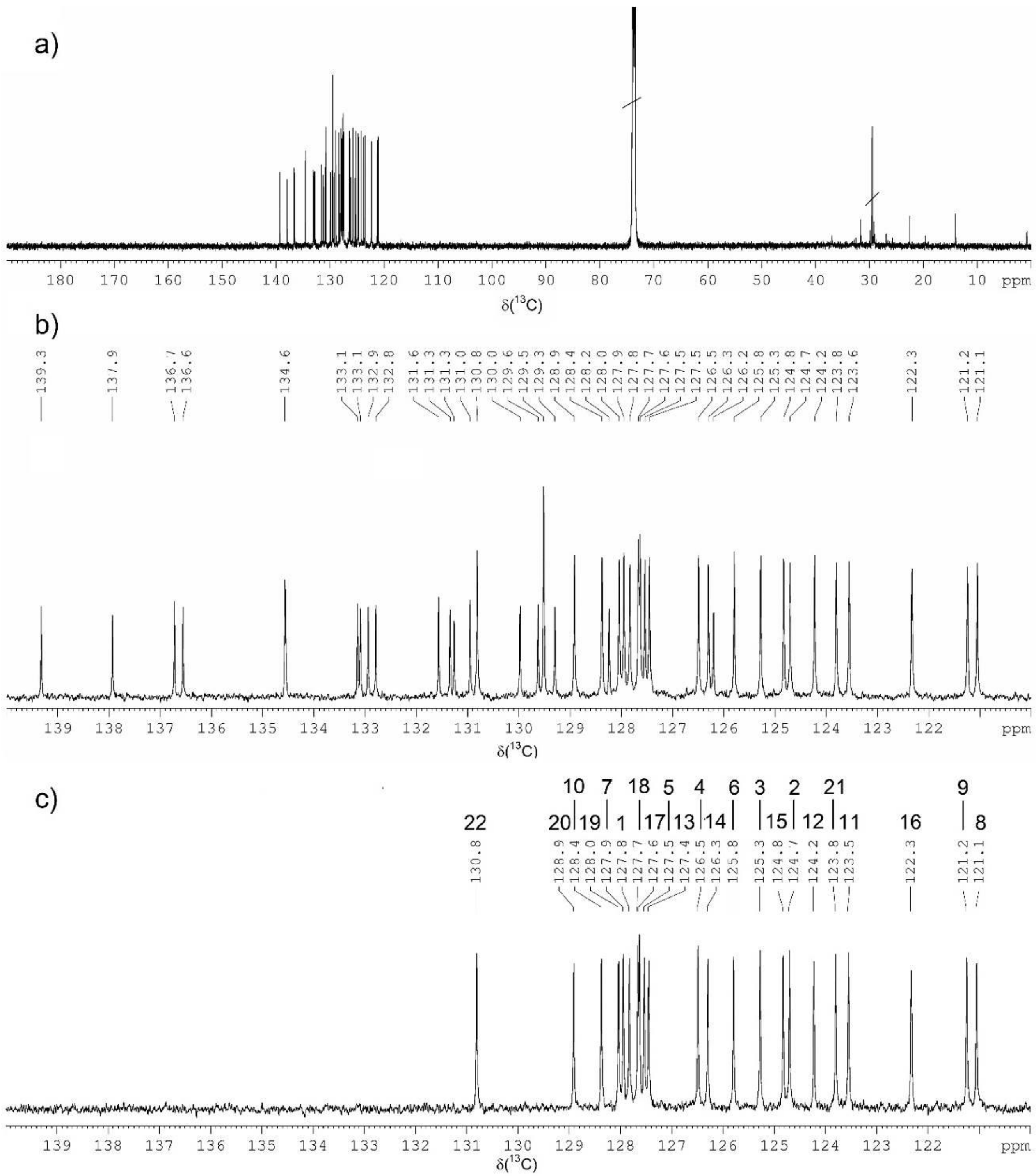

Figure S24. ^{13}C NMR spectra of **1** (125 MHz, $C_2D_2Cl_4$, 30 °C): a) overview, b) enlarged region and c) DEPT-135 spectrum showing only CH signals.

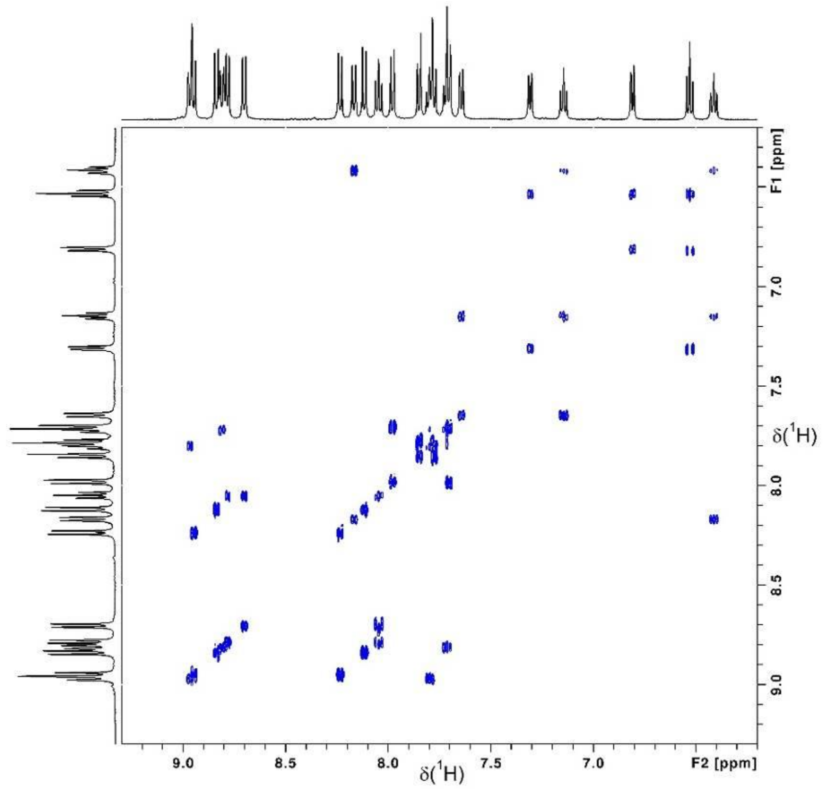

Figure S25. COSY spectrum of **1** (500 MHz, $C_2D_2Cl_4$, 30 °C).

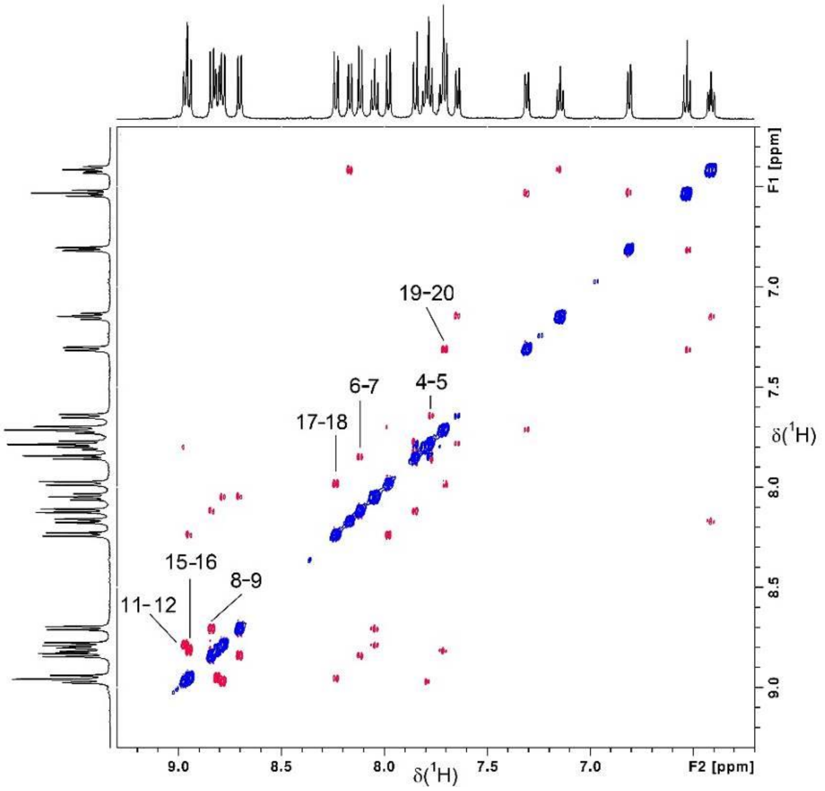

Figure S26. ROESY spectrum of **1** (500 MHz, $C_2D_2Cl_4$, 30 °C). Besides the assigned correlation peaks between protons of different spin systems there are also correlation peaks between protons within the same spin system.

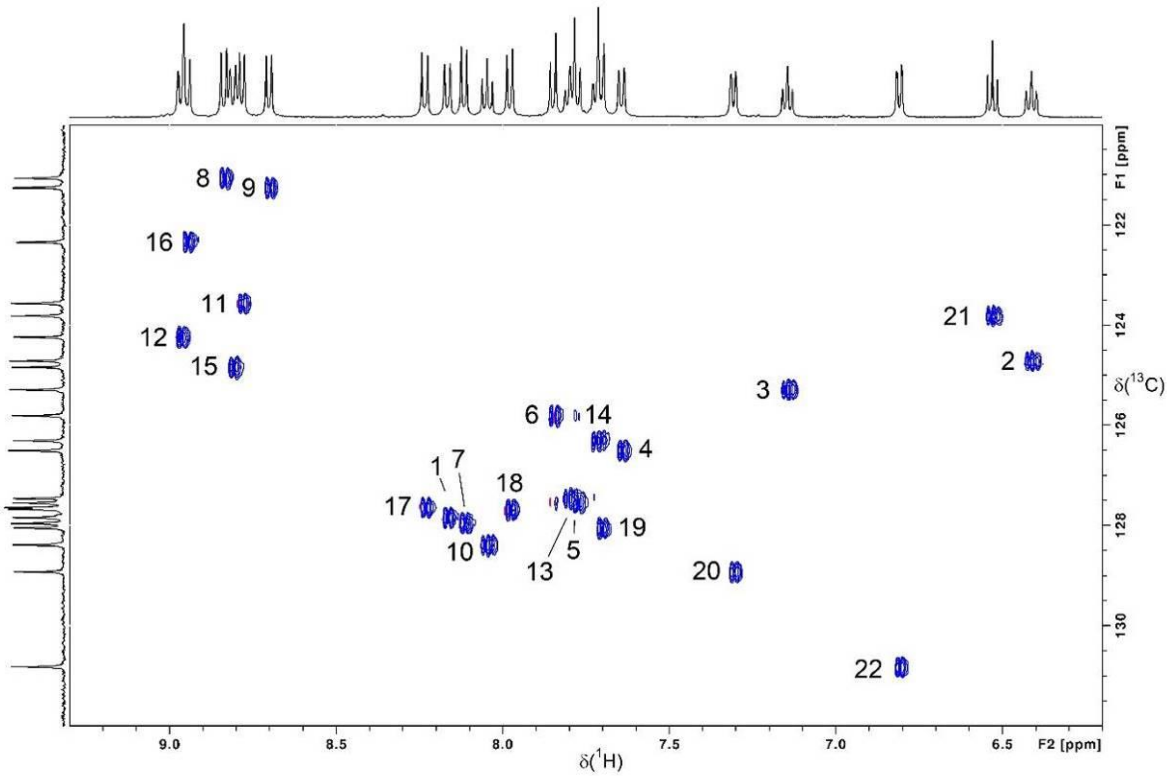

Figure S27. HSQC spectrum of **1** ($C_2D_2Cl_4$, 30 °C). The F1 dimension shows the DEPT-135 spectrum (comp. Fig. S24c).

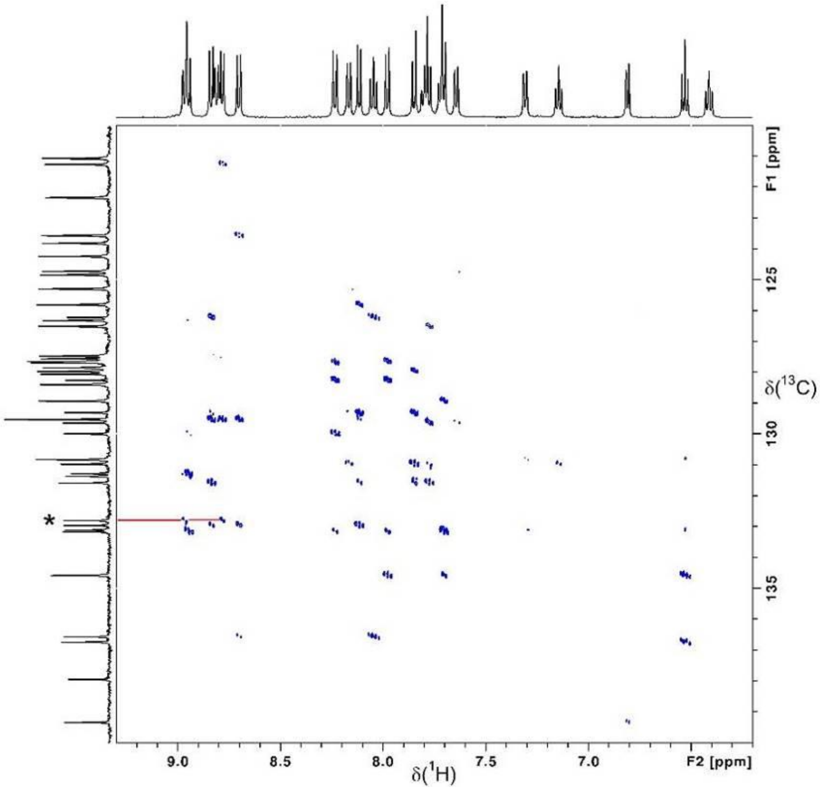

Figure S28. HMBC spectrum of **1** ($C_2D_2Cl_4$, 30 °C). The red line marks the correlation of H11 and H12 to the same quarternary carbon (*).

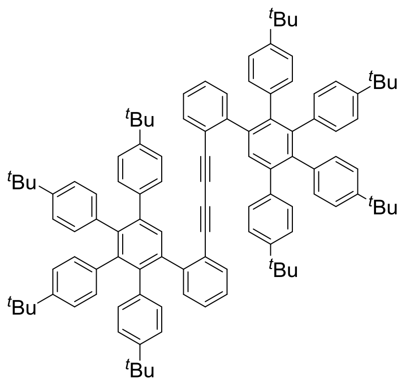

12

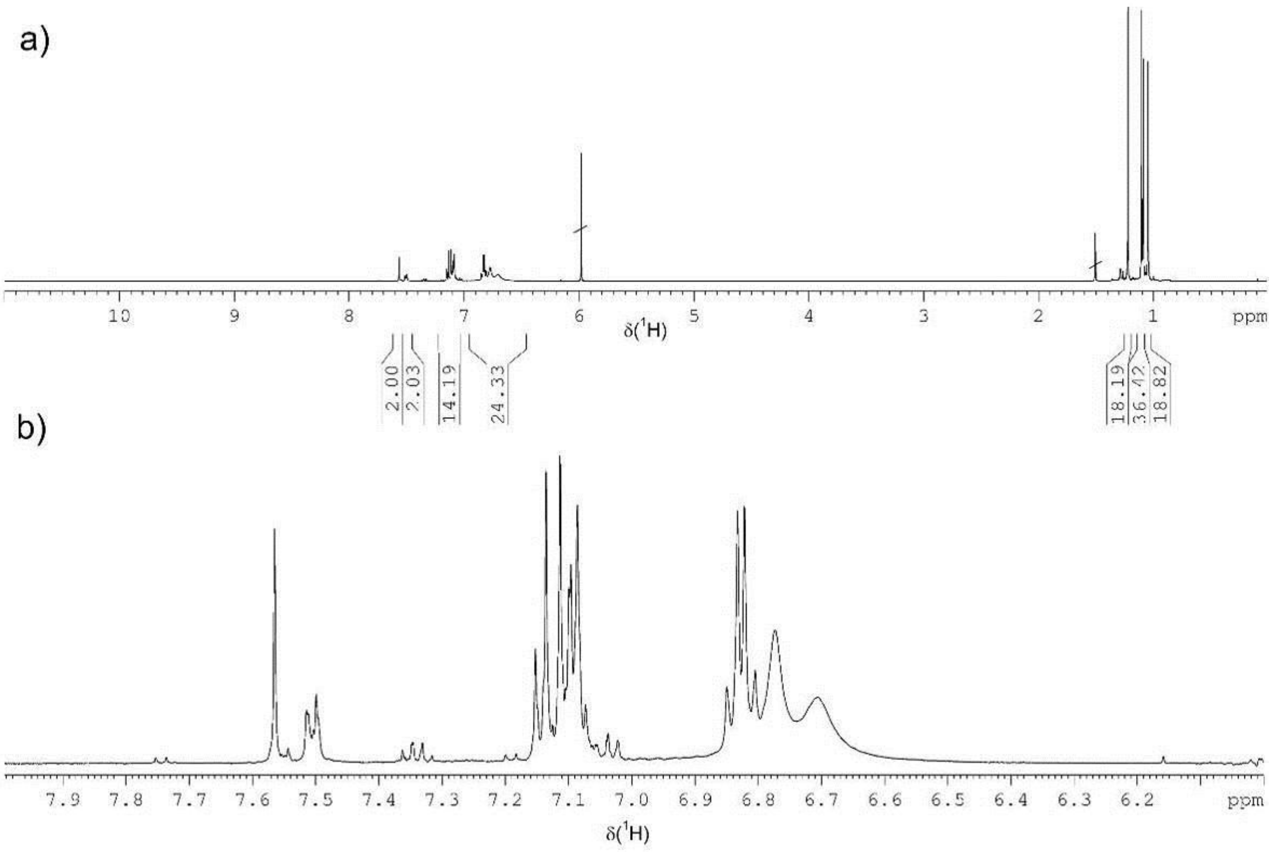

Figure S29. ^{1}H NMR spectrum of **12** (500 MHz, $C_2D_2Cl_4$, 60 °C): a) overview and b) enlarged region.

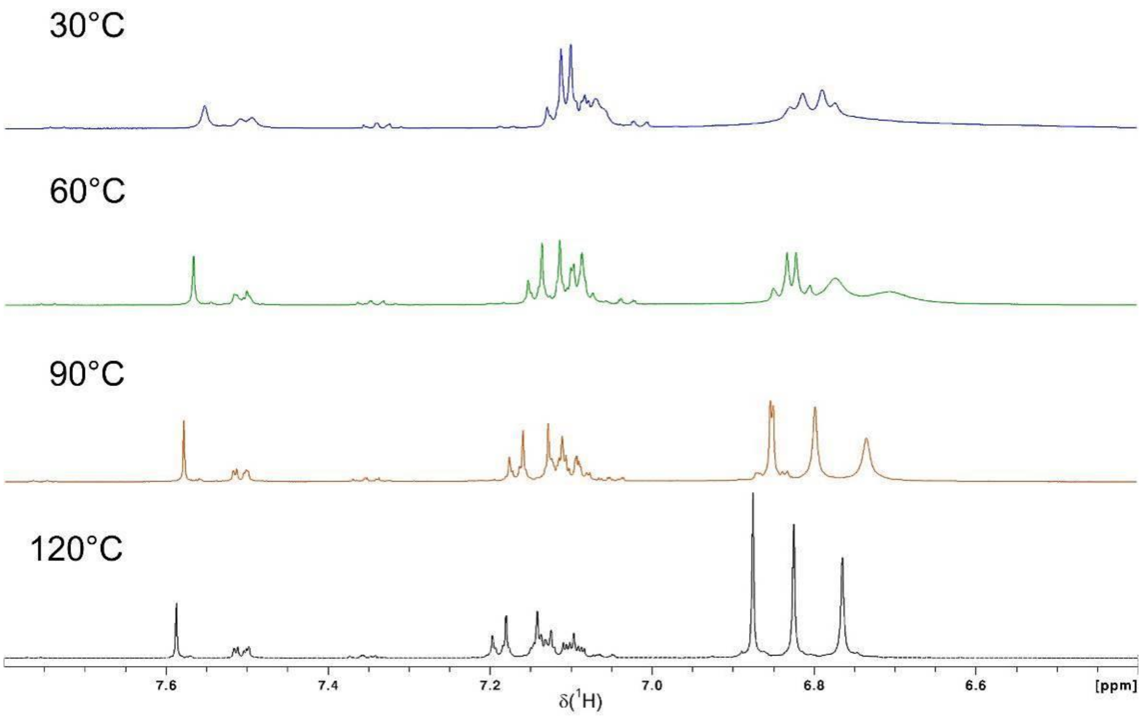

Figure S30. VT ^{1}H NMR spectra of **12** (500 MHz, $C_2D_2Cl_4$).

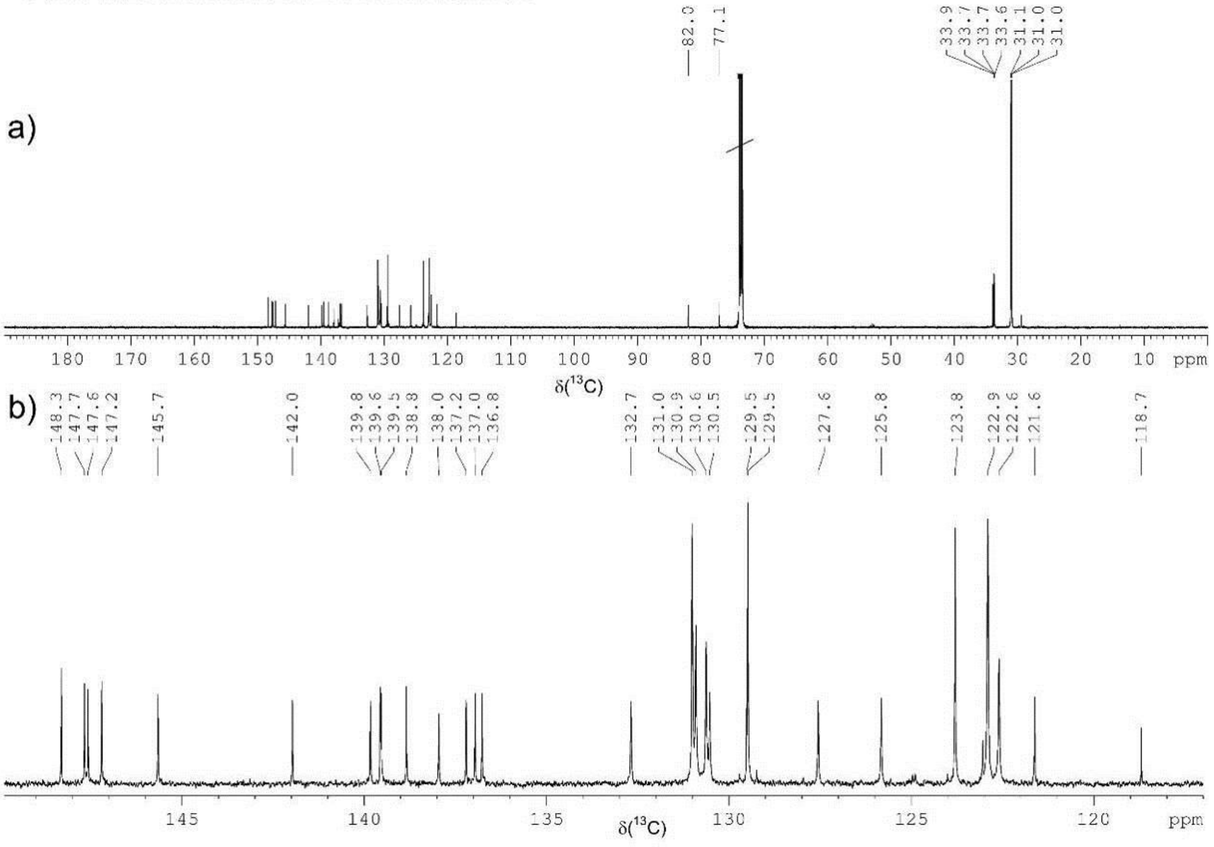

Figure S31. ^{13}C NMR spectra of **12** (125 MHz, $C_2D_2Cl_4$, 60 °C): a) overview and b) region of aromatic carbon signals.

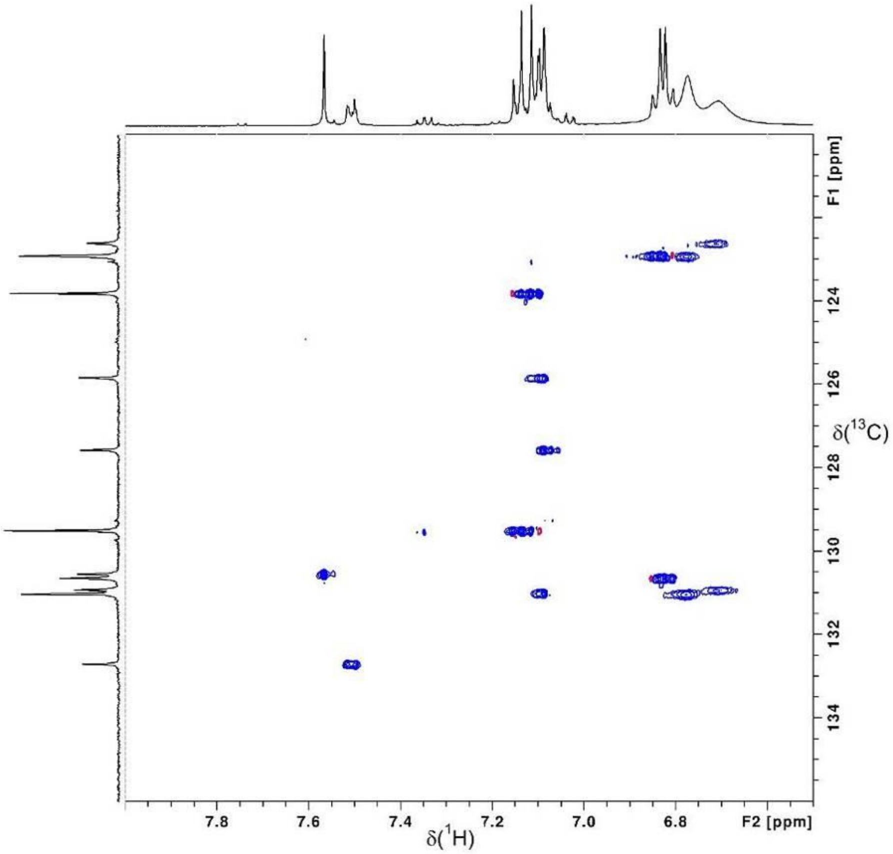

Figure S32. HSQC spectrum (region) of **12** ($C_2D_2Cl_4$, 60 °C). The F1 dimension shows the DEPT-135 spectrum.

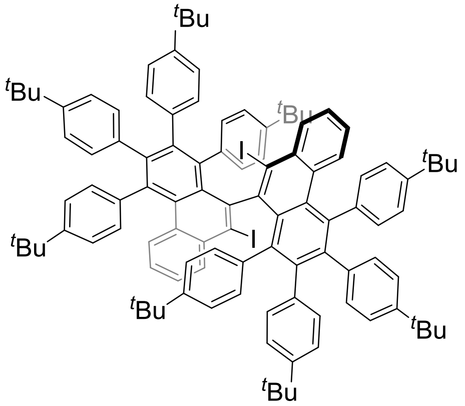

13

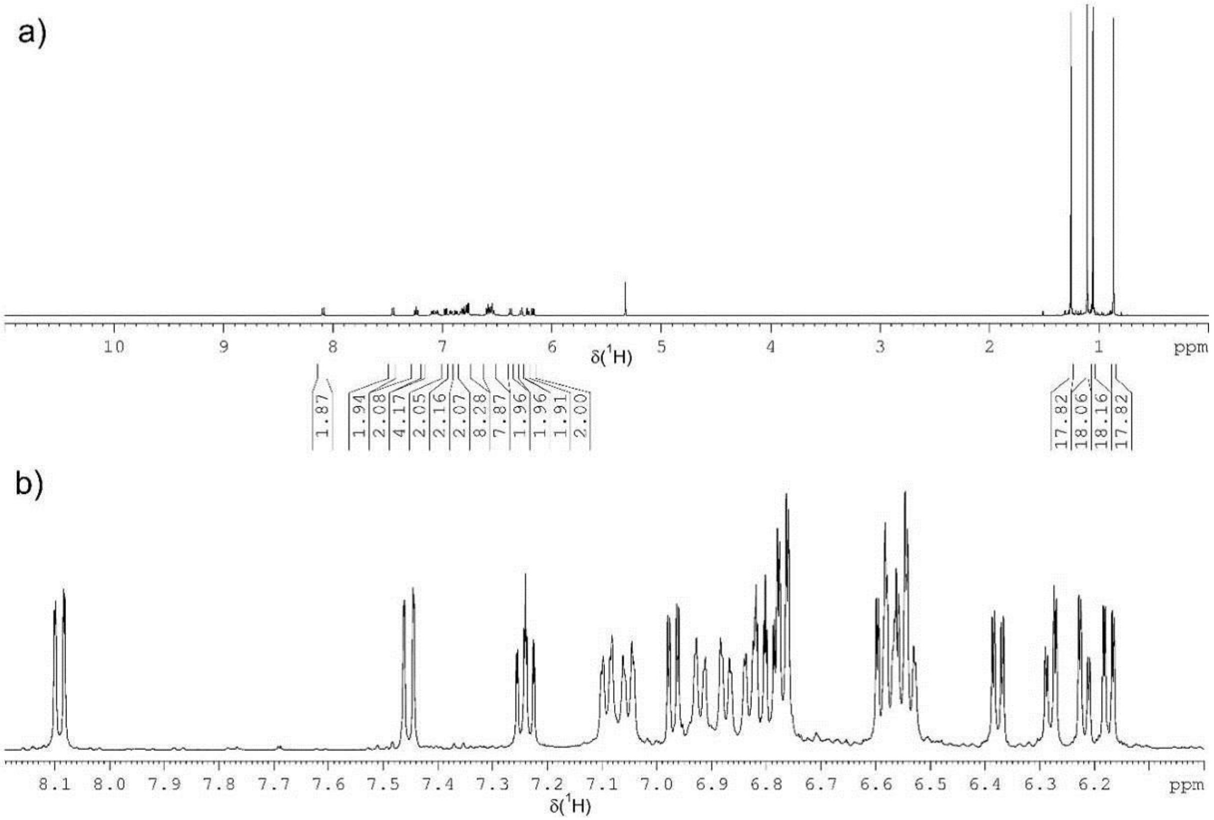

Figure S33. ^{1}H NMR spectrum of **13** (500 MHz, CD_2Cl_2): a) overview and b) enlarged region.

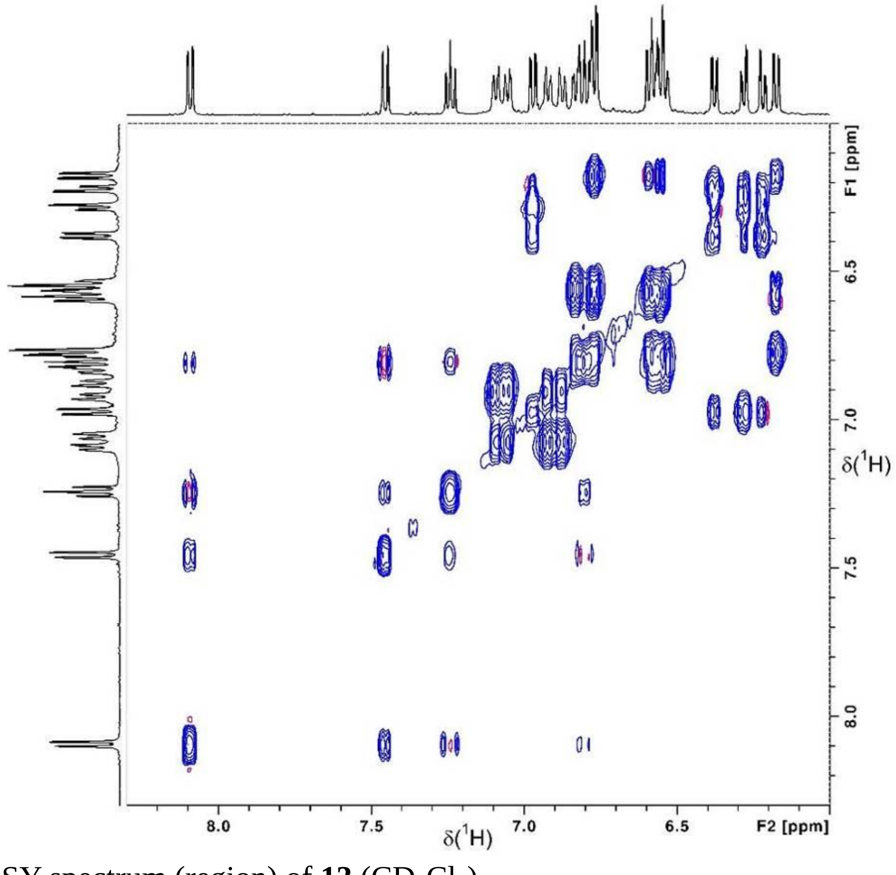

Figure S34. TOCSY spectrum (region) of **13** (CD_2Cl_2).

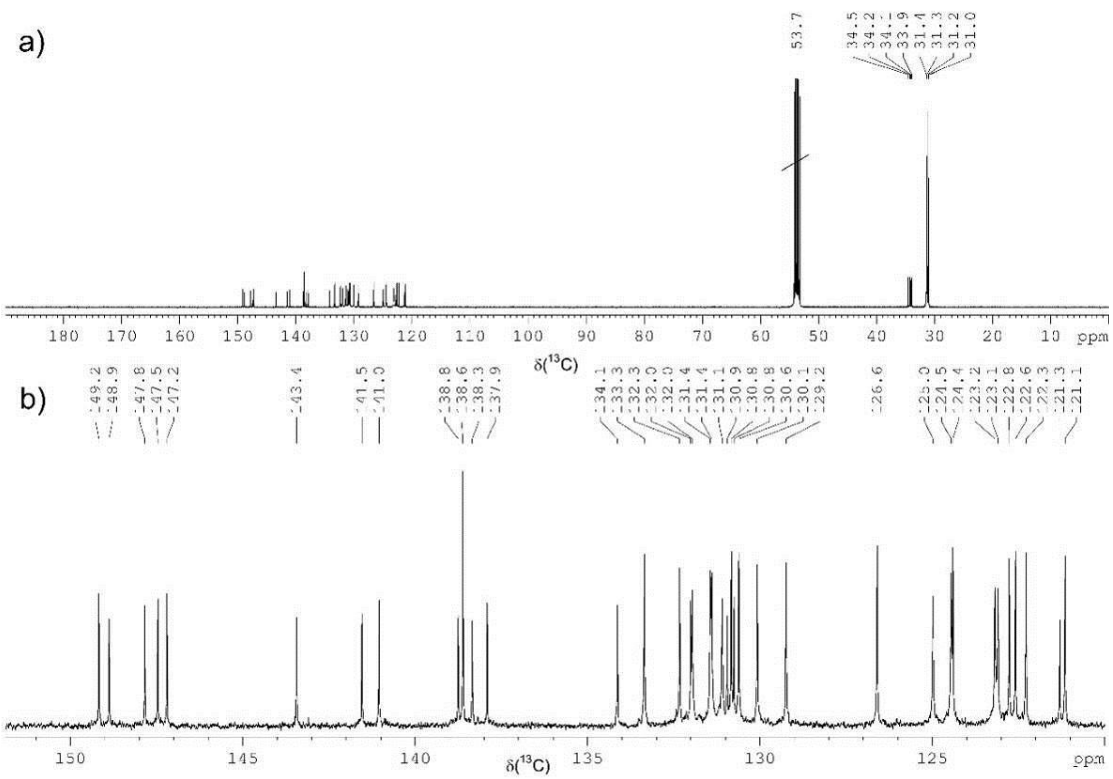

Figure S35. ^{13}C NMR spectra of **13** (125 MHz, CD_2Cl_2): a) overview and b) region of aromatic carbon signals.

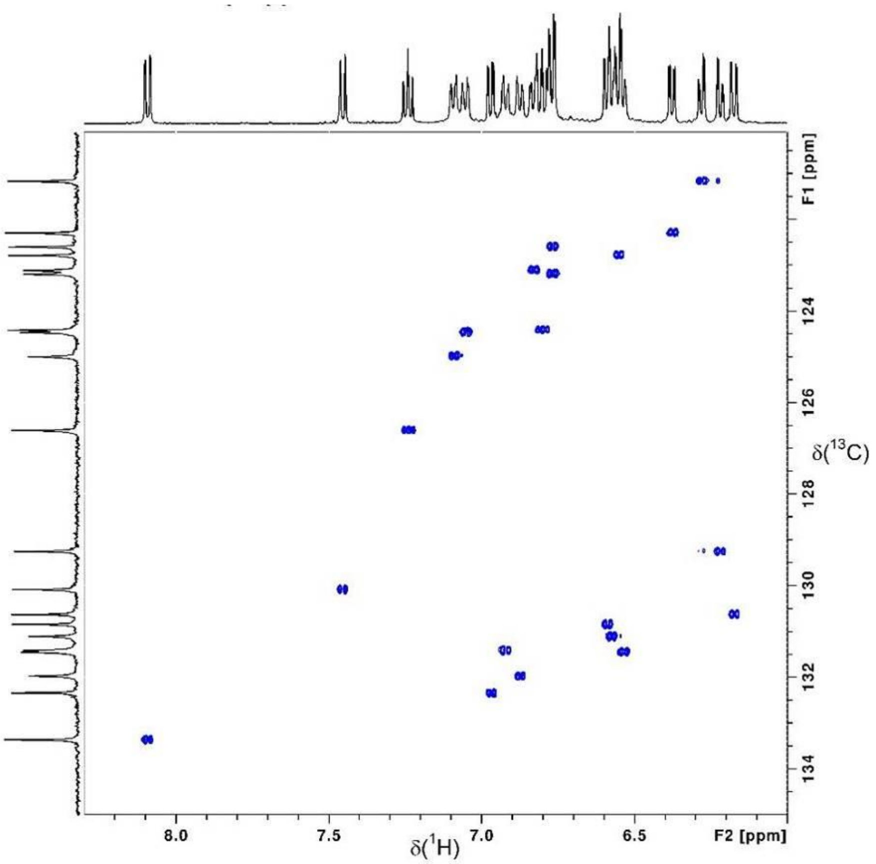

Figure S36. HSQC spectrum (region) of **13** (CD_2Cl_2). The F1 dimension shows the DEPT-135 spectrum.

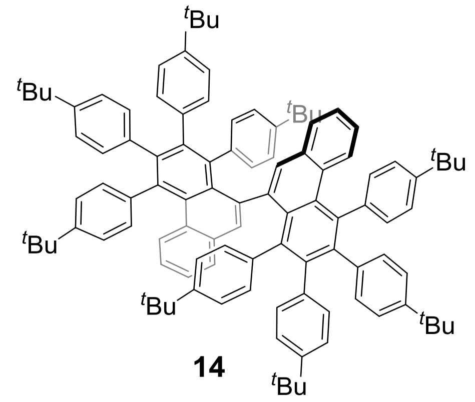

14

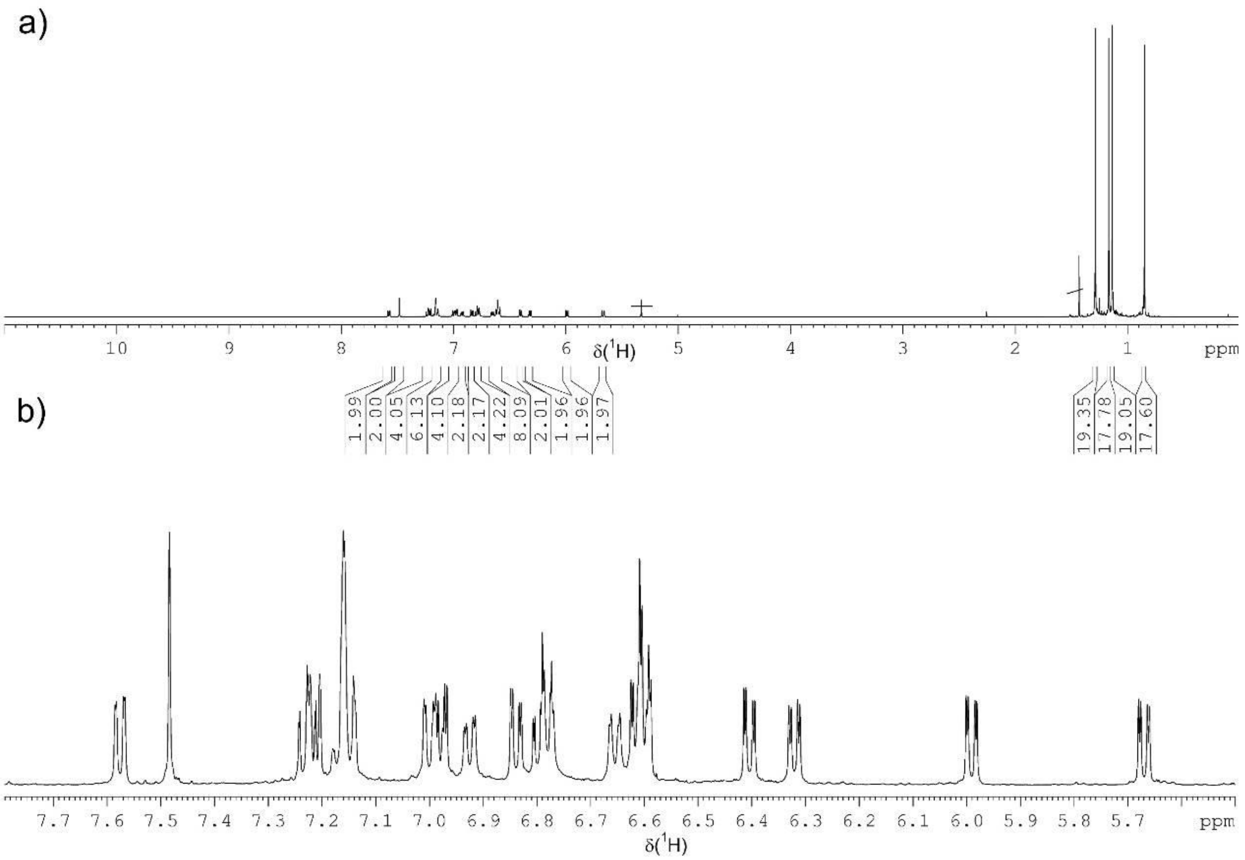

Figure S37. ^{1}H NMR spectrum of **14** (500 MHz, CD_2Cl_2): a) overview and b) enlarged region.

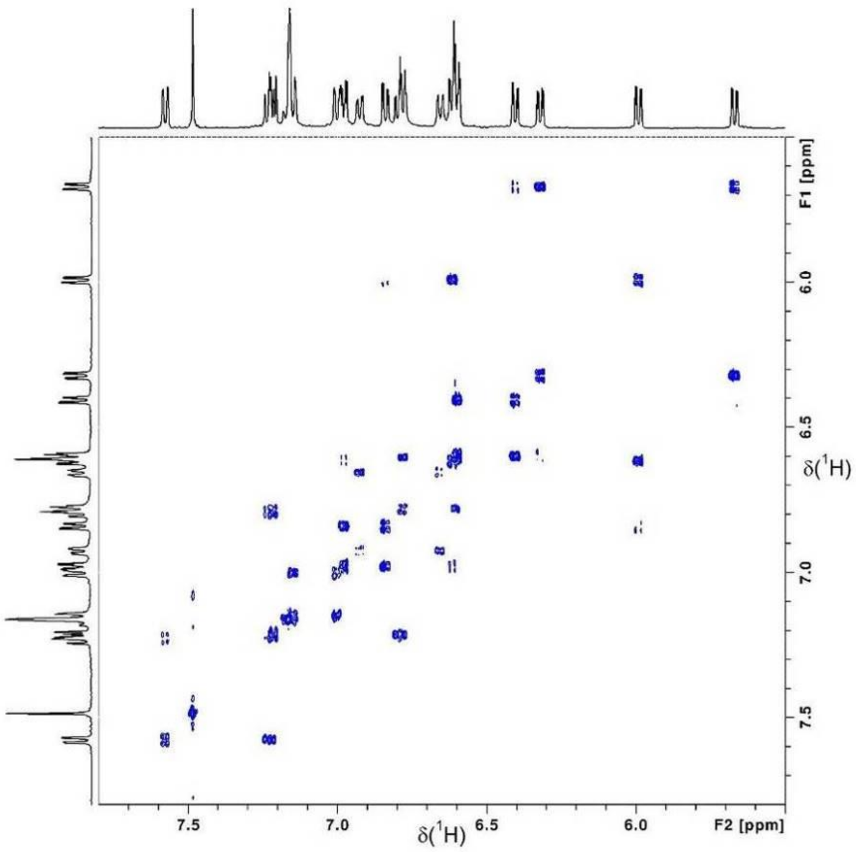

Figure S38. COSY spectrum (region) of **14** (CD_2Cl_2).

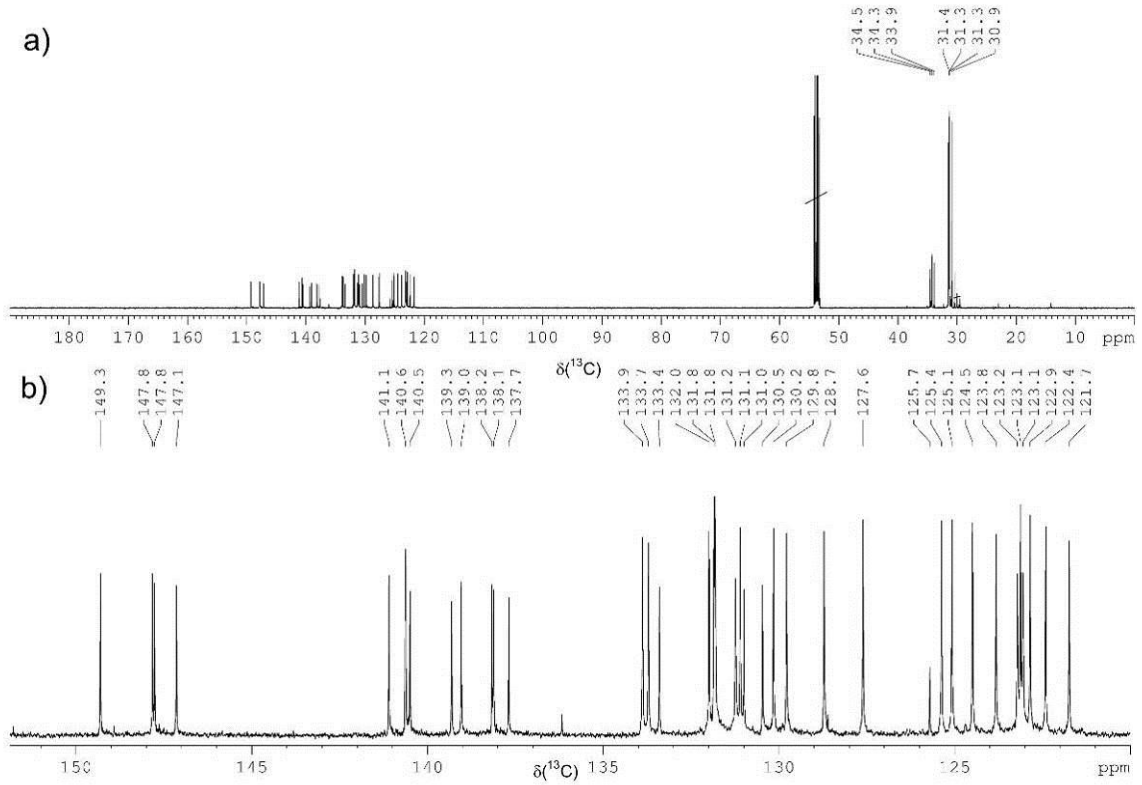

Figure S39. ^{13}C NMR spectra of **14** (125 MHz, CD_2Cl_2): a) overview and b) region of aromatic carbon signals.

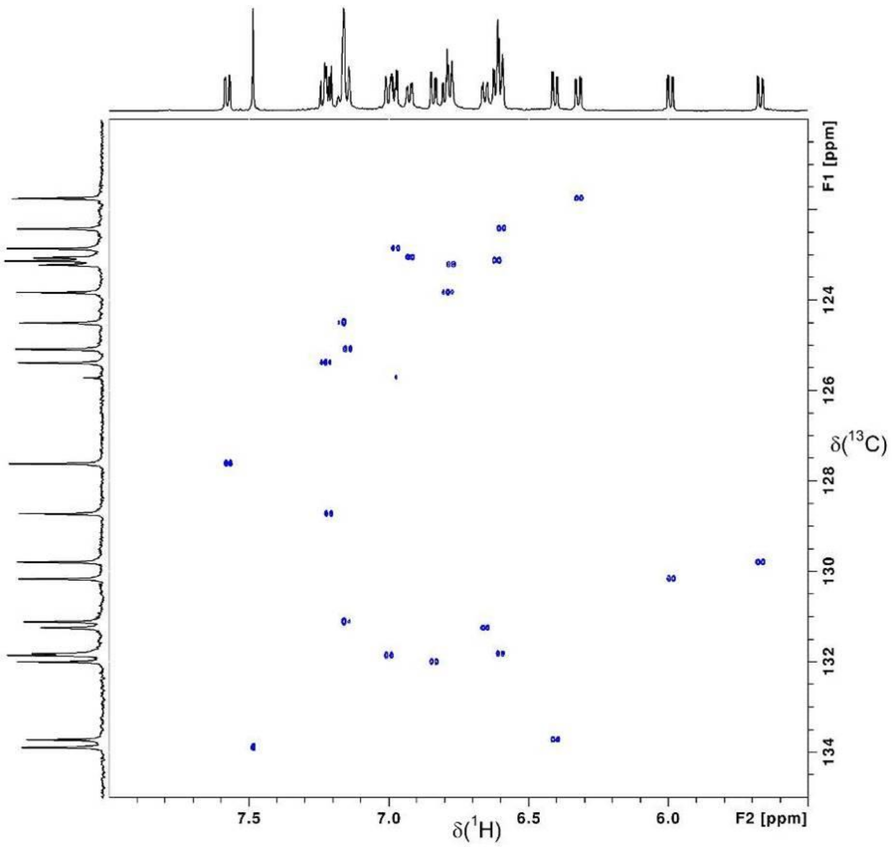

Figure S40. HSQC spectrum (region) of **14** (CD_2Cl_2). The F1 dimension shows the DEPT-135 spectrum.

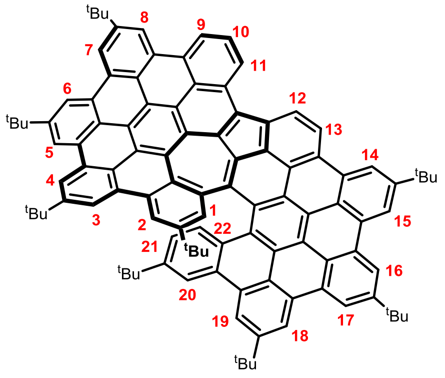

2

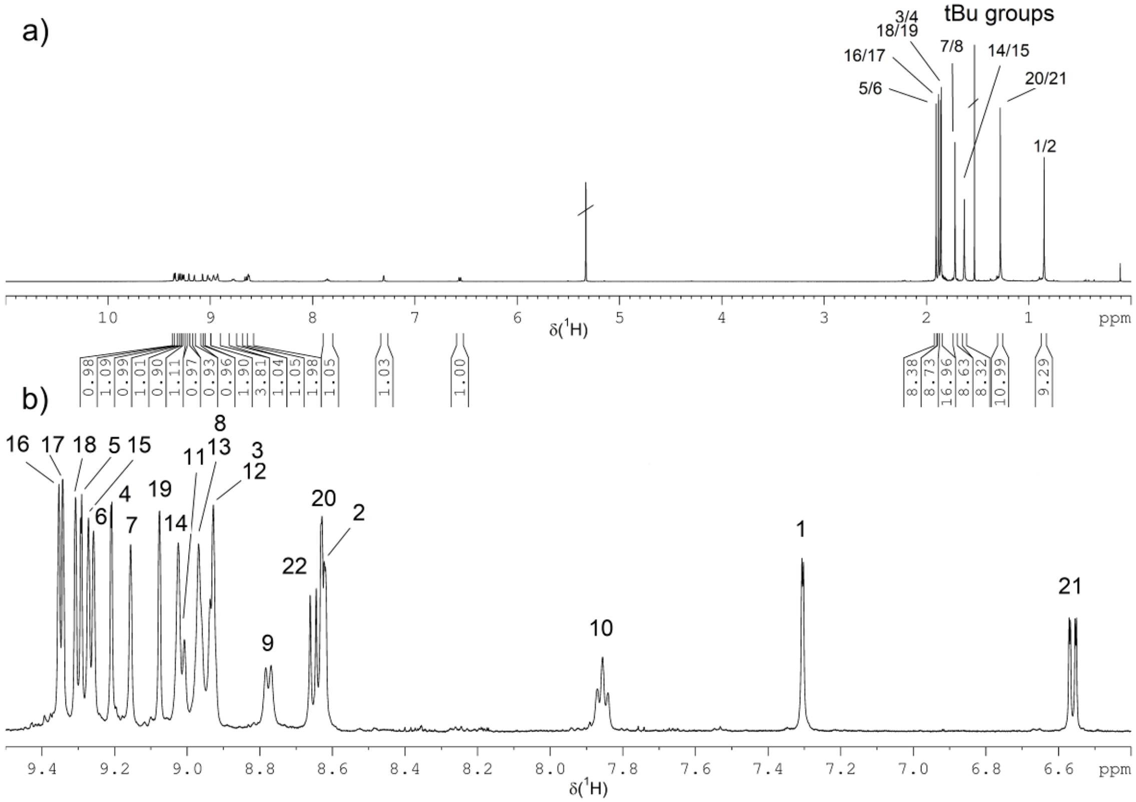

Figure S41 ^{1}H NMR spectrum of **2** (500 MHz, CD_2Cl_2): a) overview and b) enlarged region.

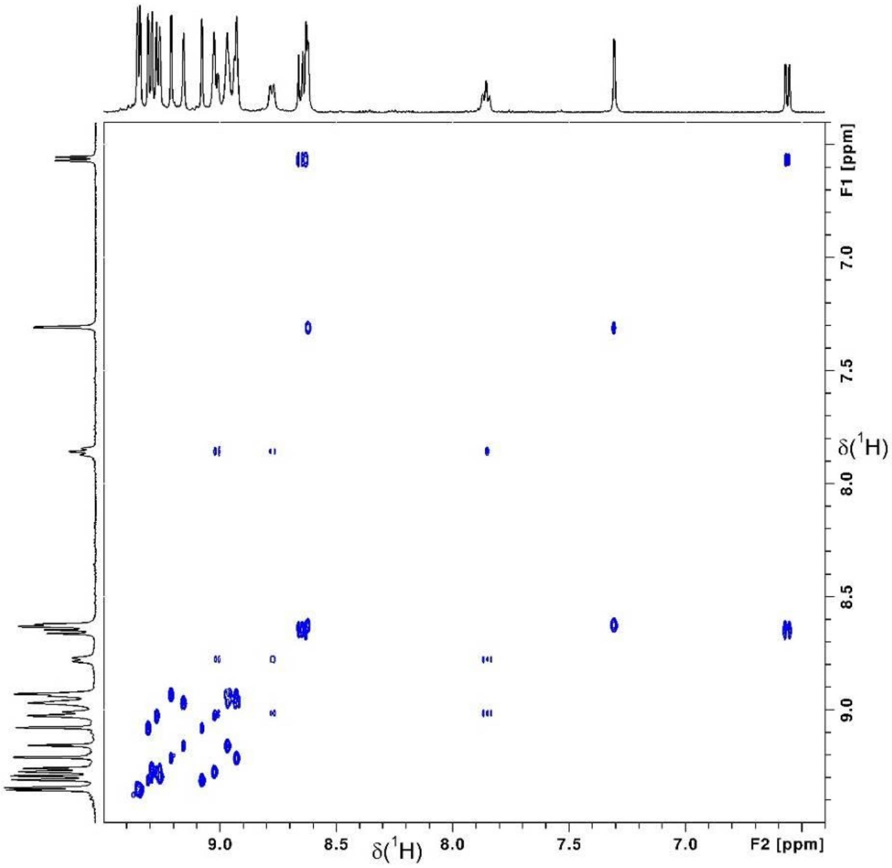

Figure S42. TOCSY spectrum (region) of **2** (CD_2Cl_2).

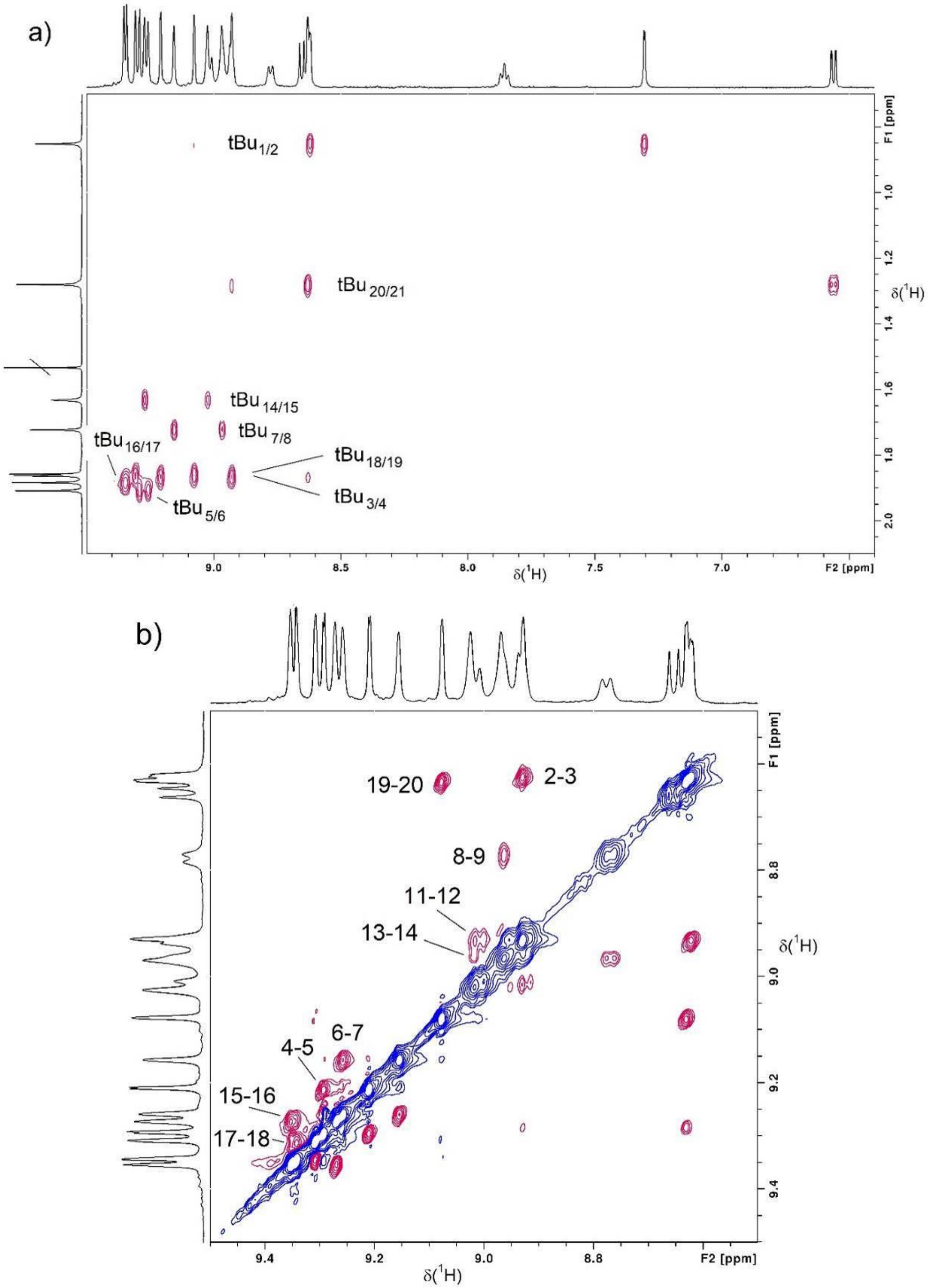

Figure S43. ROESY spectrum (regions) of **2** (CD_2Cl_2) showing the correlations of a) *tert*-butyl groups to aromatic protons and b) aromatic protons (no ROESY correlations observed for H_1, H_{10} and H_{21}).

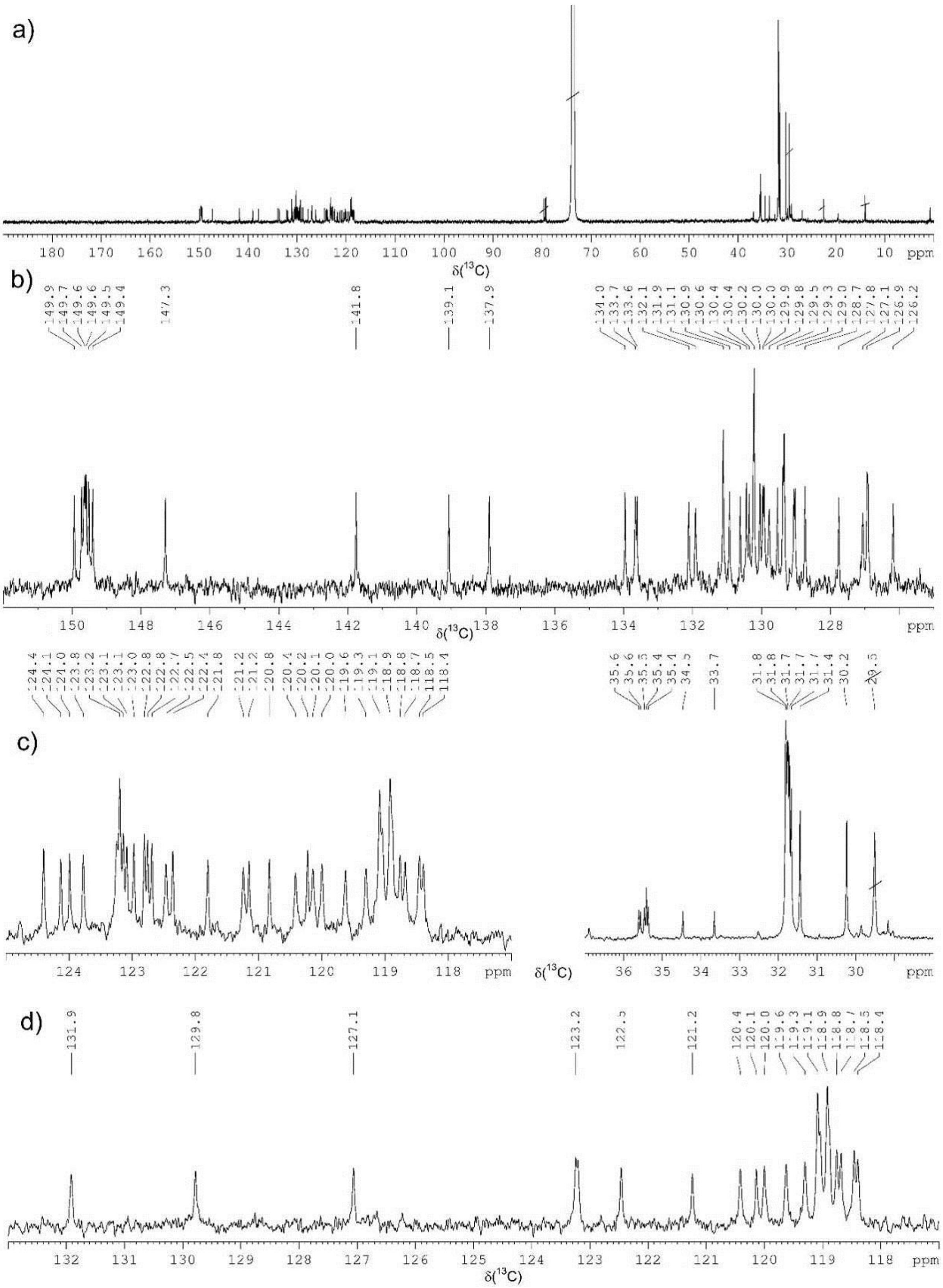

Figure S44. ^{13}C NMR spectra of **2** (125 MHz, $C_2D_2Cl_4$): a) overview, b), c) enlarged regions and d) DEPT-135 spectrum showing only aromatic CH signals.

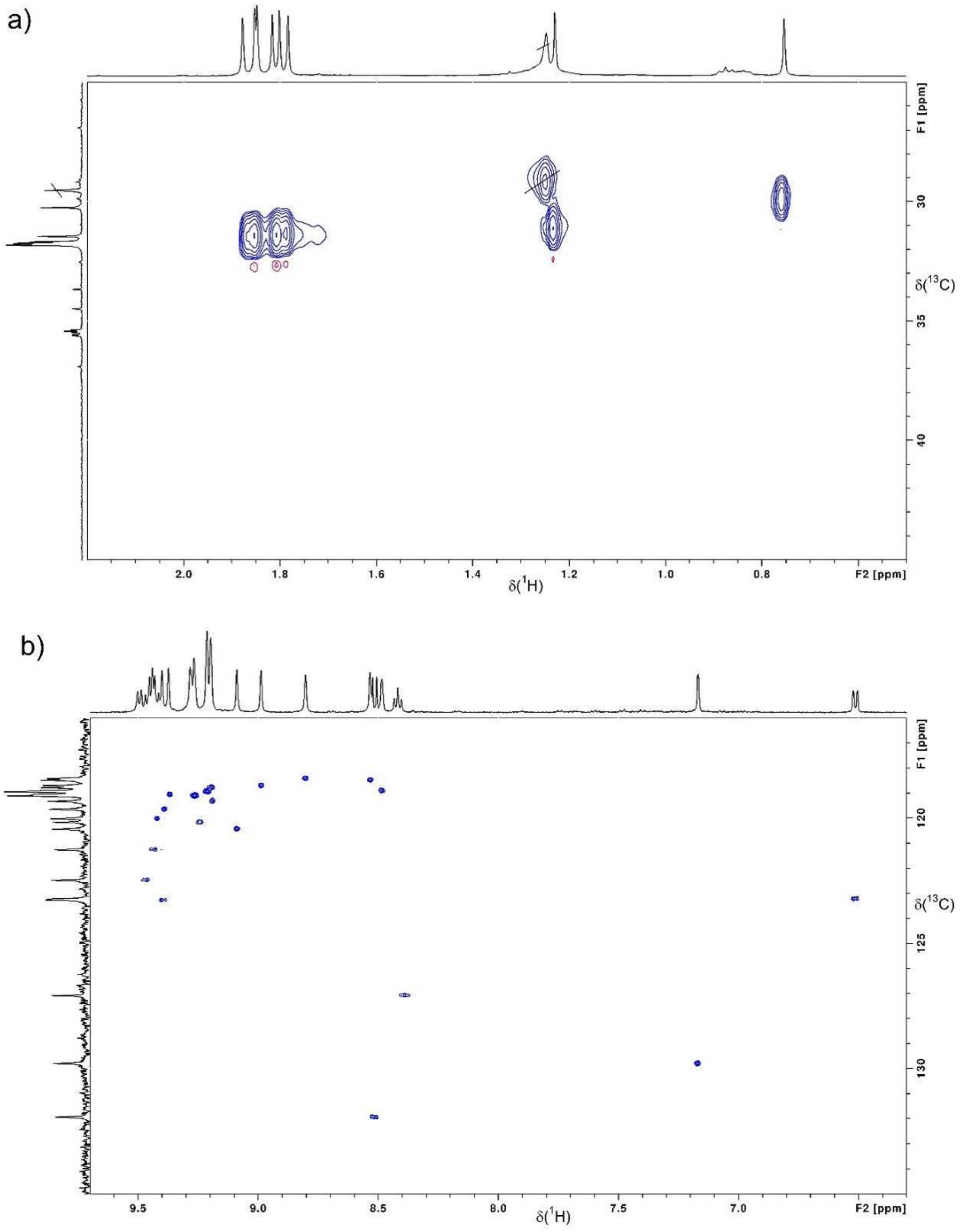

Figure S45. HSQC spectrum (region) of **2** showing the correlations of a) *tert*-butyl and b) aromatic protons ($C_2D_2Cl_4$). The F1 dimension of b) shows the DEPT-135 spectrum (comp. Fig. S44d).

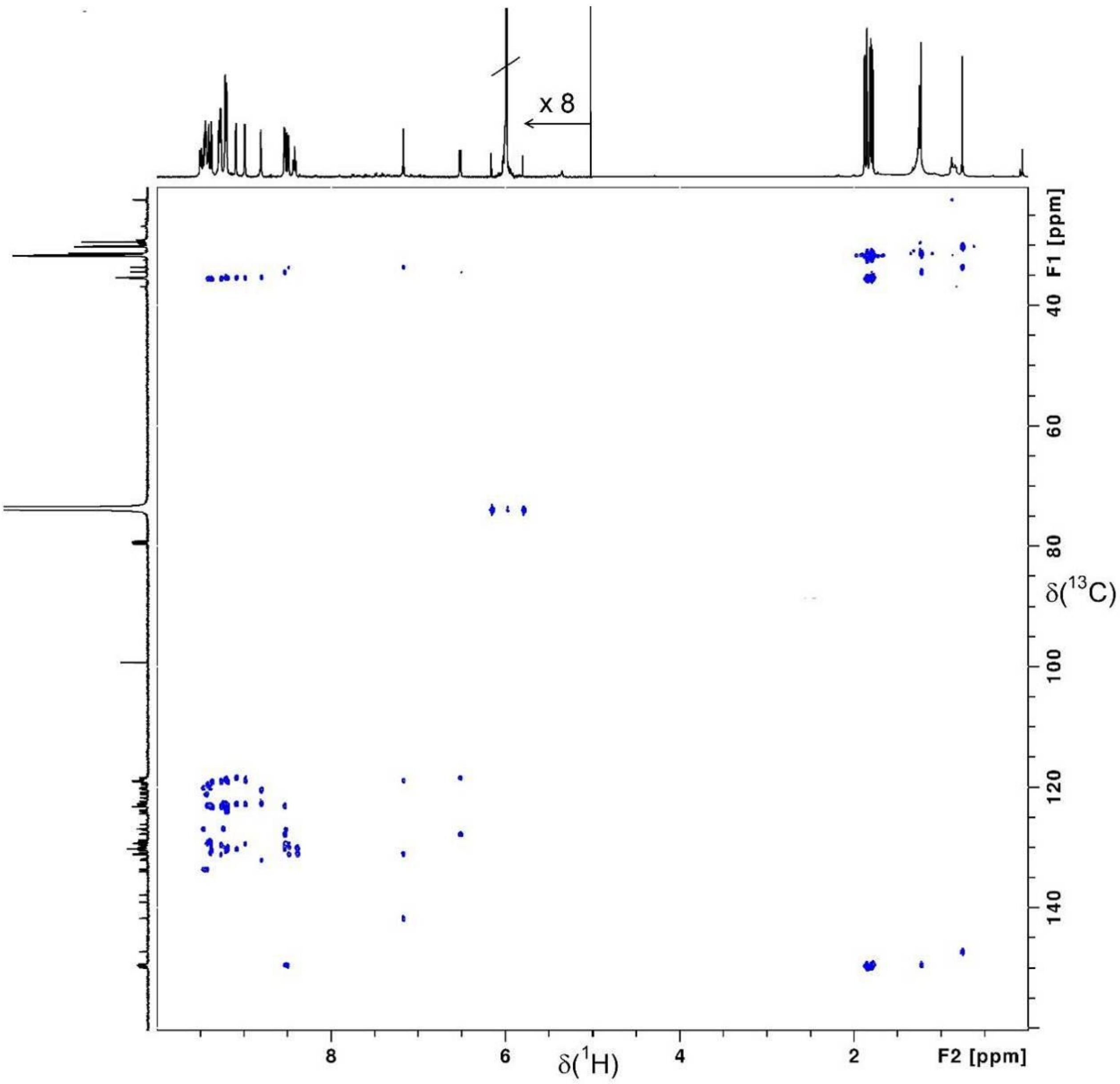

Figure S46. HMBC spectrum of **2** ($C_2D_2Cl_4$). The intensity of the 5 – 10 ppm region is increased by a factor of 8 relative to the 0 – 5 ppm region.

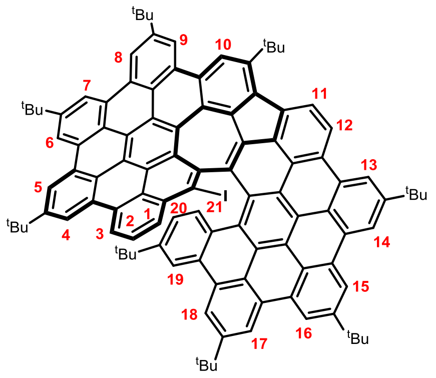

3

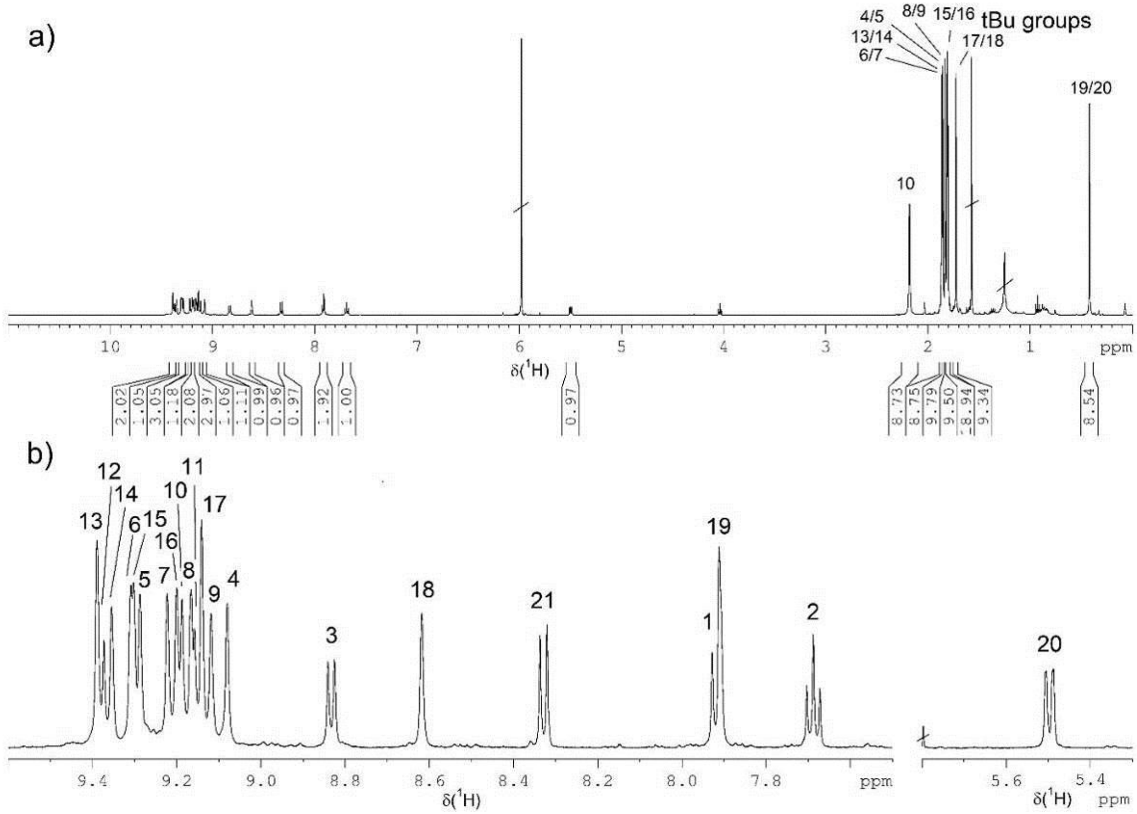

Figure S47. ^{1}H NMR spectrum of **3** (500 MHz, $C_2D_2Cl_4$): a) overview and b) enlarged region.

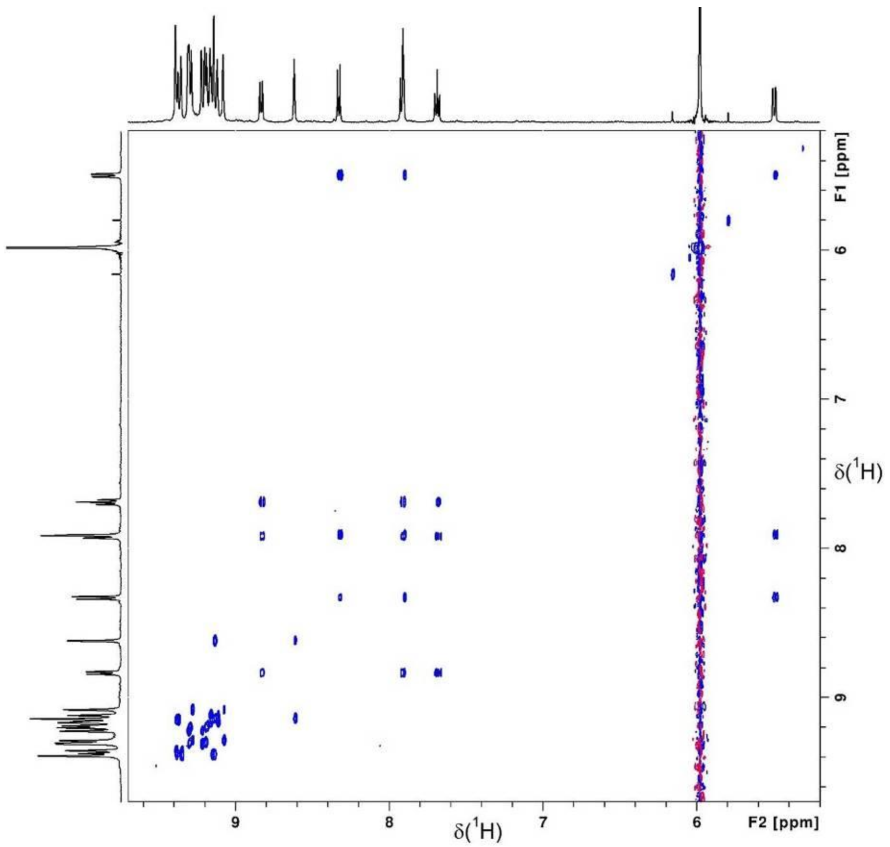

Figure S48. TOCSY spectrum (region) of **3** ($C_2D_2Cl_4$).

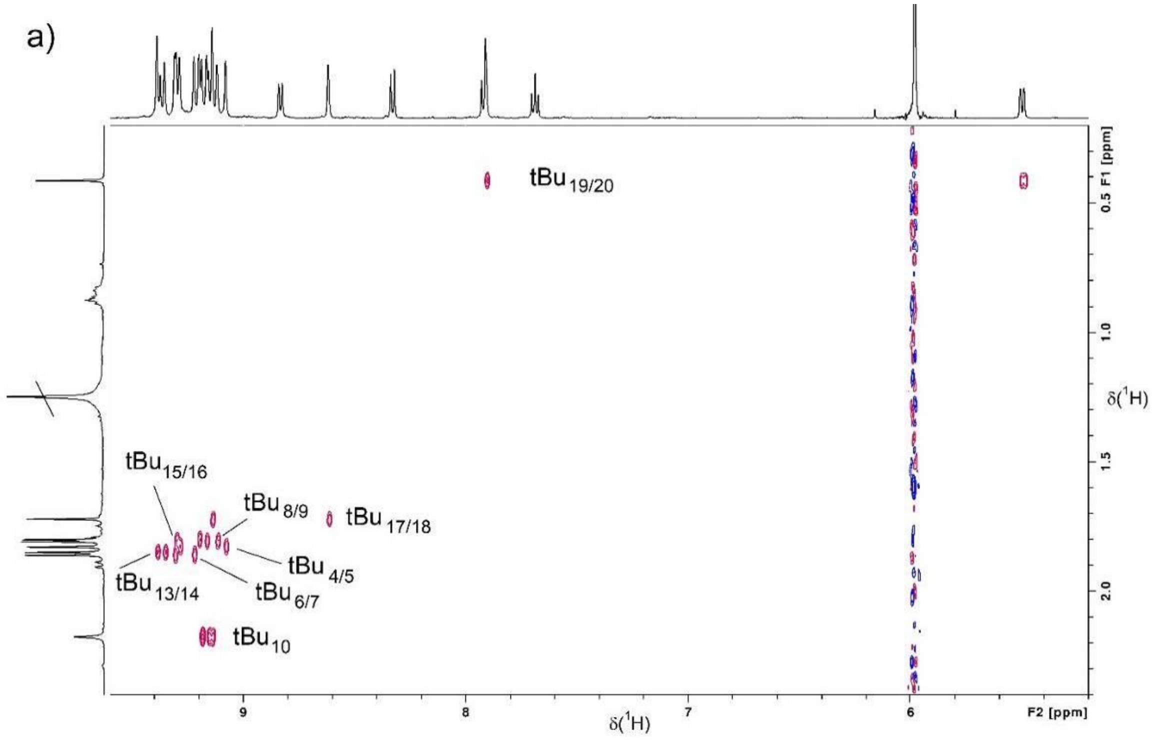

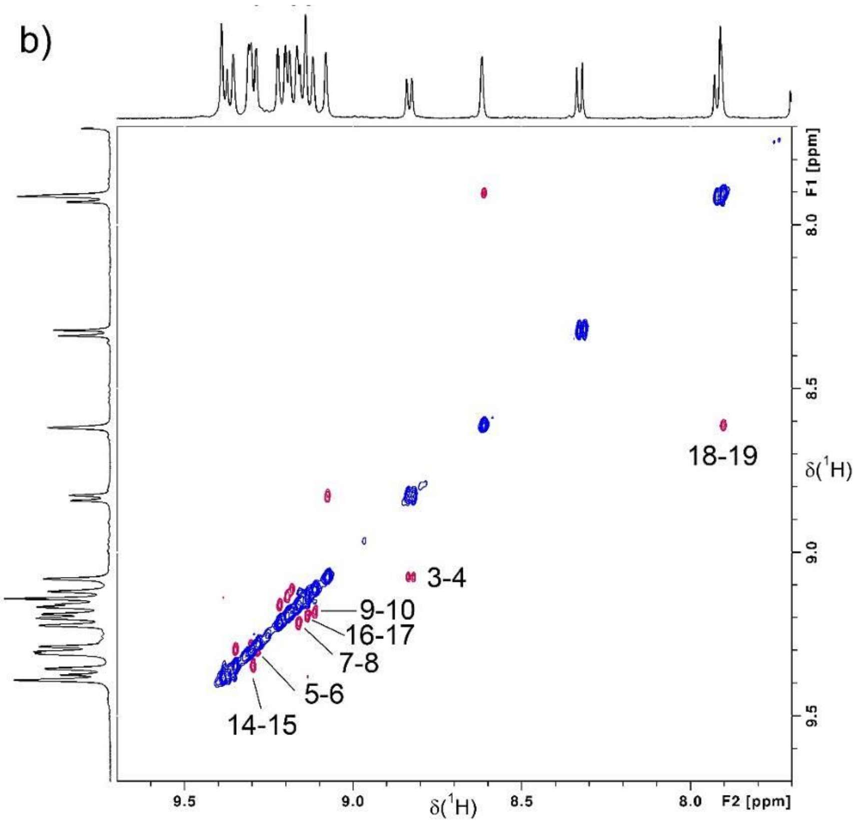

Figure S49. ROESY spectrum (regions) of **3** ($C_2D_2Cl_4$), showing the correlations of a) *tert*-butyl groups to aromatic protons and b) of different aromatic protons (no ROESY correlations observed for H_2 and H_{21}). The H_{12}-H_{13} correlation is not resolved due to the small chemical shift difference (0.07 ppm).

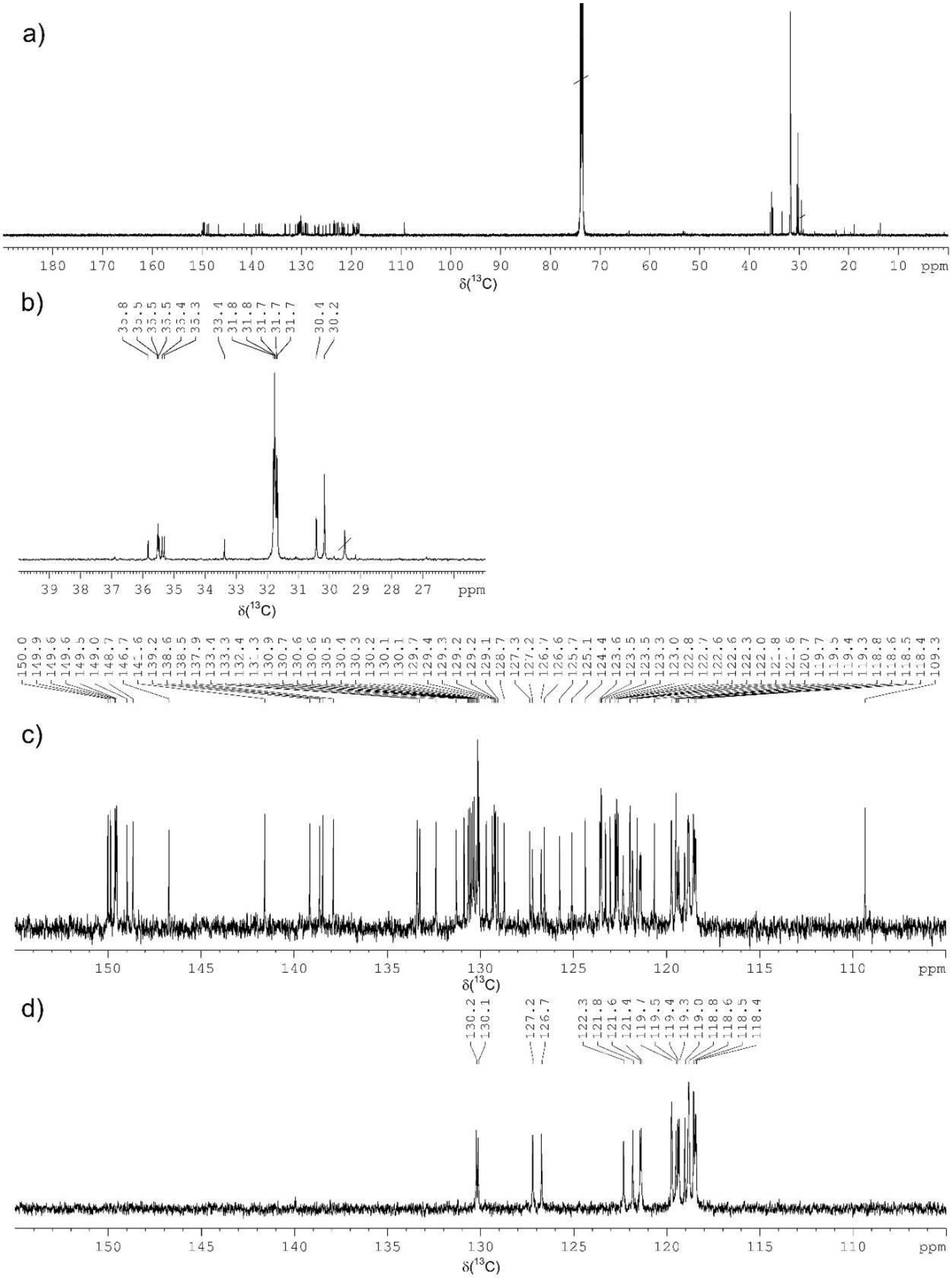

Figure S50. ^{13}C NMR spectra of **3** (125 MHz, $C_2D_2Cl_4$): a) overview, b), c) enlarged regions and d) DEPT-135 spectrum showing only aromatic CH signals.

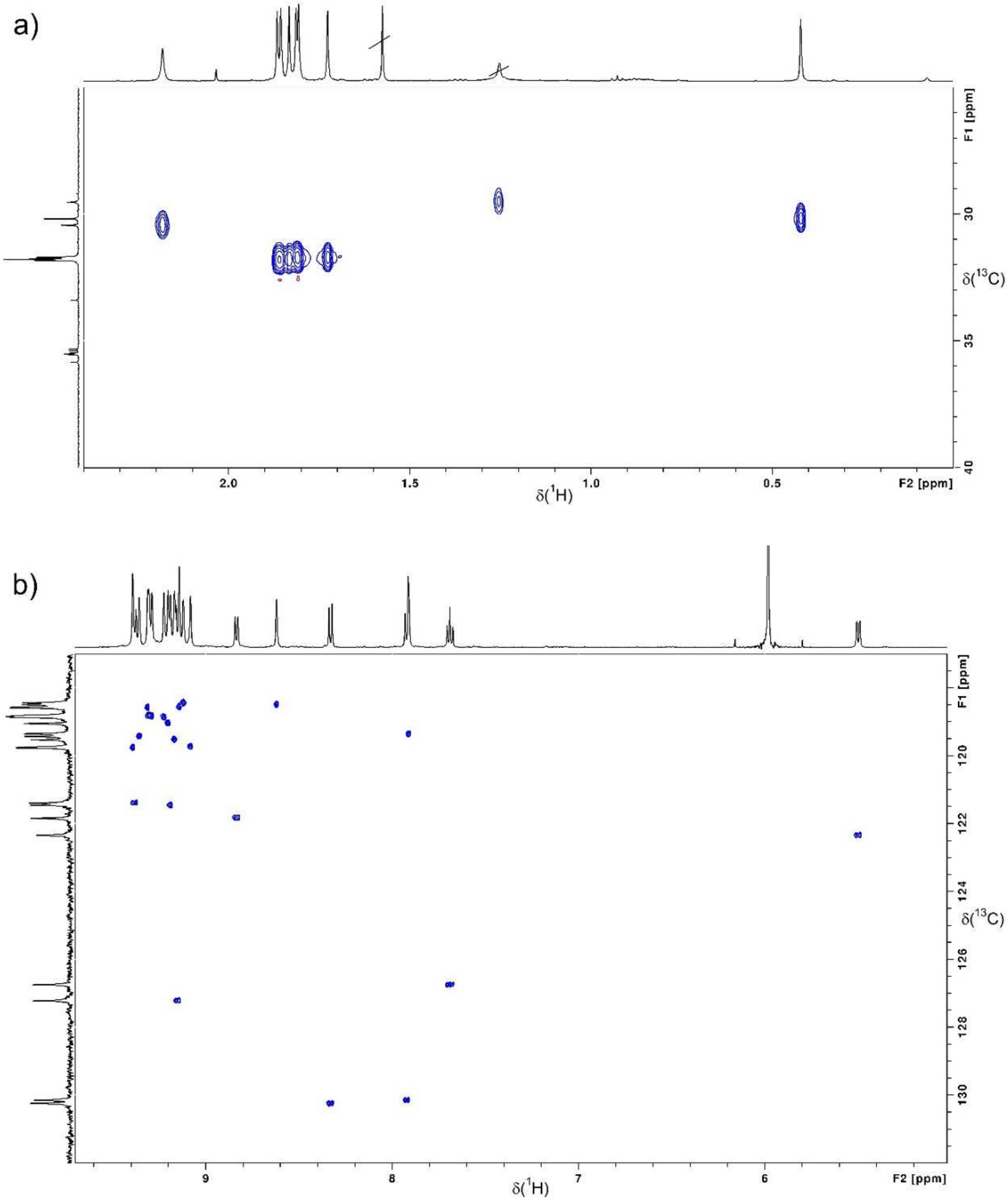

Figure S51. HSQC spectrum (region) of **3** showing the correlations of a) *tert*-butyl and b) of aromatic protons ($C_2D_2Cl_4$). The F1 dimension of b) shows the DEPT-135 spectrum (comp. Fig. S50d).

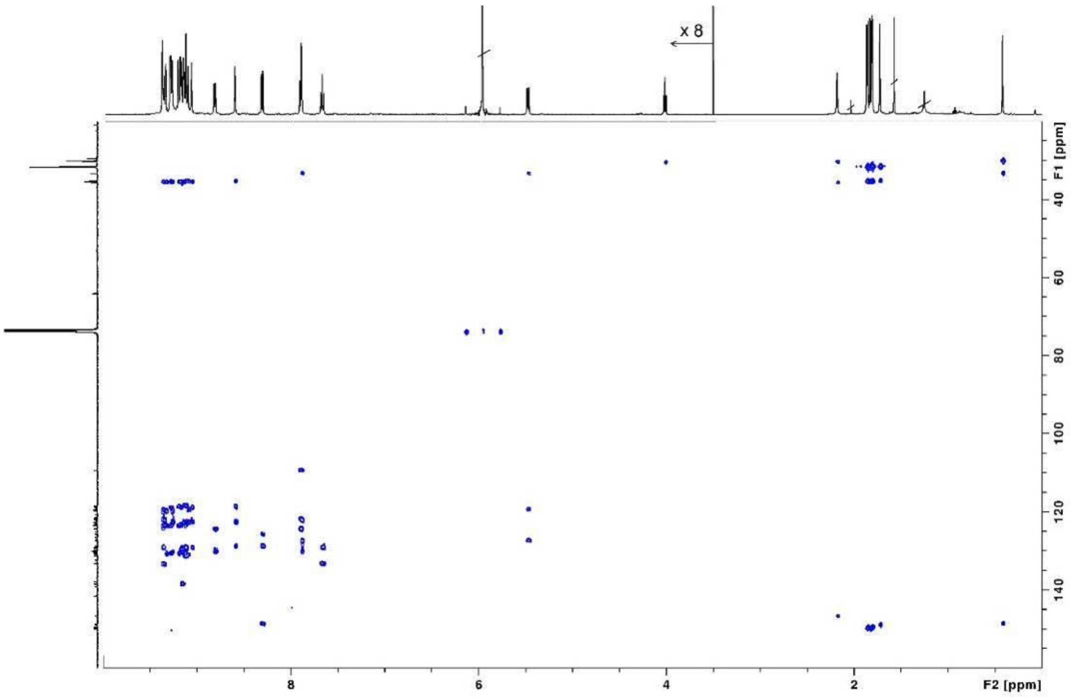

Figure S52. HMBC spectrum of **3** ($C_2D_2Cl_4$). The intensity of the 3.5 – 10 ppm region is increased by a factor of 8 relative to the 0 – 3.5 ppm region.

10. High Resolution Mass Spectra (HR-MS)

Compound Table

Compound Label	RT	Mass	Abund	Formula	Tgt Mass	Diff (ppm)
Cpd 1: C31 H34 Si	1.25	434.2423	724762	C31 H34 Si	434.24298	-1.56

MS Zoomed Spectrum

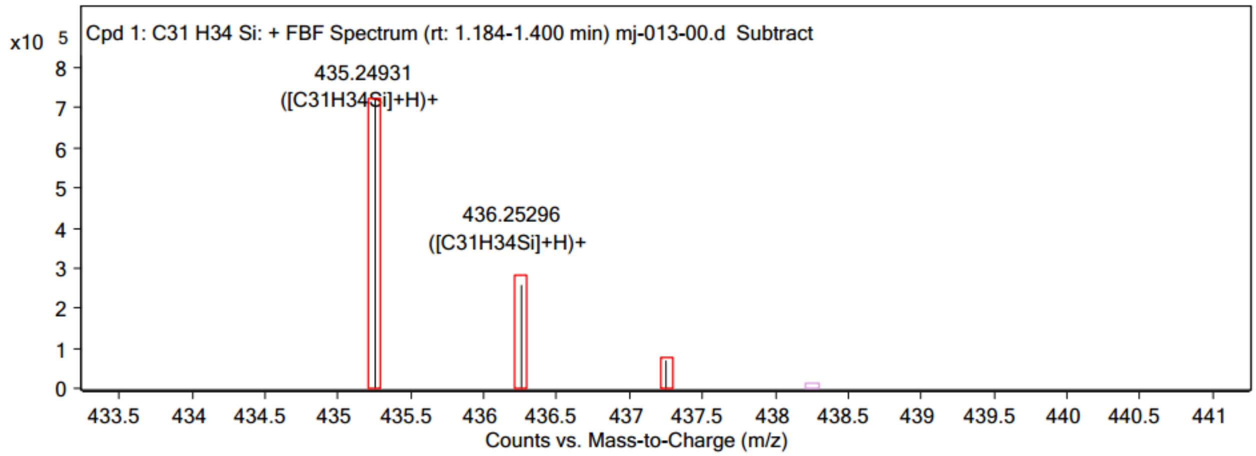

MS Spectrum Peak List

m/z	z	Abund	Formula	Ion
435.24931	1	724761.88	C31H34Si	(M+H)+
436.25296	1	258843.39	C31H34Si	(M+H)+
437.25361	1	67239.59	C31H34Si	(M+H)+

--- End Of Report ---

Figure S53. HR-ESI mass spectrum of compound **5**.

Compound Table

Compound Label	RT	Mass	Abund	Formula	Tgt Mass	Diff (ppm)
Cpd 1: C44 H26	1.186	554.20202	41736	C44 H26	554.20345	-2.59

MS Zoomed Spectrum

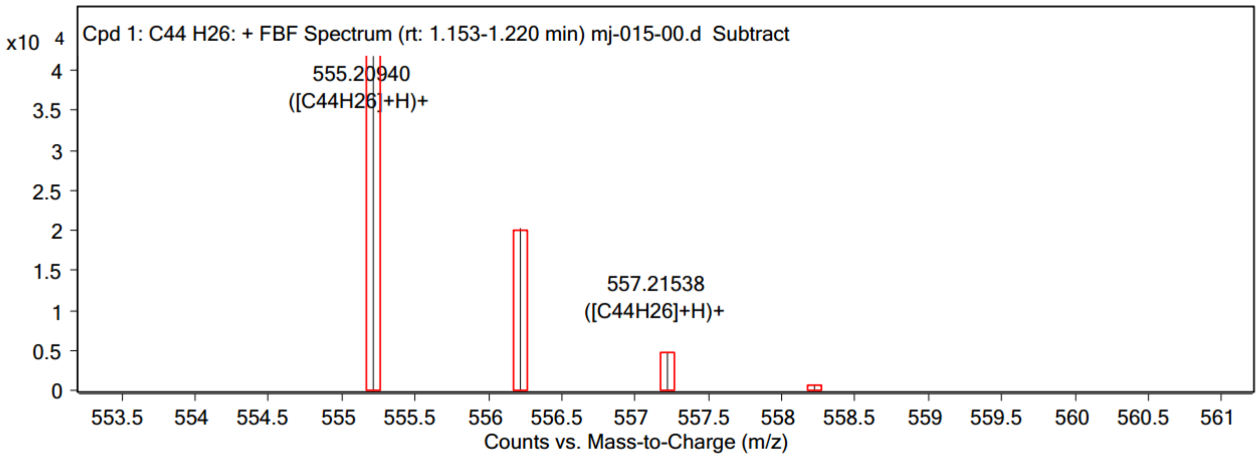

MS Spectrum Peak List

m/z	z	Abund	Formula	Ion
555.2094	1	41736.13	C44H26	(M+H)+
556.21259	1	20239.24	C44H26	(M+H)+
557.21538	1	4669.95	C44H26	(M+H)+
558.21988	1	738.68	C44H26	(M+H)+

--- End Of Report ---

Figure S54. HR-ESI mass spectrum of compound **7**.

Compound Table

Compound Label	RT	Mass	Abund	Formula	Tgt Mass	Diff (ppm)
Cpd 1: C44 H24 I2	1.276	805.99543	45867	C44 H24 I2	805.99674	-1.63

MS Zoomed Spectrum

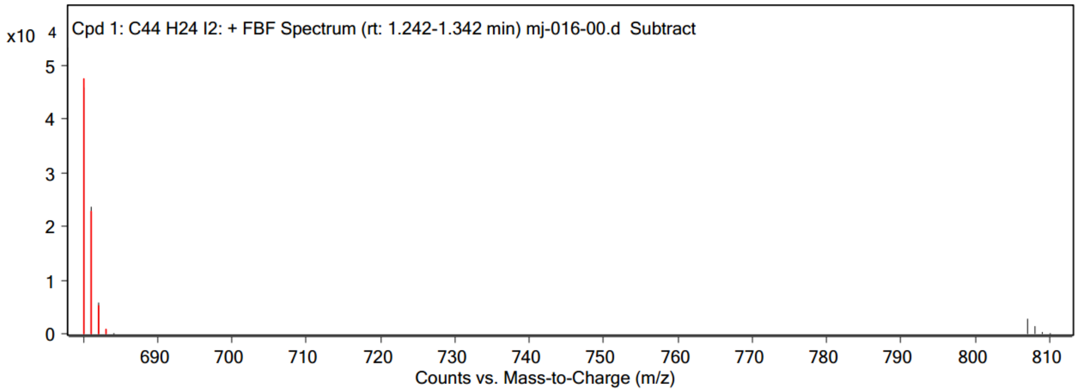

MS Spectrum Peak List

m/z	z	Abund	Formula	Ion
680.09841	1	45867.48	C44H24I2	(M+H)+[-I]
681.10166	1	23667.32	C44H24I2	(M+H)+[-I]
682.10478	1	5912.42	C44H24I2	(M+H)+[-I]
683.10827	1	956.69	C44H24I2	(M+H)+[-I]
684.11277	1	114.84	C44H24I2	(M+H)+[-I]
807.00073	1	2723.8	C44H24I2	(M+H)+
808.00452	1	1314.32	C44H24I2	(M+H)+
809.00776	1	260	C44H24I2	(M+H)+
810.00308	1	49.31	C44H24I2	(M+H)+

Figure S55. HR-ESI mass spectrum of compound **8**.

Compound Table

Label	Tgt Score	Mass Error (ppm)	Tgt Formula	Obs. RT	Ref. Mass	Obs. Mass
Cpd 1: C108 H114	99.62	-0.41	C108 H114	1.54	1410.89205	1410.89148

Obs. *m/z*	Obs. RT	Obs. Mass	Tgt Formula	Tgt Mass	Tgt Mass Error (ppm)	RT Diff.	Find Cpds Algorithm
1412.90239	1.54	1410.89148	C108 H114	1410.89205	-0.41	Find By Formula	

MS Zoomed Spectrum

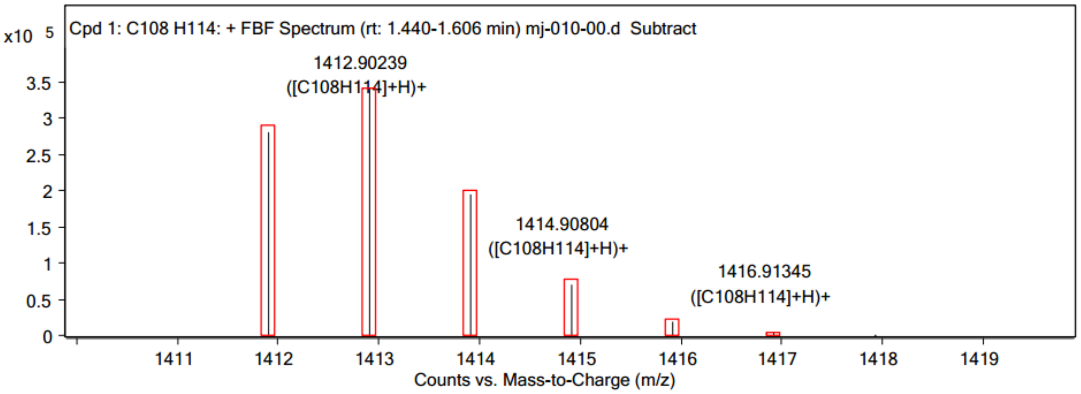

MS Spectrum Peak List

Obs. *m/z*	Charge	Abund	Formula	Ion/Isotope
1411.89883	1	280055.81	C108H114	(M+H)+
1412.90239	1	341349.5	C108H114	(M+H)+
1413.90552	1	194885.53	C108H114	(M+H)+
1414.90804	1	70195.05	C108H114	(M+H)+
1415.91062	1	18694.11	C108H114	(M+H)+
1416.91345	1	4497.7	C108H114	(M+H)+
1417.91627	1	719.52	C108H114	(M+H)+

--- End Of Report ---

Figure S56. HR-ESI mass spectrum of compound **12**.

Compound Table

Compound Label	RT	Mass	Abund	Formula	Tgt Mass	Diff (ppm)
Cpd 1: C108 H112 I2	1.209	1662.6822	3250	C108 H112 I2	1662.68534	-1.89

MS Zoomed Spectrum

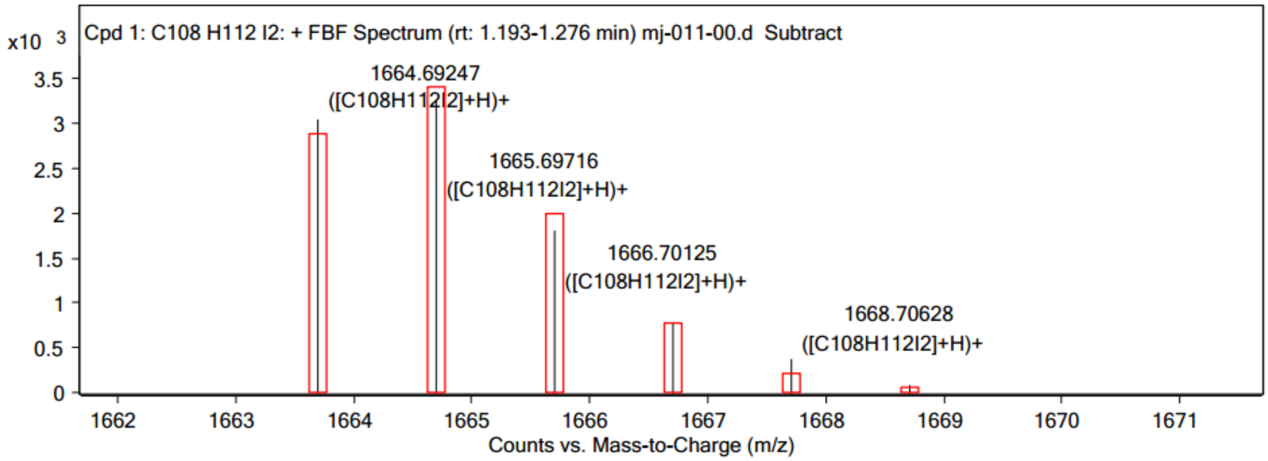

MS Spectrum Peak List

m/z	z	Abund	Formula	Ion
1663.68927	1	3046.98	C108H112I2	(M+H)+
1664.69247	1	3249.97	C108H112I2	(M+H)+
1665.69716	1	1801.8	C108H112I2	(M+H)+
1666.70125	1	775.34	C108H112I2	(M+H)+
1667.7005	1	371.05	C108H112I2	(M+H)+
1668.70628	1	84.24	C108H112I2	(M+H)+

--- End Of Report ---

Figure S57. HR-MALDI-TOF mass spectrum of compound **13**.

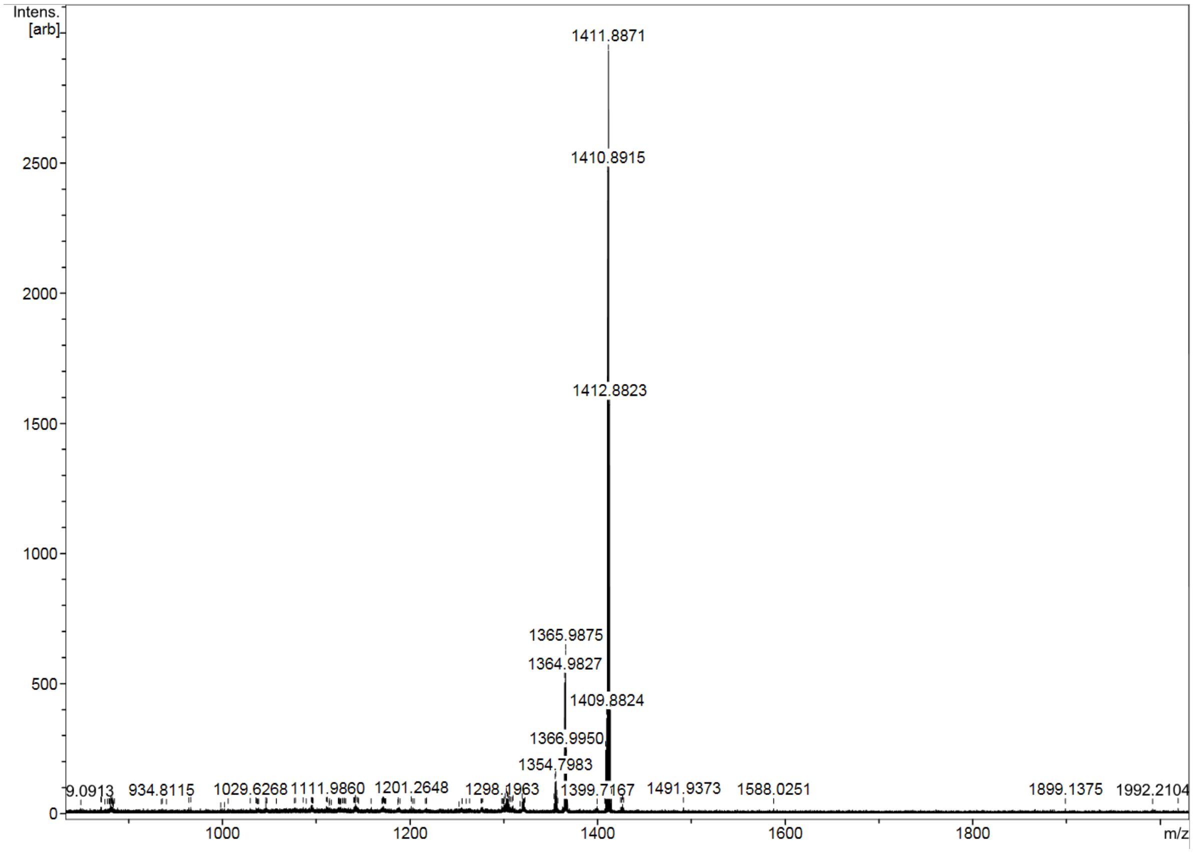

Figure S58. HR-MALDI-TOF mass spectrum of compound **14**.

11. References

[1] T. Ide, S. Sakamoto, D. Takeuchi, K. Osakada, S. Machida, *J. Org. Chem.* **2012**, *77*, 4837-4841.

[2] K. W. Bentley, Z. A. de los Santos, M. J. Weiss, C. Wolf, *Chirality*, **2015**, 27, 700.

[3] D. J. Gregg, C. M. A. Ollagnier, C. M. Fitchett, S. M. Draper, *Chem. Eur. J.* **2006,** 12, 3043.

[4] Gaussian 09, Revision D.01, M. J. Frisch, G. W. Trucks, H. B. Schlegel, G. E. Scuseria, M. A. Robb, J. R. Cheeseman, G. Scalmani, V. Barone, B. Mennucci, G. A. Petersson, H. Nakatsuji, M. Caricato, X. Li, H. P. Hratchian, A. F. Izmaylov, J. Bloino, G. Zheng, J. L. Sonnenberg, M. Hada, M. Ehara, K. Toyota, R. Fukuda, J. Hasegawa, M. Ishida, T. Nakajima, Y. Honda, O. Kitao, H. Nakai, T. Vreven, J. A. Montgomery, Jr., J. E. Peralta, F. Ogliaro, M. Bearpark, J. J. Heyd, E. Brothers, K. N. Kudin, V. N. Staroverov, R. Kobayashi, J. Normand, K. Raghavachari, A. Rendell, J. C. Burant, S. S. Iyengar, J. Tomasi, M. Cossi, N. Rega, J. M. Millam, M. Klene, J. E. Knox, J. B. Cross, V. Bakken, C. Adamo, J. Jaramillo, R. Gomperts, R. E. Stratmann, O. Yazyev, A. J. Austin, R. Cammi, C. Pomelli, J. W. Ochterski, R. L. Martin, K. Morokuma, V. G. Zakrzewski, G. A. Voth, P. Salvador, J. J. Dannenberg, S. Dapprich, A. D. Daniels, Ö. Farkas, J. B. Foresman, J. V. Ortiz, J. Cioslowski, and D. J. Fox, Gaussian, Inc., Wallingford CT, **2009**.

[5] D. Geuenich, K. Hess, F. Köhler, R. Herges, *Chem. Rev.* **2005**, 105, 3758.

[6] (a) Z. Chen, C. S. Wannere, C. Corminboeuf, R. Puchta, P. v. R.Schleyer, *Chem. Rev.* **2015**, 105, 3842. (b) H. Fallah-Bagher-Shaidaei, S. S. Wannere, C. Corminboeuf, R., Puchta, P. v. R. Schleyer, *Org. Lett.* **2006**, 8, 863.
